# Interlayer Dzyaloshinskii–Moriya Interaction in Synthetic Ferrimagnets for Spiking Neural Networks

**DOI:** 10.1002/advs.202519110

**Published:** 2026-01-04

**Authors:** Shen Li, Xing Chen, Mouad Fattouhi, Tianxun Huang, Chen Lv, Mark C. H. de Jong, Pingzhi Li, Daoqian Zhu, Xiaoyang Lin, Felipe Garcia‐Sanchez, Eduardo Martinez, Stéphane Mangin, Bert Koopmans, Weisheng Zhao, Reinoud Lavrijsen

**Affiliations:** ^1^ State Key Laboratory of Spintronics Hangzhou International Innovation Institute Beihang University Hangzhou China; ^2^ Department of Applied Physics Eindhoven University of Technology Eindhoven The Netherlands; ^3^ Fert Beijing Institute MIIT Key Laboratory of Spintronics School of Integrated Circuit Science and Engineering Beihang University Beijing China; ^4^ Laboratoire Albert Fert CNRS Thales Université Paris‐Saclay Palaiseau France; ^5^ Department of Applied Physics Universidad De Salamanca Salamanca Spain; ^6^ Institut Jean Lamour UMR CNRS 7198 Université De Lorraine Nancy France

**Keywords:** interlayer Dzyaloshinskii–Moriya interaction, spiking neural network, spin‐orbit torque, synthetic ferrimagnets

## Abstract

Interlayer Dzyaloshinskii–Moriya interaction (IL‐DMI) in synthetic magnetic structures has attracted extensive interest for greatly facilitating deterministic spin‐orbit torque (SOT)‐driven information writing and topologically non‐trivial 3D magnetic Hopfion forming. However, its distinct role in synthetic ferrimagnets (SFi) remains unexplored, where the conjunction of asymmetric magnetic moments and antisymmetric nature of IL‐DMI leads to more diverse spin configurations and applications. Here, we reveal the unidirectional and chiral nature of IL‐DMI in SFi, further unlocking application directions of IL‐DMI in neuromorphic computing. Particularly, the IL‐DMI‐induced effective field increases approximately twentyfold while interacting with two asymmetric antiparallel‐aligned moments, greatly facilitating future IL‐DMI detection. Unlike previous digital‐like switching, we find that the interplay of IL‐DMI, SOT, and thermal effect gives rise to an analog‐like switching behavior. Leveraging this, we develop an SOT‐based non‐probabilistic leaky‐integrate‐fire neuron device utilizing the micromagnetic analog‐like switching model. Compared to probabilistic neurons, this provides a hardware support Spiking neural network, interlayer Dzyaloshinskii–Moriya interaction, spin‐orbit torque, synthetic ferrimagnetsfor ultralow power, high‐sparsity, and high‐accuracy spiking neural networks.

## Graphical abstract

This work introduces a groundbreaking integration of asymmetric magnetic structures (synthetic ferrimagnets) and antisymmetric magnetic interaction (interlayer Dzyaloshinskii–Moriya interaction) for the first time. It addresses the critical challenge of IL‐DMI detection and shows the discovery of unprecedented analog‐like spin‐orbit torque switching. The analog switching naturally matches the neuron characteristics in spiking neural networks, promising for ultralow‐power, high‐sparsity, and high‐accuracy artificial intelligence hardware.

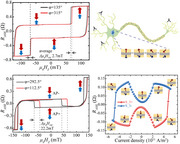

## 1 Introduction

Synthetic antiferromagnetically‐coupled structures have become indispensable in spintronic devices due to their combined advantages of both ferromagnets (high readability and writability) and antiferromagnets (high scalability, thermal stability, and ultrafast magnetization dynamics) [1, 2]. Consisting of two or more ferromagnetic (FM) layers separated by spacers, synthetic antiferromagnetically‐coupled structures have evolved rapidly toward various applications such as magnetic random access memory (MRAM) [3], racetrack [4], spin‐torque oscillators [5], magnetic synapses [6], etc. In these structures, the antiferromagnetic (AFM) coupling is achieved through a symmetric interlayer exchange interaction (IEI), i.e., Ruderman–Kittel–Kasuya–Yosida (RKKY) interaction, where the interaction between spins in the FM layers and electrons in the spacers induces a spin‐dependent Friedel‐like spatial oscillation of the spin density [7, 8, 9]. Consequently, the RKKY interaction leads to a symmetric FM/AFM coupling depending on the thickness of the spacer [10, 11]. Recently, a new type of IEI—the interlayer Dzyaloshinskii–Moriya interaction (IL‐DMI)—has been theoretically predicted [12] and experimentally verified [13, 14]. IL‐DMI represents an antisymmetric counterpart to the RKKY interaction, leading to a chiral coupling, where the magnetizations tend to align perpendicularly in a clockwise/counterclockwise manner. Following this, IL‐DMI has garnered numerous interests due to its ability to break the symmetry required for deterministic spin‐orbit torque (SOT)‐driven information writing [15, 16] and promote topologically non‐trivial 3D spin textures such as hopfions. [1, 17, 18] Besides, other intriguing spin phenomena based on IL‐DMI have also been observed, including 360° domain wall rings [19], overall scalar spin chirality preservation [20] and asymmetric domain wall expansion [21, 22].

Synthetic antiferromagnetically‐coupled structures can be classified into synthetic antiferromagnets (SAFs) and synthetic ferrimagnets (SFi) based on whether the magnetic moments of the upper and lower layers are symmetric. The above extensive studies have explored IL‐DMI in symmetric SAFs. However, for asymmetric SFi whose net magnetic moment is not completely canceled, the role of antisymmetric IL‐DMI has not been unraveled. The combination of asymmetric structures and antisymmetric interactions can create more diverse spin arrangements and applications. In this work, we present both the experimental and simulation demonstrations of the field‐induced and SOT‐induced asymmetric magnetization switching behaviors of SFi with perpendicular magnetic anisotropy under the action of IL‐DMI, further demonstrating its application potential in neuromorphic computing. We demonstrate that IL‐DMI behaves as an effective in‐plane field with unidirectional and chiral characteristics during the magnetization switching of SFi. The interaction of this effective field with unbalanced antiparallel‐aligned moments in the SFi significantly increases the switching asymmetries in the hysteresis loops with an extra in‐plane field. The approximately 20‐fold field amplification is caused by the changed IL‐DMI energy contribution in the Arrhenius law and is explained by theoretical calculations, greatly facilitating future IL‐DMI detection. Progressing toward the influence of IL‐DMI in SOT switching, we measure both the transient state of the SFi during the application of the current pulse and the steady state of the SFi after the current pulse. In addition to field‐free switching, we find that the interplay of IL‐DMI and SOT can achieve an analog‐like (continuous) switching behavior with an asymmetric critical switching current distribution, which benefits future design of SOT‐based neuromorphic devices [23]. Corresponding micromagnetic simulations are in agreement with experimental results, further elucidating the role of IL‐DMI in hysteresis loops and SOT switching of SFi. Furthermore, a first SOT‐based deterministic leaky‐integrate‐fire (LIF) neuron device for spiking neural network (SNN) is developed utilizing the micromagnetic analog‐like switching model, and an accuracy of 92.5% in Modified National Institute of Standards and Technology (MNIST) handwritten digit recognition is achieved. Compared to probabilistic LIF neurons, this device eliminates the need for multiple iterations and averaging computations, providing application possibilities for ultralow‐power, high‐sparsity, and high‐accuracy neural networks. This work elucidates the unique role of IL‐DMI in the magnetization switching process and fills the gap in SOT application toward neuromorphic computing.

## 2 Results

### 2.1 Asymmetric Magnetization Switching Behaviors of SFi with IL‐DMI

**FIGURE 1:**
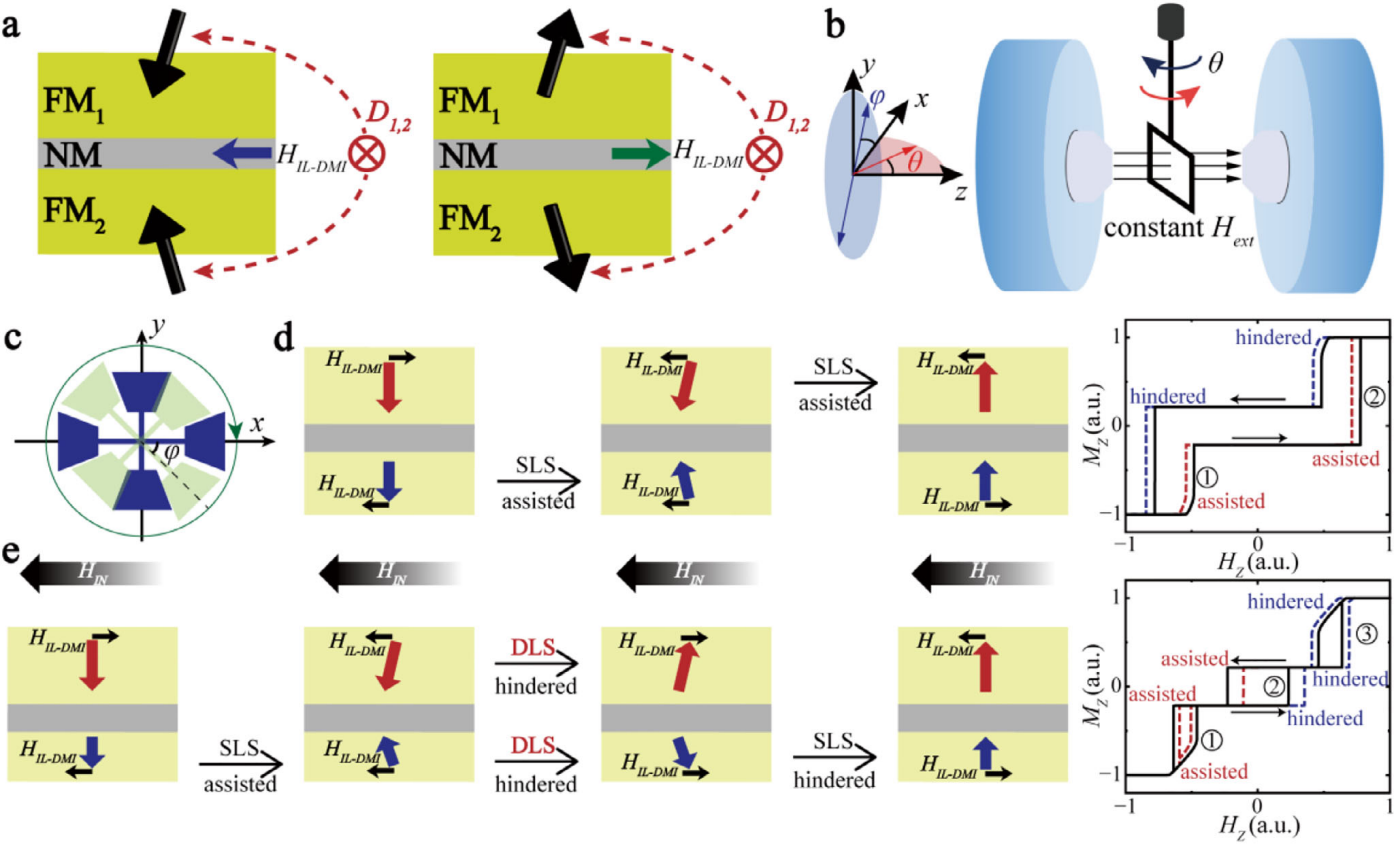
Schematic illustrations of the chiral exchange bias and field‐reversal symmetry breaking. (a) The exchange bias field of the IL‐DMI is opposite under two different antiparallel arrangements with the same chirality. The black arrows represent the magnetization direction of the FM layers. (b,c) The measurement schematic of IL‐DMI under a constant field. The field is set to a value which is slightly larger than the out‐of‐plane saturation field. (b) For each Hall bar with a specific angle *φ* in the *x‐y* plane, the magnetization switching of the SFi is measured by rotating the sample angle *θ* in the *x‐z* plane, where *H_Z_* = *H_ext_* *cos*θ and *H_IN_* = *H_ext_* *sin*θ. (c) The Hall bar is located in the *x‐y* plane and rotates at an angle of *φ* with a step of 22.5°. The angle rotating process in (b) is repeated for every different *φ*. (d and e) Schematics of asymmetric switching of SFi and hysteresis loop changes due to IL‐DMI and an additional IP field in two‐step switching (d) and three‐step switching (e) cases. The red and blue arrows indicate magnetizations of the top and bottom FMs, respectively. The chiral exchange bias *H_IL‐DMI_* breaks the inversion symmetry between up‐to‐down (U–D) and down‐to‐up (D–U) switching polarities in the presence of *H_IN_*. The right sides show the corresponding hysteresis loop changes, and the numbers indicate the switching steps.

We start by developing the necessary concepts to clearly identify the effect of IL‐DMI. In general, the magnetization reversal in SAFs is invariant under the inversion of the magnetic field direction. However, this field‐reversal invariance is broken when there is IL‐DMI in the SAF structure. Figure 1a shows the most prominent feature of IL‐DMI during SAF magnetization switching: chiral exchange bias. The bias field *H_IL‐DMI_* is opposite under two opposite antiparallel arrangements of the SAF. To measure such a variable‐direction exchange bias, we apply a constant‐direction IP magnetic field *H_IN_* during the hysteresis loop switching. Accordingly, the magnetization switching process will either be assisted or hindered depending on the relative direction of *H_IN_* and *H_IL‐DMI_*. Figure 1b,c shows the measurement schematic of IL‐DMI with anomalous Hall effect (AHE) under a constant field *H_ext_*. For a Hall bar with each specific angle *φ* in the *x‐y* plane, the magnetization switching process is measured by rotating the sample angle *θ* clockwise or counterclockwise in the *x‐z* plane. During the rotation of *θ*, the magnetic component perpendicular to the sample plane *H_Z_* = *H_ext_* *cos*θ drives the switching and the IP component *H_IN_* = *H_ext_* *sin*θ helps to define the IP switching direction. Furthermore, we obtain the AHE loops at each angle *φ* and compare them. The sign and magnitude of the effective IL‐DMI field can be extracted from the asymmetric switching behavior driven by *H_ext_* with *H_IN_* along different directions (see details in Section S1).

Depending on the degree of imbalance between the two magnetic moments of the upper and lower layers, the SFi magnetization switching will appear as either two‐step switching (Figure 1d) or three‐step switching (Figure 1e), respectively. When the magnetic moment difference between the two layers is small, the magnetization switching of SFi is similar to that of SAF. Each layer switches only once, and it requires two steps to complete the switching. The IL‐DMI is conventionally measured in a compensated SAF stack with such a two‐step switching process [13, 15, 16, 21, 24, 25, 26, 27]. Figure 1d shows the schematic of asymmetric switching and the corresponding hysteresis loop change of an SFi in a two‐step switching case due to IL‐DMI and an additional IP field component. In this all single‐layer switching (SLS) situation, the hysteresis loop behaves as a two‐step switching. When *H_z_* is ascending, every switching step is assisted since *H_IL‐DMI_* and *H_IN_* are parallel and adding up. Similarly, when *H_z_* is descending, the switching processes will both be hindered. This assist‐assist‐hinder‐hinder behavior will hence appear as an overall shift in the full hysteresis loop. Therefore, the IL‐DMI can be probed by measuring the overall loop shift (see details in Section S2), and the field strength of IL‐DMI can be characterized by this shifting magnitude. Nevertheless, due to the small magnitude of IL‐DMI [14], the shift is usually very small and hard to extract [28]. Moreover, small errors in angle setting due to sample mounting and hysteresis in the rotation stage will also cause small loop shifts, and this cannot be eliminated.

To improve upon the above issue, we propose to use the hysteresis loop of an SFi stack exhibiting a three‐step switching process. Figure 1e shows the schematic of asymmetric switching and the corresponding hysteresis loop change of SFi due to IL‐DMI when there is a magnetization rearranging process due to the imbalance of the magnetic moments of the two layers. When there is a large thickness difference between the upper and lower FM layers, an extra step in the hysteresis loop is observed as the thicker layer tends to align with the external magnetic field due to the Zeeman energy gain. However, this requires the other layer to also switch as the RKKY interaction is still strong enough to stabilize the AFM state relative to the applied field. We term such an event as double‐layer switching (DLS). In the three‐step switching case, the switching sequence of the two FM layers is completely changed. The thinner layer will switch three times in all three steps, and the thicker layer will only switch once in the second step. Accordingly, the hysteresis loop no longer exhibits as an overall shift as shown in the two‐step switching of the SFi. During SLS in three‐step switching, the effect of IL‐DMI on FM is consistent with that described in two‐step switching. However, the switching sequence changes of the two layers make the outer loops expand or contract instead of shifting. Here, the expansion (contraction) represents the change in the width of the outer loop, which occurs when the magnetization switching is both hindered (assisted) as *H_Z_* is ascending and descending. In the process of DLS, *H_IL‐DMI_* and *H_IP_* along the same direction will hinder its transition to the next state instead (see details in Section S3). Consequently, the three steps in the hysteresis loop will behave as expanding‐shifting‐contracting in sequence (Figure S5d). In the DLS process, the combination of asymmetric magnetization and the antisymmetric IL‐DMI leads to richer magnetization reversal dynamics. Due to the antiferromagnetic coupling between the upper and lower layers and their simultaneous switching needs, the system inevitably passes through a state where the two layers are both aligned in‐plane but antiparallel. At this moment, owing to the chiral nature of the IL‐DMI, the resulting effective field is no longer in‐plane but instead perpendicular that directly competes with the externally applied out‐of‐plane scanning field. As a result, the effective switching field shift caused by IL‐DMI is significantly amplified (Figure S6). Due to its different form of energy contribution in the Arrhenius law [29], the switching field shifting degree in the inner loop will significantly increase. Therefore, we can utilize this amplified hysteresis loop shift during DLS to measure IL‐DMI more efficiently.

To demonstrate experimentally the aforementioned two types of asymmetric switching, we deposit SFi films of Ta(30)/Pt(30)/Co(*t*)/Ir(14.5)/Co(10)/Cu(10)/Ta(30) (layer thicknesses in angstroms in parentheses) by magnetron sputtering. See Materials and Methods for growth details and the possible origin of IP symmetry breaking to generate IL‐DMI. In order to verify the two types of hysteresis loop behavior, we fabricate three kinds of samples S_1, S_2, and S_3 (the numbers indicate the thickness difference in angstroms between the upper FM and the lower FM layers) by changing the thickness *t* of the lower FM layer to 9, 8, and 7 Å, respectively. Accordingly, the hysteresis loop of S_1 behaves as a two‐step switching, and the hysteresis loops of S_2 and S_3 behave as three‐step switching. AP+ and AP‐ represent the antiparallel states with magnetization up (+) and down (‐) of the thicker upper Co layer, respectively. The basic magnetic properties and out‐of‐plane AHE curves of the three samples are presented in Section S4. Then the asymmetric AHE switching loops due to IL‐DMI and an additionally applied *H_IN_* are measured using the configurations shown in Figure 1b,c.

**FIGURE 2:**
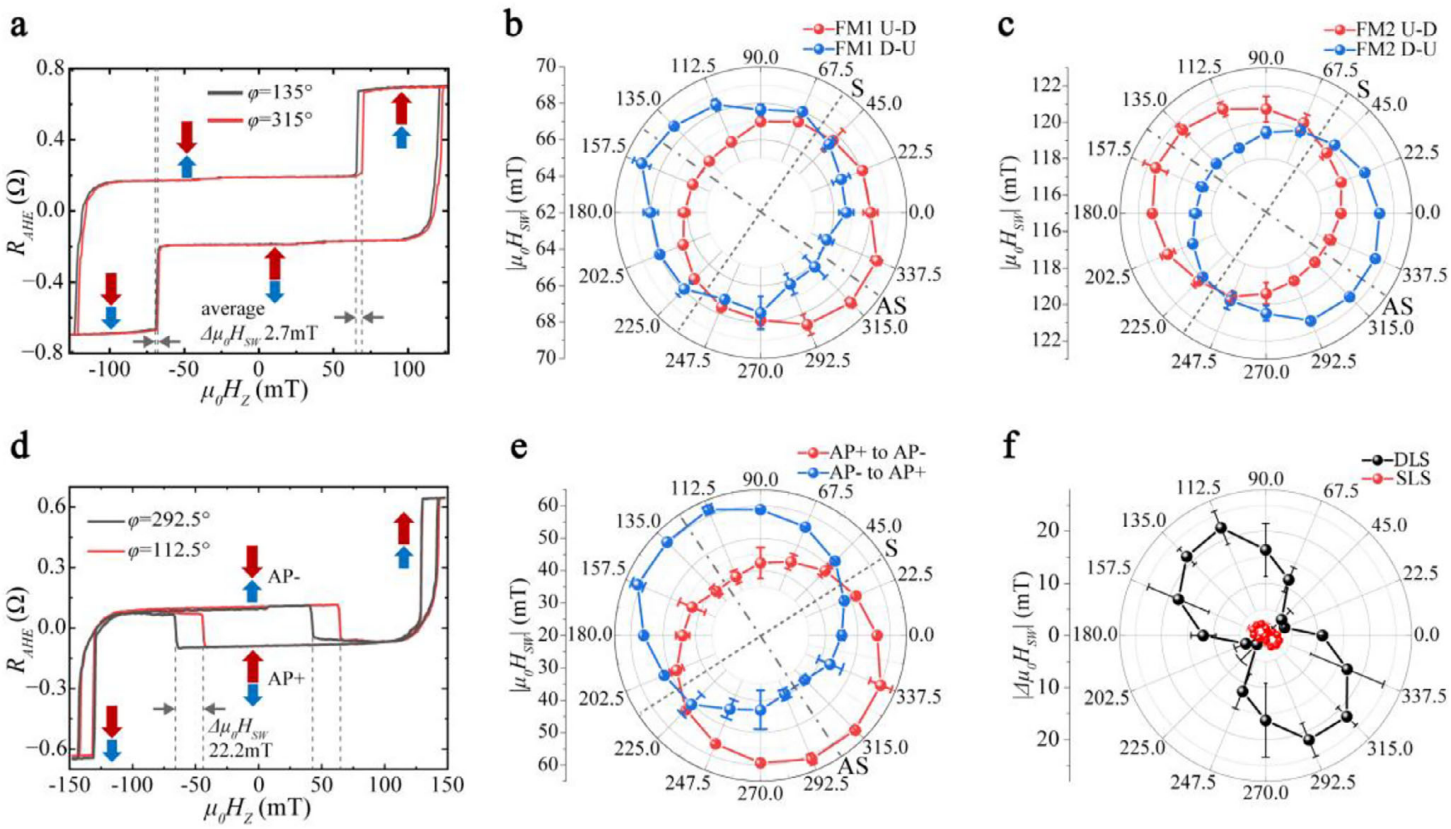
Characterization of the IL‐DMI effect for SFi in both two‐step switching and three‐step switching. (a) Maximum AHE loop shifts of SFi S_1 measured at azimuth angles of 135° and 315° in the case of two‐step switching. Angle *φ* refers to the additional *H_IN_* direction. (b and c) Azimuthal angular dependence of the switching field of the upper layer (b) and lower layer (c) of SFi S_1. The asymmetric (AS) axis and symmetric (S) axis are along 145° and 55°, respectively. (d) Maximum switching field shifts of SFi S_2 during the DLS process were measured at azimuth angles of 112.5° and 292.5° in the case of three‐step switching. (e) Azimuthal angular dependence of the switching field during the DLS process of S_2. AP+ and AP‐ represent the antiparallel states with magnetization up and down in the upper Co layer, respectively. The AS axis and S axis are along 123° and 33°, respectively. (f) Comparison of azimuthal angular dependence of the switching field difference *|△µ*_0_ *H_SW_|* between S_1 (SLS) and S_2 (DLS). The maximum switching field difference Δμ_0_ *H_SW_* is 2.7 (±0.3) and 22.2 (±3.4) mT, respectively.

We first measure the AHE loop of S_1 to verify the consistency of IL‐DMI behavior in the SFi with two‐step switching and the SAF with two‐step switching reported before [13]. As shown in Figure 2a, the AHE loops at the azimuth angle *φ* = 135° and *φ* = 315° are slightly shifted to the left and right, respectively. Angle *φ* refers to the additional *H_IN_* direction. The vertical dashed lines indicate the maximum AHE loop shift of SFi S_1 corresponding to the switching process shown in Figure 1d. The switching field difference Δμ_0_ *H_SW_* =  2.7 mT is obtained by averaging the two shifts for switching from parallel to antiparallel (shown by dashed lines and arrows). Theoretically, these two shifts should be equal. However, the shifts measured in actual experiments are generally different. The difference between the two shifts is inevitable, which is caused by an angle calibration error or a misaligned magnetic field. Figure 2b,c shows the azimuthal angular dependence of the switching field of the upper layer and lower layer of S_1 with the IP field, respectively. The magnitude of the IP field is kept as *H_IN_* = *H_ext_* *sin*θ as the direction of it rotates from 0 to 180°. We find that the magnetization switching of S_1 exhibits opposite unidirectional anisotropy in the two FM layers. This asymmetric distribution of the switching field confirms the presence of IL‐DMI in our sample, and the asymmetry (AS) axis is along *φ* = 145°, and the symmetry (S) axis is along *φ* = 55°. The switching field difference Δμ_0_ *H_SW_* is 2.8 (±0.7) and 2.7 (±0.5) mT for switching from antiparallel to parallel and from parallel to antiparallel, respectively.

With the basis of the experimental results of two‐step switching, we now verify the proposed asymmetric switching behavior for three‐step switching in an SFi. We measure the asymmetric AHE loops of sample S_2 to determine its IL‐DMI bias field. As expected, in the case of three‐step switching, the hysteresis loop behaves as the expanding‐shifting‐contracting under both *H_IL‐DMI_* and *H_IN_* along the AS axis in the *x‐y* plane. Figure 2d shows the maximum switching field shifts of SFi S_2 during the DLS process with *H_IN_* applied at azimuth angles of 112.5° and 292.5°. Near the AS axis, the inner loops for DLS are clearly shifted to the left and right, respectively (see comparison in Section S5). Figure 2e shows the azimuthal angular dependence of the switching field during the DLS process of S_2. The AS and S axes of IL‐DMI can be clearly distinguished. Figure 2f shows the comparison of the azimuthal angular dependence of the switching field difference *|△µ_0_H_SW_|* between SLS in S_1 and DLS in S_2. We found the maximum switching field difference Δμ_0_ *H_SW_* is 2.7 (±0.3) and 22.2 (±3.4) mT, respectively. The expansion or contraction behavior of the outer loops are shown in Section S6. Furthermore, we confirm the magnitude of the IL‐DMI field in S_2 to be 1.23 (±0.35) mT by calculating the switching field width difference of the outer loops in the SLS region, where the error bar represents the standard deviation. Theoretically, due to the expansion or contraction characteristics of the outer loops, we can calibrate the two hysteresis loops with the center of the outer loops as a reference to eliminate the shifting caused by angular errors or misaligned field. This procedure avoids the unequal situation of the shifts that appear in Figure 2a. The deposition conditions of S_2 and S_1 are exactly the same, differing only in the thickness of the lower FM. Indeed, the intensity of the IL‐DMI effective fields of the two (1.23 and 2.7 mT) is found to be close in magnitude. However, the loop shift of sample S_2 during DLS (22.2 mT) is approximately 20 times larger than the loop shift during SLS (1.23 mT). We believe that this amplification in shift magnitude is caused by the simultaneous switching of both the upper and lower layers in DLS. According to Arrhenius law [29], the direct competition between IL‐DMI and the perpendicular driving field will significantly increase the switching field shift (see details in Section S7). To eliminate the influence of varying IP field magnitudes on the switching field offset, we conduct additional asymmetric AHE measurements on samples S_1 and S_2. Instead of rotating the sample rod to obtain the AHE loops, we fixed the IP field magnitude and scanned the out‐of‐plane field. The results show that the switching field offsets for SLS and DLS are consistent in magnitude with the existing measurement results (see details in Section S8). With outer loops calibration and large‐shifting in the inner loops, we discovered a more effective IL‐DMI measurement in a three‐step switching process. This detection method is applicable to all synthetic magnetic systems and can be easily implemented by adjusting the thickness of one of the layers (see complete measurement results in Section S9).

### 2.2 Transient State Detection in SOT Switching

**FIGURE 3:**
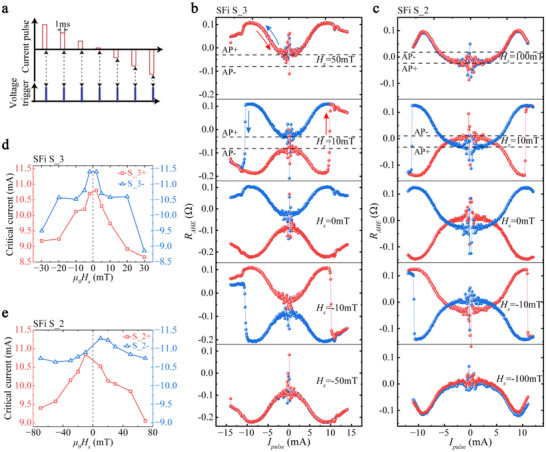
Detection of the transient resistance states of SFi S_3 and S_2 during the SOT switching. (a) Schematic illustration of the transient state detection method for SOT switching. The AHE voltage detection occurs at the moment the applied SOT pulse ends. The pulse width of SOT is 1 ms. (b,c) *R_AHE_* versus SOT pulse curves of S_3 (b) and S_2 (c) under different IP fields. Under the action of IL‐DMI, SOT switching is no longer a dual‐resistance state switching. The red and blue data represent the ascending and descending current scanning results of the SOT pulse, respectively. The horizontal dashed lines represent the resistance values of the two AP states of SFi. It should be noted that compared with S_3, the sign of the AP+ and AP‐ signal of S_2 is reversed due to the thicker bottom Co. No SOT switching occurs without *H_x_*. With proper *H_x_* assistance, deterministic SOT switching can be achieved. However, when *H_x_* exceeds a certain value (50 mT for S_3 and 100 mT for S_2), no SOT switching occurs, and the curves of positive and negative current sweeping coincide. Due to the influence of IL‐DMI, SOT current and thermal effects, the magnetization of the lower layer Co will gradually turn to IP with the increase of current. The upper and lower layers tend to align perpendicularly before switching. (d,e) Asymmetric critical current distribution with different *H_x_* of S_3 (d) and S_2 (e). The critical current in the positive scan is always smaller than that in the negative scan.

At present, one of the most promising applications of SFi materials is to utilize SOT to switch the SFi magnetic moment and apply it to data storage and logic computing [30]. For SOT switching, SFi has the advantages of high‐speed, minimized magnetic crosstalk, extra robustness against external field perturbation, etc., and its net magnetic moment allows for easy magnetic reading [31, 32]. However, there are currently only limited articles that have studied the SOT switching of SFi in the presence of IL‐DMI. The research results are mainly limited on IL‐DMI assisting SOT to achieve deterministic switching [15, 16]. To comprehensively study the impact of IL‐DMI on SOT switching, we apply two methods of detection: Method I, to detect the transient state right after the SOT switching pulse; Method II, to detect the steady state after the SOT pulse applying and a time interval. Figure 3a shows the schematic illustration of Method I. In this case, volatile and nonvolatile magnetization changes induced by SOT are simultaneously probed. Figure 3b,c shows the dependence of *R_AHE_* with *I_pulse_* in SFi S_3 and S_2 Hall bars (10‐µm wide) with the assistance of different IP fields *H_x_*. Under the joint action of IL‐DMI, spin current, and thermal effect, it is no longer the mutual binary switching of the two antiparallel states of AP+ and AP‐ [33]. The continuous rising or falling behavior of *R_AHE_* before switching (range from −10 to 10 mA) indicates that the upper and lower layers of SFi tend to be arranged perpendicularly with the current increasing, which is caused by the IP polarization current of SOT, the chiral configuration preferred by IL‐DMI, and thermal effects. We refer to this as analog‐like SOT switching behavior. In previous work [33, 34, 35], the same method is used to detect SOT switching of SAF stacks without IL‐DMI. To the best of our knowledge, none of these reported the analog‐like switching curves. Therefore, we believe that IL‐DMI plays a key role in this switching behavior. The red and blue lines represent the ascending (+) and descending (−) current scanning results of *I_pulse_*, respectively. First, no SOT switching occurs without *H_x_*. Second, with proper *H_x_* assistance, deterministic SOT switching can be achieved. When *H_x_* is reversed, the switching polarity of the SOT is also reversed. However, when *H_x_* exceeds a certain value (50 mT for S_3 and 100 mT for S_2), no SOT switching occurs, and the curves of positive and negative current sweeping coincide. We believe this is because when *H_x_* is large enough, SFi will spontaneously switch to only one of the antiparallel states so that the chiral exchange bias field of IL‐DMI is consistent with *H_x_*. In this case, no matter the current is scanning positively or negatively, SFi prefers only one of the AP states, and no deterministic switching will happen due to IL‐DMI. It is also verified by seeing that the SFi only prefers the AP+ or AP‐ states when *H_x_* reaches +50 or −50 mT, respectively. One previous article reported that perpendicular magnetization switching of SAF with IL‐DMI can be achieved using IP field only [36], which to some extent is consistent with our SOT results when *H_x_* is large enough. Further, we plot the dependence of the critical switching current of the device as a function of *H_x_*. As shown in Figure 3d,e, regardless of the direction of *H_x_*, the absolute value of the critical current in the positive scan (red square) is always smaller than that in the negative scan (blue triangle) at each *H_x_* value (see Section S10 for the procedure for extracting the threshold switching current and complete analog‐like SOT switching of S_3 and S_2). Similar results are also obtained in sample S_1, and the detailed switching results can be found in Section S11. SOT‐induced Kerr‐imaging dynamics and size‐scaling results of the Hall bar devices in Section S12 rule out the possibility that the analog‐like switching originates from multidomain or domain wall motion. In future work, we aim to adopt layer‐resolved techniques, such as layer‐resolved MOKE, XMCD‐PEEM or magnon‐mode spectroscopy [37] to further directly visualize and verify the layer‐specific trajectories. The critical switching current decreases with the increase of effective IP field. The reversal of *H_x_* will cause the polarity switching of the SOT. Accordingly, the chiral bias field due to IL‐DMI will be reversed in another AP state. Thus, the unidirectional asymmetric behavior of the critical current is due to the fact that *H_x_* and *H_IL‐DMI_* are always oriented in the same direction once the scanning direction is fixed (the micromagnetic simulation section that follows will provide further explanation and simulation validation). This result can in turn be used as a proof of the chiral exchange bias generated by IL‐DMI.

It is worth noting that the analog‐like SOT switching observed in our devices is different from the memristive SOT switching reported in previous studies. Memristive SOT switching refers to partial reversal in large‐sized devices caused by the impediment of domain wall motion due to changes in local exchange bias or other factors [38, 39, 40]. The memristive SOT switching in our device is also demonstrated (see details in Section S13).

### 2.3 Steady State Detection in SOT Switching

**FIGURE 4:**
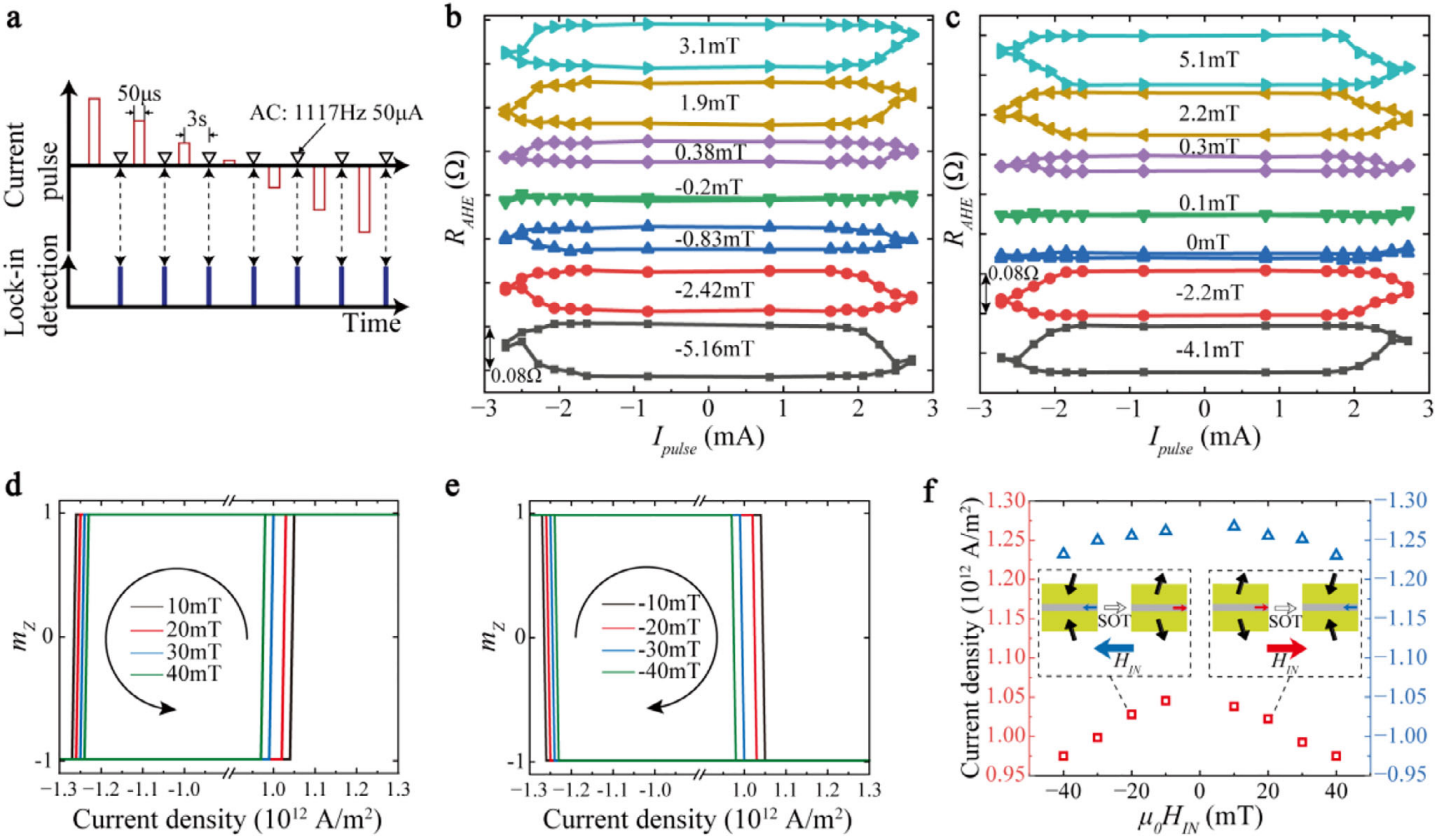
Detection of the steady resistance states of SFi S_2 during the SOT switching and micromagnetic simulation of asymmetric critical switching current distribution. (a) Schematic illustration of the steady state detection method for SOT switching. AHE detection occurs 3 s after SOT pulse application, achieved with a small AC current and lock‐in technology. The pulse width of SOT is 50 µs. The amplitude of AC current is 50 µA, and the frequency is 1117 Hz. (b and c) *R_AHE_* versus SOT pulse curves of S_2 with Hall bar angle at *φ* = 0° (b) and *φ* = 90° (c) under different IP fields. The effective fields produced by IL‐DMI at *φ* = 0° and *φ* = 90° are 0.2 and −0.1mT, respectively, as shown by the green curves. (d,e) Micromagnetic simulation of SFi Magnetization switching versus SOT current density with the assistance of positive (d) and negative (e) IP fields. (f) Asymmetric critical current distribution with different IP fields obtained from micromagnetic simulation. Regardless of the direction of *H_IN_*, the critical current in the positive scan (red square) is always smaller than that in the negative scan (blue triangle). The inset explains that the reason for this asymmetry is because *H_IN_* and *H_IL‐DMI_* always switch simultaneously.

After the transient resistance states detection, we also use Method II to detect the steady state to complete the research of the behavior of IL‐DMI in SOT switching. Figure 4a shows the schematic illustration of Method II. In order to eliminate the influence of thermal effects on the switching, after each SOT pulse, an interval of 3 s is waited, until we apply a small AC current to detect *R_AHE_* of SFi using the lock‐in technique. In this case, only the nonvolatile magnetization change induced by SOT is probed. Correspondingly, at this time, the SOT switching curve turned into the conventional digital‐like binary state switching, and the phenomenon of transient analog‐like state no longer appeared. We use this method to detect the effective field generated by IL‐DMI and verify whether it can assist SOT to achieve field‐free switching. Here, we conduct experiments on symmetric Hall bars, where both stripes have the same width of 2 µm so that we can obtain the results with the IP field along both *φ* = 0° and *φ* = 90° in one device. Figure 4b,c shows the SOT switching curve of S_2 with the Hall bar (2‐µm wide) angle at *φ* = 0° and *φ* = 90° under different IP fields. In the case of *φ* = 0° (*φ* = 90°), almost no switching signal is detected in the sample when the IP field is −0.2 mT (0.1 mT), and the SOT curves show the opposite switching polarity before and after this field. These results indicate that the effective fields produced by IL‐DMI at *φ* = 0° and *φ* = 90° are 0.2 and −0.1 mT, respectively. We further demonstrate this effective field by plotting the AHE resistance difference before and after switching as a function of magnetic field (Section S14). The above results show that the effective field for assisting SOT changes the direction at 0° and 90°, which is consistent with the effective field direction of the IL‐DMI. However, the magnitude of the effective field we measured is relatively small compared to the *H_IL‐DMI_* measured in asymmetric hysteresis loop switching. For this part, see Section S15 for the reason behind.

### 2.4 Micromagnetic Simulations for SOT Switching

In order to explain these phenomena caused by the combination of IL‐DMI and SOT, we proceed to use micromagnetic simulation to verify the present experimental results (see details in Section S16). Since the field‐free SOT switching has been demonstrated in previous articles [15, 16, 41, 42, 43], we focus on the asymmetric critical current distribution and the analog‐like switching behavior. Figure 4d,e shows the magnetization switching of SFi with SOT current with the assistance of positive and negative fields, respectively. As the direction of the IP field reverses, the SOT switching polarity also reverses, as shown by the rotation direction of the arrows in Figure 4d,e. However, the critical current in the positive scan is always smaller than that in the negative scan. Figure 4f shows the simulation result of unidirectional asymmetric critical current distribution, which is consistent with the experimental results shown in Figure 3d,e. The critical current density for SOT switching decreases as the IP assist field increases. The inset explains that this unidirectional asymmetric behavior is due to the SOT polarity switching as *H_IN_* reverses. Correspondingly, the effective IL‐DMI field switches, too. Therefore, during negative current sweeping, *H_IN_* and *H_IL‐DMI_* are always in the same direction and adding up. During the positive current sweeping, *H_IN_* and *H_IL‐DMI_* are always in the opposite direction and subtracting. Overall, the critical current in a negative scan will always be smaller than that in positive scan.

We further investigate the dynamics of magnetization during the SOT switching process of the SFi. We demonstrate that under the influence of the spin current effect and IL‐DMI (without considering thermal effects here), the magnetizations of the top and bottom layers gradually transition from an antiparallel alignment to a perpendicular alignment during the switching process due to the IL‐DMI effect (Section S16). This confirms the possibility that IL‐DMI contributes to this type of analog‐like SOT switching, as IL‐DMI causes the top layer's magnetization to gradually rotate to IP and thereby enhances the overall AHE signal of the SFi compared to the antiparallel arrangement.

### 2.5 Spintronic LIF Neuron Implementation in SNN

Neuromorphic computing, a computational paradigm inspired by the human brain, has gained widespread application and is increasingly attracting the attention of researchers [44]. SNNs, with their unique sparse, asynchronous, and event‐driven computing, closely mimic the biological processes of the human brain, making them highly suitable toward energy‐efficient neuromorphic systems [45]. Spintronics holds great potential to provide hardware support for neuromorphic computing [46, 47, 48] due to its nonlinear characteristics, hysteresis, collective behavior, and non‐volatility. The synergies between SNNs and spintronic SOT devices with fast speed, energy efficiency, and non‐volatile properties hold the promise of creating neuromorphic systems that are capable of performing complex tasks with high precision and low energy consumption. However, due to the digital‐like (binary‐state) SOT switching characteristic, LIF neuron models based on it are always probabilistic. That is, the neurons in this model fire stochastically, and the probability of firing at a particular time is a nonlinear function of the instantaneous magnitude of the weighted input [39, 45]. A network with stochastic neurons is evaluated over multiple iterations, and the output of a layer is computed as the average number of spikes over all iterations [45]. The variability introduced by stochasticity can prevent the model from overfitting, leading to better generalization and noise resistance. In contrast, the deterministic LIF model eliminates the need for multiple iterations and has been demonstrated to achieve higher accuracy and sparsity. More importantly, the deterministic LIF model exhibits superior energy efficiency [49]. The analog SOT behavior found in our IL‐DMI devices naturally exhibits the LIF characteristics of a neuron: the device resistance increases progressively with current density (integrate), resets to a low‐resistance antiparallel state once the current is removed (leaky), and undergoes switching when a threshold is exceeded (fire). Therefore, we leverage this to achieve the first SOT‐based deterministic LIF neuron device and integrate it into an SNN for handwritten digit recognition.

**FIGURE 5:**
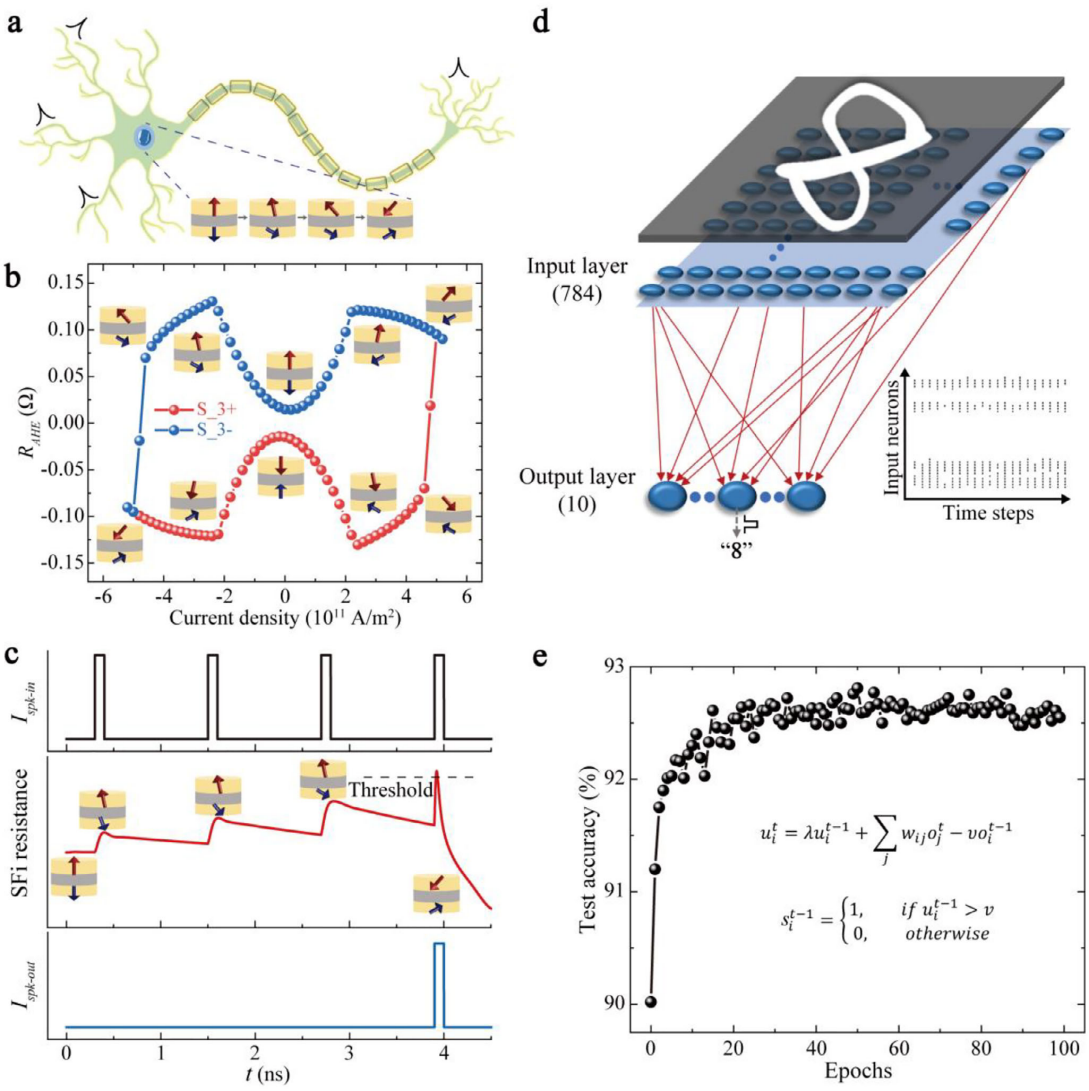
SNN with LIF neurons based on analog‐like SOT switching behavior. (a) Schematic of an LIF neuron achieved by an SFi device with different magnetization configurations induced by SOT. (b) Micromagnetic simulation results of the AHE resistance of S_3 as a function of current density. The insets indicate the magnetization configurations excited by different current densities. (c) Demonstration of the LIF behavior of an artificial neuron based on analog‐like SOT switching by micromagnetic simulation. (d) The topological structure of the proposed SNN. The numbers in parentheses indicate the number of neurons. (e) MNIST digital handwritten patterns recognition accuracy as a function of learning epochs.

Figure 5a shows the schematic of a LIF neuron achieved based on analog‐like SOT switching. Here, micromagnetic simulations are first used to reproduce the analog‐like SOT switching behavior. Figure 5b shows the micromagnetic simulation results of *R_AHE_* of S_3 as a function of current density. It shows good agreement with the experimental results in Figure 3b (*H_x_* = 10 mT) after including the thermal effect (Sections S17 and S18). We first consider the temperature rise in the device induced by the SOT current. Then, using the saturation magnetization *M_S_* (0.55 MA/m) and perpendicular magnetic anisotropy (0.52 MJ/m^3^) extracted from experimental data at 300 K, along with their temperature‐dependent models, we dynamically incorporate the temperature‐induced variations of *M_S_* and *K* into the MuMax3 simulations. The switching behavior closely matches the LIF characteristics of neurons. The initial state of the SFi is one of the AP states. With the application of current pulses, the magnetic moments of the upper and lower layers of the SFi gradually tilt, and the resistance begins to increase as the current integrates. However, these states are volatile. When the current is removed, the SFi gradually leaks back to the initial AP state. Finally, when the current integrates to a certain level, the SFi undergoes switching, corresponding to the firing characteristic of neurons. Figure 5c shows the demonstration of the LIF behavior based on analog‐like SOT switching. A current of appropriate magnitude (5 × 10^11^ A/m^2^) and fixed duration (0.1 ns) is applied.

Figure 5d shows the topological structure of the proposed SNN. The topology of a typical two‐layer SNN was adopted with inputs consisting of a 2D array of 28 × 28 pixels. The original input pixels are first converted into spike trains using Poisson encoders (inset of Figure 5d), and these spike trains are then sequentially fed into the network. The classification output is determined by the output neuron that generates the most frequent spikes. The synaptic weights connecting the input and output neurons are trained using the surrogate gradient method, one of the most efficient algorithms for training spiking neural networks [46]. We integrate the analog‐like SOT switching into the differential equations of neurons and model the LIF dynamics. The formula in Figure 5e is the discrete and recurrent representation of the LIF neuron, where *u* is the output of the neuron, *t* represents the timestep, the subscripts *i* and *j* denote the post‐ and pre‐neurons, respectively, λ is the leaky constant, ω is the synaptic weight, *s* represents the output spike, and ν is the firing threshold. To perform the real‐world task using the IL‐DMI‐assisted SOT switching behavior, we have extracted hyperparameters such as the leaky constant λ from the characteristics of the device. This SOT‐based neuron model is then integrated into the SNN for training. As shown in Figure 5e, the accuracy of MNIST handwritten digit recognition increases with learning epochs, achieving a testing accuracy of 92.5% after 25 epochs (see Sections S19 and S20 for the training details of the network). The energy consumption per spike of our neuron, constructed using analog‐like SOT switching, is 2–4 orders of magnitude lower than that of current neuromorphic manycore processors (see Section S21 for the calculation details). The analog‐like SOT switching offers a promising direction for SOT‐based neuromorphic computing applications. With the combination of memristive behavior achieved through multistate SOT switching [38, 40] (Figure S24) to mimic the synapses, it becomes feasible to realize a fully spintronic neural network based on SOT.

## 3 Conclusion

In conclusion, we unravel the role of IL‐DMI in both field‐induced and SOT‐induced magnetization switching of SFi structures and pioneer its application in neuromorphic computing. We report the asymmetric behaviors in the hysteresis loops caused by IL‐DMI, both with and without the DLS process. With DLS, the inner asymmetric loop shift is amplified approximately 20‐fold, and the outer loops enable calibration to eliminate field misaligning errors. Both advantages will greatly enhance the future detection of IL‐DMI. This approach is universal and can be easily implemented by varying the thickness of one of the FM layers. Using transient‐state and steady‐state resistance measurements, we explore IL‐DMI's impact on SFi's SOT switching. Beyond producing an asymmetric unidirectional critical current distribution, IL‐DMI can also assist SOT to realize an analog‐like switching behavior. Accordingly, we integrate the analog‐like SOT switching into an SNN as a deterministic LIF neuron and achieve the handwritten digit recognition on the MNIST dataset. This work reveals the role of IL‐DMI in SFi structures and extends its potential applications in ultralow‐power, high‐sparsity and high‐accuracy neuromorphic computing.

## 4 Materials and Methods

### 4.1 Sample and Device Fabrication

In the experiment, a sample series of Ta(30)/Pt(30)/Co(*t*)/Ir(14.5)/Co(10)/Cu(10)/Ta(30) (layer thicknesses in angstroms) stacks are deposited on thermally oxidized silicon substrates by DC sputtering under a base pressure lower than 10^−8^ Torr. Cu is used to weaken the AHE of the top Co layer, resulting in a larger AHE signal difference in our stacks [50]. We do not impose a special way of IP symmetry breaking on purpose. However, we speculate that the IP symmetry breaking is caused by the oblique sputtering of Ir targets. One of the evidence is that the asymmetric axes of the IL‐DMI in all our devices are along the second and fourth quadrants (see Section S9). In addition, some articles reported that IL‐DMI can still be detected even in a normally sputtered Co/Ir/Co control sample [36]. A superconducting quantum interference device (SQUID) was used to quantify the magnetic moments of each sample. The films are fabricated into Hall bar devices of 20, 10, 4, and 2 µm width by optical lithography and lift‐off techniques. All the electrodes are then attached to 45 nm Ti/Au to enable the contact during the electrical measurements, using e‐beam evaporation.

### 4.2 Electrical Measurements

The measurement of IL‐DMI is achieved through a magneto‐resistance setup. We apply a constant magnetic field first. By rotating the sample clockwise or counterclockwise, we can get the AHE hysteresis loop while applying a constant‐direction IP field to the sample. Similarly, we apply a constant in‐plane field and sweep the perpendicular field to measure the AHE loop of the device, and perform comparative measurements. The transient state and steady state detections in SOT switching measurement are achieved through Keithley 6221, 2182, and Keithley 33250, 6221, Stanford SR830, respectively.

### 4.3 Micromagnetic Simulation

Micromagnetic simulations are carried out by solving numerically the LLG equation augmented with a damping‐like SOT that considers the contribution of the current flowing through the heavy metal layer. We include the contribution of the IL‐DMI as an effective field in the micromagnetic simulator Mumax3 with the energy form as *E_DM_* =  *D* · (*S*_1_ × *S*_2_). The IL‐DMI and RKKY coupling strength are set as *D_1,2_* = 0.12 mJ/m^2^ and *J_RKKY_* = 0.3 mJ/m^2^, respectively. The spin polarization is set as [0,1,0] when the current is along [1,0,0].

### 4.4 SNN Training

A discrete, recurrent representation of the LIF neuron from the ordinary differential equation (ODE) describing the RC circuit can be derived as: $\tau_{m}\;\frac{du}{dt}=-u+R_{m}I\left(t\right),$ where *u* is the membrane potential, *I*(*t*) is the input current, and τ_*m*_ = *R_m_* *C_m_* is the time constant of the circuit. The forward Euler method is used to solve the above linear ODE, providing a good enough approximation of continuous‐time integration. This gives a good enough approximation of continuous‐time integration. By isolating the membrane potential at the following time step, the equation becomes: $u\;\left(t+\Delta t\right)=\left(1-\frac{\Delta t}{\tau_{m}}\right)\;u\left(t\right)+I\;\left(t\right)=\lambda u\left(t\right)+wI\left(t\right).$ The neuron emits an output spike if the membrane exceeds the threshold *v*. For a specific neuron node in a SNN, the weighted sum of the input current is taken as overall inputs: $u_{i}\;\left(t+\Delta t\right)=\lambda u_{i}\left(t\right)+\sum_{j}w_{ij}I\left(t\right)-vS\left(t\right),$ where *w_ij_* is learnable parameter, and *v* is often set to 1 (can be tuned), this leaves the λ  as the primary hyperparameter to be specified. The parameter λ (set to 0.28) is extracted from the characteristics of the IL‐DMI device and integrated into the model for training using the surrogate gradient method. Specifically, we use the spikingjelly pakage for the SNN training on the MNIST dataset, which contains 60 000 training images and 10 000 testing images. the simulating time step T was set to 100, the batch size was set as 64, and the Adaptive moment estimation (ADAM) optimier was used for the training.

## Author Contributions

S.L., T.H., S.M., and X.L. conceived the original idea. S.L., T.H., X.L., W.Z., and R.L. planned and designed the experiments. X.C. performed the SNN simulations. T.H. and C.L. fabricated the samples. S.L. performed IL‐DMI measurements and steady SOT measurements. T.H. and S.L. performed transient SOT measurements. M.F., S.L., F.G.‐S., and E.M. performed micromagnetic simulations. All the authors participated in discussions of the research.

## Funding

This work was supported in part by the National Natural Science Foundation of China (Nos. 62371019, T2394475, 92164206, and 52261145694), the Beijing Natural Science Foundation (No.4232070), the Research Start‐Up Funds of Hangzhou International Innovation Institute of Beihang University Under Grant (Nos. 2024KQ052 and 2025BKZ005), the International Mobility Project (No. B16001), the China Scholarship Council (CSC) and the European Union's Horizon 2020 research and innovation program under the Marie Sklodowska‐Curie grant agreement (No. 860060).

## Conflicts of Interest

The authors declare no conflicts of interest.

## Supporting information




**Supporting File**: advs73620‐sup‐0001‐SuppMat.docx.

## Data Availability

The data that support the findings of this study are available from the corresponding author upon reasonable request.

## References

[1] K. Wang , V. Bheemarasetty , and G. Xiao , “Spin Textures in Synthetic Antiferromagnets: Challenges, Opportunities, and Future Directions,” APL Materials 11 (2023): 070902, 10.1063/5.0153349.

[2] R. A. Duine , K.‐J. Lee , S. S. P. Parkin , and M. D. Stiles , “Synthetic Antiferromagnetic Spintronics,” Nature Physics 14 (2018): 217–219, 10.1038/s41567-018-0050-y. 29910827 PMC5997292

[3] Y.‐C. Lau , D. Betto , K. Rode , J. M. D. Coey , and P. Stamenov , “Spin–orbit torque switching Without an External Field Using Interlayer Exchange Coupling,” Nature Nanotechnology 11 (2016): 758–762, 10.1038/nnano.2016.84. 27240416

[4] S.‐H. Yang , K.‐S. Ryu , and S. S. P. Parkin , “Domain‐Wall Velocities of up to 750 m s−1 driven by Exchange‐Coupling Torque in Synthetic Antiferromagnets,” Nature Nanotechnology 10 (2015): 221–226, 10.1038/nnano.2014.324. 25705867

[5] D. Houssameddine , J. F. Sierra , D. Gusakova , et al., “Spin Torque Driven Excitations in a Synthetic Antiferromagnet,” Applied Physics Letters 96 (2010): 072511, 10.1063/1.3314282.

[6] Z. Yu , M. Shen , Z. Zeng , et al., “Voltage‐controlled Skyrmion‐based Nanodevices for Neuromorphic Computing Using a Synthetic Antiferromagnet,” Nanoscale Advances 2 (2020): 1309–1317, 10.1039/D0NA00009D. 36133072 PMC9419653

[7] M. A. Ruderman and C. Kittel , “Indirect Exchange Coupling of Nuclear Magnetic Moments by Conduction Electrons,” Physical Review 96 (1954): 99–102, 10.1103/PhysRev.96.99.

[8] K. Yosida , “Magnetic Properties of Cu‐Mn Alloys,” Physical Review 106 (1957): 893–898, 10.1103/PhysRev.106.893.

[9] T. Kasuya , “A Theory of Metallic Ferro‐ and Antiferromagnetism on Zener's Model,” Prog Theor Phys 16 (1956): 45–57.

[10] S. Parkin , Xin Jiang , C. Kaiser , A. Panchula , K. Roche , and M. Samant , “Magnetically Engineered Spintronic Sensors and Memory,” Proceedings of the IEEE 91 (2003): 661–680, 10.1109/JPROC.2003.811807.

[11] M. Wang , W. Cai , D. Zhu , et al., “Field‐Free Switching of a Perpendicular Magnetic Tunnel Junction Through The Interplay of Spin–Orbit and Spin‐Transfer Torques,” Nature Electronics 1 (2018): 582–588, 10.1038/s41928-018-0160-7.

[12] E. Y. Vedmedenko , P. Riego , J. A. Arregi , and A. Berger , “Interlayer Dzyaloshinskii‐Moriya Interactions,” Physical Review Letters 122 (2019): 257202, 10.1103/PhysRevLett.122.257202. 31347891

[13] D.‐S. Han , K. Lee , J.‐P. Hanke , et al., “Long‐range Chiral Exchange Interaction in Synthetic Antiferromagnets,” Nature Materials 18 (2019): 703–708, 10.1038/s41563-019-0370-z. 31160801

[14] A. Fernández‐Pacheco , E. Vedmedenko , F. Ummelen , R. Mansell , D. Petit , and R. P. Cowburn , “Symmetry‐Breaking Interlayer Dzyaloshinskii–Moriya Interactions In Synthetic Antiferromagnets,” Nature Materials 18 (2019): 679–684, 10.1038/s41563-019-0386-4. 31160802

[15] Z. Wang , P. Li , M. Fattouhi , et al., “Field‐free Spin‐orbit Torque Switching of Synthetic Antiferromagnet Through Interlayer Dzyaloshinskii‐Moriya Interactions,” Cell Reports Physical Science 4 (2023): 101334, 10.1016/j.xcrp.2023.101334.

[16] W. He , C. Wan , C. Zheng , et al., “Field‐Free Spin–Orbit Torque Switching Enabled by the Interlayer Dzyaloshinskii–Moriya Interaction,” Nano Letters 22 (2022): 6857–6865, 10.1021/acs.nanolett.1c04786. 35849087

[17] N. Kent , N. Reynolds , D. Raftrey , et al., “Creation and Observation of Hopfions in Magnetic Multilayer Systems,” Nature Communications 12 (2021): 1562, 10.1038/s41467-021-21846-5. PMC7946913 33692363

[18] R. Lavrijsen , J.‐H. Lee , A. Fernández‐Pacheco , D. C. M. C. Petit , R. Mansell , and R. P. Cowburn , “Magnetic Ratchet for Three‐dimensional Spintronic Memory and Logic,” Nature 493 (2013): 647–650, 10.1038/nature11733. 23364743

[19] M. A. Cascales Sandoval , A. Hierro‐Rodríguez , S. Ruiz‐Gómez , et al., “Observation and Formation Mechanism of 360° Domain Wall Rings in Synthetic Anti‐ferromagnets With Interlayer Chiral Interactions,” Applied Physics Letters 123 (2023): 172407.

[20] M. A. C. Sandoval , A. Hierro‐Rodríguez , S. Ruiz‐Gómez , et al., “Preservation of Scalar Spin Chirality Across a Metallic Spacer in Synthetic Antiferromagnets with Chiral Interlayer Interactions,” arXiv (2024): 240407637.

[21] J. Zhou , L. Huang , H. J. Chung , et al., “Chiral Interlayer Exchange Coupling for Asymmetric Domain Wall Propagation in Field‐Free Magnetization Switching,” ACS Nano 17 (2023): 9049–9058, 10.1021/acsnano.2c11875. 37171183

[22] J. Yun , B. Cui , Q. Cui , et al., “Anisotropic Interlayer Dzyaloshinskii–Moriya Interaction in Synthetic Ferromagnetic/Antiferromagnetic Sandwiches,” Advanced Functional Materials 33 (2023): 2301731, 10.1002/adfm.202301731.

[23] J. Grollier , D. Querlioz , K. Y. Camsari , K. Everschor‐Sitte , S. Fukami , and M. D. Stiles , “Neuromorphic Spintronics,” Nature Electronics 3 (2020): 360–370, 10.1038/s41928-019-0360-9. PMC7754689 33367204

[24] K. Wang , L. Qian , S.‐C. Ying , and G. Xiao , “Spin‐orbit Torque Switching of Chiral Magnetization Across a Synthetic Antiferromagnet,” Communications Physics 4 (2021): 10, 10.1038/s42005-020-00513-z.

[25] F. Kammerbauer , W.‐Y. Choi , F. Freimuth , et al., “Controlling the Interlayer Dzyaloshinskii–Moriya Interaction by Electrical Currents,” Nano Letters 23 (2023): 7070–7075, 10.1021/acs.nanolett.3c01709. 37466639

[26] F. S. Gao , S. Q. Liu , R. Zhang , et al., “Experimental Evidence of the Oscillation Behavior of the Interlayer DMI Effect,” Applied Physics Letters 123 (2023): 192401, 10.1063/5.0177502.

[27] S. Liang , R. Chen , Q. Cui , et al., “Ruderman–Kittel–Kasuya–Yosida‐Type Interlayer Dzyaloshinskii–Moriya Interaction in Synthetic Magnets,” Nano Letters 23 (2023): 8690–8696, 10.1021/acs.nanolett.3c02607. 37695701

[28] M. Tanase , A. K. Petford‐Long , O. Heinonen , K. S. Buchanan , J. Sort , and J. Nogués , “Magnetization Reversal in Circularly Exchange‐biased Ferromagnetic Disks,” Physical Review B 79 (2009): 014436, 10.1103/PhysRevB.79.014436.

[29] S. Krause , G. Herzog , T. Stapelfeldt , et al., “Magnetization Reversal of Nanoscale Islands: How Size and Shape Affect the Arrhenius Prefactor,” Physical Review Letters 103 (2009): 127202, 10.1103/PhysRevLett.103.127202. 19792456

[30] Y. Zhang , X. Feng , Z. Zheng , et al., “Ferrimagnets for Spintronic Devices: From Materials to Applications,” Applied Physics Reviews 10 (2023): 011301, 10.1063/5.0104618.

[31] Z. Zheng , Y. Zhang , V. Lopez‐Dominguez , et al., “Field‐Free Spin‐Orbit Torque‐Induced Switching of Perpendicular Magnetization in a Ferrimagnetic Layer With a Vertical Composition Gradient,” Nature Communications 12 (2021): 4555, 10.1038/s41467-021-24854-7. PMC8316453 34315883

[32] Q. Shao , P. Li , L. Liu , et al., “Roadmap of Spin–Orbit Torques,” IEEE Transactions on Magnetics 57 (2021): 1–39, 10.1109/TMAG.2021.3078583. PMC10091395 37057056

[33] R. Chen , Q. Cui , L. Liao , et al., “Reducing Dzyaloshinskii‐Moriya Interaction and Field‐free Spin‐orbit Torque Switching in Synthetic Antiferromagnets,” Nature Communications 12 (2021): 3113, 10.1038/s41467-021-23414-3. PMC8149869 34035269

[34] J. Wei , X. Wang , B. Cui , et al., “Field‐Free Spin–Orbit Torque Switching in Perpendicularly Magnetized Synthetic Antiferromagnets,” Advanced Functional Materials 32 (2022): 2109455, 10.1002/adfm.202109455.

[35] R. Q. Zhang , G. Y. Shi , J. Su , et al., “Tunable spin–orbit torque switching in antiferromagnetically coupled CoFeB/Ta/CoFeB,” Applied Physics Letters 117 (2020): 212403, 10.1063/5.0031415.

[36] H. Masuda , T. Seki , Y. Yamane , et al., “Large Antisymmetric Interlayer Exchange Coupling Enabling Perpendicular Magnetization Switching by an in‐Plane Magnetic Field,” Physical Review Applied 17 (2022): 054036, 10.1103/PhysRevApplied.17.054036.

[37] Y. Shiota , T. Taniguchi , D. Hayashi , et al., “Handedness Manipulation of Propagating Antiferromagnetic Magnons,” Nature Communications 15 (2024): 9750, 10.1038/s41467-024-54125-0. PMC11579503 39567512

[38] S. Fukami , C. Zhang , S. DuttaGupta , A. Kurenkov , and H. Ohno , “Magnetization switching by spin–orbit torque in an antiferromagnet–ferromagnet bilayer system,” Nature Materials 15 (2016): 535–541, 10.1038/nmat4566. 26878314

[39] A. Kurenkov , S. DuttaGupta , C. Zhang , S. Fukami , Y. Horio , and H. Ohno , “Artificial Neuron and Synapse Realized in an Antiferromagnet/Ferromagnet Heterostructure Using Dynamics of Spin–Orbit Torque Switching,” Advanced Materials 31 (2019): 1900636, 10.1002/adma.201900636. 30989740

[40] J. Liu , T. Xu , H. Feng , et al., “Compensated Ferrimagnet Based Artificial Synapse and Neuron for Ultrafast Neuromorphic Computing,” Advanced Functional Materials 32 (2022): 2107870, 10.1002/adfm.202107870.

[41] Y.‐H. Huang , C.‐C. Huang , W.‐B. Liao , T.‐Y. Chen , and C.‐F. Pai , “Growth‐Dependent Interlayer Chiral Exchange and Field‐Free Switching,” Physical Review Applied 18 (2022): 034046, 10.1103/PhysRevApplied.18.034046.

[42] Y.‐C. Li , Y.‐H. Huang , C.‐C. Huang , Y.‐T. Liu , and C.‐F. Pai , “Field‐Free Switching in Symmetry‐Breaking Multilayers: The Critical Role of Interlayer Chiral Exchange,” Physical Review Applied 20 (2023): 024032, 10.1103/PhysRevApplied.20.024032.

[43] C.‐Y. Lin , P.‐C. Wang , Y.‐H. Huang , et al., “Field‐Free Spin–Orbit Torque Switching via Oscillatory Interlayer Dzyaloshinskii–Moriya Interaction for Advanced Memory Applications,” ACS Materials Letters 6 (2024): 400–408, 10.1021/acsmaterialslett.3c01376.

[44] D. Ham , H. Park , S. Hwang , and K. Kim , “Neuromorphic Electronics Based on Copying and Pasting the Brain,” Nature Electronics 4 (2021): 635–644, 10.1038/s41928-021-00646-1.

[45] N. Rathi , I. Chakraborty , A. Kosta , et al., “Exploring Neuromorphic Computing Based on Spiking Neural Networks: Algorithms to Hardware,” ACM Computing Surveys 55 (2023): 1–49, 10.1145/3571155.

[46] E. O. Neftci , H. Mostafa , and F. Zenke , “Surrogate Gradient Learning in Spiking Neural Networks: Bringing the Power of Gradient‐Based Optimization to Spiking Neural Networks,” IEEE Signal Processing Magazine 36 (2019): 51–63, 10.1109/MSP.2019.2931595.

[47] J. Torrejon , M. Riou , F. A. Araujo , et al., “Neuromorphic Computing With Nanoscale Spintronic Oscillators,” Nature 547 (2017): 428–431, 10.1038/nature23011. 28748930 PMC5575904

[48] O. Lee , T. Wei , K. D. Stenning , et al., “Task‐adaptive Physical Reservoir Computing,” Nature Materials 23 (2024): 79–87, 10.1038/s41563-023-01698-8. 37957266 PMC10769874

[49] Stochastic Spiking Neural Networks With First‐to‐Spike Coding , 2024 International Conference on Neuromorphic Systems (ICONS), (IEEE, 2024), 24–31.

[50] X. Fan , H. Celik , J. Wu , et al., “Quantifying Interface and Bulk Contributions to Spin–Orbit Torque in Magnetic Bilayers,” Nature Communications 5 (2014): 3042, 10.1038/ncomms4042. 24401766

