# Supplementary material for: Interlayer Dzyaloshinskii–Moriya Interaction in Synthetic Ferrimagnets for Spiking Neural Networks
Source: Adv Sci (Weinh). 2026 Jan 4;13(14):e19110. doi: 10.1002/advs.202519110 (PMC12970263; doi:10.1002/advs.202519110)
Supplement: Supplementary file 1 — Supporting File: advs73620‐sup‐0001‐SuppMat.docx. [file ADVS-13-e19110-s001.docx]

**Supplementary Information**

Interlayer Dzyaloshinskii-Moriya interaction in synthetic ferrimagnets for spiking neural networks

*Shen Li, Xing Chen, Mouad Fattouhi, Tianxun Huang, Chen Lv, Mark C. H. de Jong, Pingzhi Li**, Daoqian Zhu, Xiaoyang Lin^*^, Felipe Garcia-Sanchez, Eduardo Martinez, Stéphane Mangin, Bert Koopmans, Weisheng Zhao^*^ and Reinoud Lavrijsen*

S. Li, X. Lin, W, Zhao

State Key Laboratory of Spintronics, Hangzhou International Innovation Institute, Beihang University, Hangzhou 311115, China

E-mail: XYLin@buaa.edu.cn; weisheng.zhao@buaa.edu.cn

S. Li, M.C.H. Jong, P. Li, B. Koopmans, R. Lavrijsen

Department of Applied Physics, Eindhoven University of Technology, P.O. Box 513, 5600 MB Eindhoven, The Netherlands

S. Li, C. Lv, D. Zhu, X. Lin, W, Zhao

Fert Beijing Institute, MIIT Key Laboratory of Spintronics, School of Integrated Circuit Science and Engineering, Beihang University, Beijing 100191, China

X. Chen

Laboratoire Albert Fert, CNRS, Thales, Université Paris-Saclay, Palaiseau 91767, France

M. Fattouhi, F. Garcia-Sanchez, E. Martinez

Department of Applied Physics, Universidad de Salamanca, Plaza de la Merced, Salamanca 37008, Spain

T. Huang, C. Lv, S. Mangin

Institut Jean Lamour, UMR CNRS 7198, Université de Lorraine, Nancy 54011, France

**Table of Contents**

**Section S1.** The detailed procedure of asymmetric magnetization switching measurements.

**Section S2.** The detailed switching process analysis and simulation of SFi in two step-switching.

**Section S3**. The detailed switching process analysis and simulation of SFi in three-step switching.

**Section S4**. Basic film, magnetic and electrical transport properties of SFi S_1 to S_3.

**Section S5**. Comparison between the minimum and maximum switching field shifts of SFi S_2 during the DLS process.

**Section S6**. Characterization of the IL-DMI effect for SFi with three-step switching by measuring the switching field width during SLS process.

**Section S7**. Quantitative calculation of the assisted magnetization switching by IL-DMI during the SLS and DLS processes.

**Section S8**. Characterization of the IL-DMI effect for S_1 and S_2 with fixed IP field.

**Section S9**. Complete IL-DMI measurement results and comparisons for all the SFi devices.

**Section S10**. The procedure for extracting the threshold switching current and complete analog-like SOT switching of S_3 and S_2 under different IP fields.

**Section S11**. Detection of the intermediate resistance states of SFi S_1 during the SOT switching.

**Section S12.** SOT-induced Kerr-imaging dynamics and size-scaling results of the Hall bar devices.

**Section S13.** Non-volatile multi-resistance-state SOT switching results under different pulse widths.

**Section S14.** Characterization of the effective IL-DMI field of SFi S_2 for field-free SOT switching.

**Section S15.** Speculation on the reasons for the relatively small IL-DMI effective field measured during field-free SOT switching.

**Section S16.** Micromagnetic simulation results of magnetization dynamics during the binary-state SOT switching.

**Section S17**. Micromagnetic simulation results of the analog-like SOT switching.

**Section S18**. Quantitative calculation and simulation of the thermal contribution in the analog-like SOT switching process.

**Section S19**. Details and training process of the SNN constructed using analog-like SOT switching devices as neurons.

**Section S20**. Discussion on the LIF neuron model and SNN test accuracy.

**Section S21**. Energy consumption quantification and performance comparison of analog-like SOT devices applied to neurons of SNN.

**S1. The detailed procedure of asymmetric magnetization switching measurements.**

To provide readers with a clearer understanding of our measurement technique, we present the measurement procedure of the asymmetric hysteresis loops in detail. Figure S1 shows the schematic diagram of our measurement system. The device is mounted onto the sample holder using wire bonding. This sample holder can rotate within the x-y plane, allowing the application of additional in-plane fields at various IP angles. Meanwhile, the sample rod is positioned between the magnets and can rotate within the x-z plane, enabling both the sweeping of the out-of-plane magnetic field and the application of an IP field with a fixed direction.


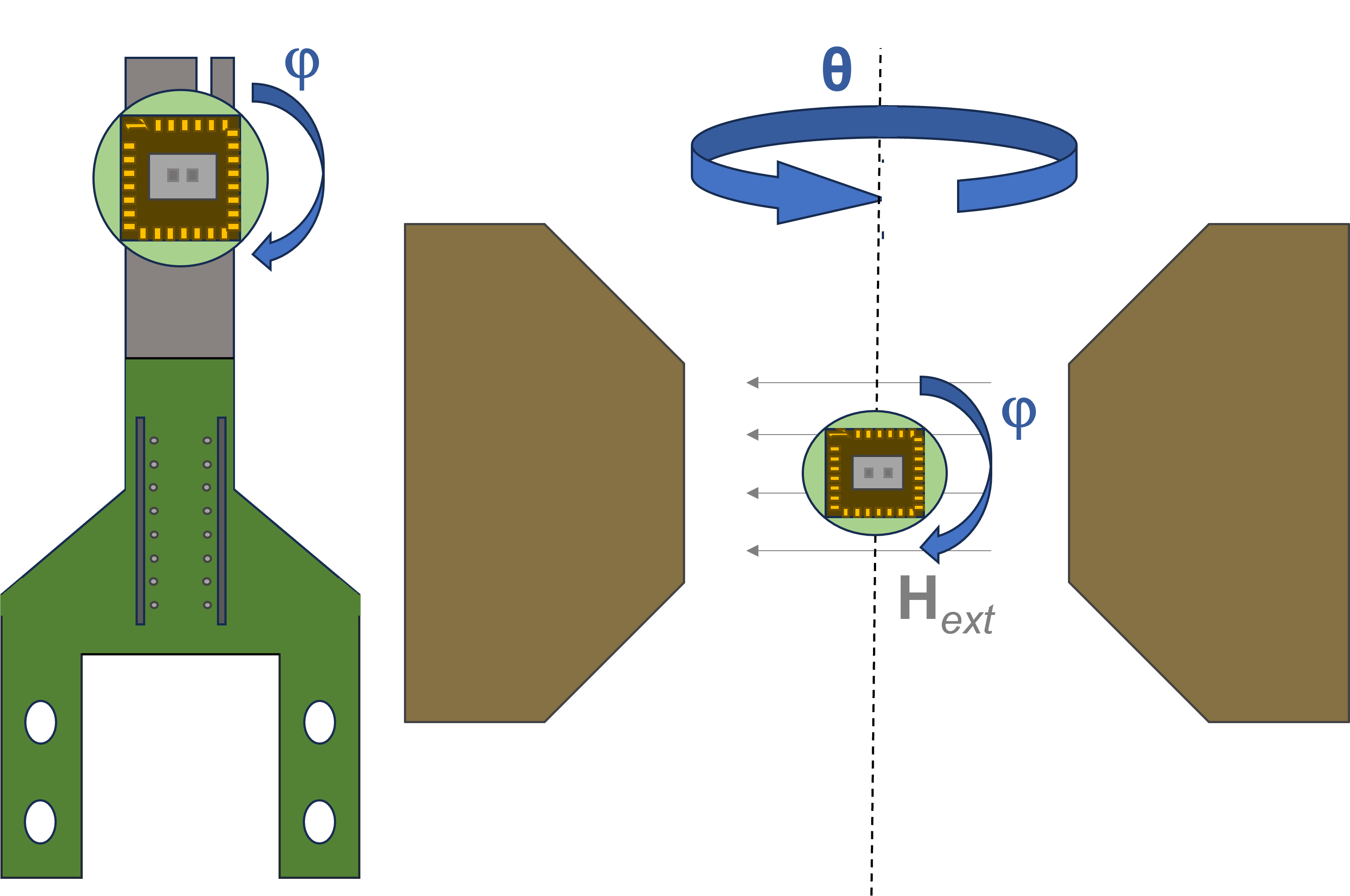


**Supplementary** **Figure S1**. **Schematic diagram of the measurement system for asymmetric hysteresis loop measurement.** The sample holder on the left can rotate in the x–y plane with a step size of 22.5°, while the sample rod on the right can rotate continuously in the x–z plane.

Figure S2 further illustrates the AHE measurement process under the application of in-plane magnetic fields at different angles. The left panel of Figure S2 shows the definition of the in-plane field angle and the rotation of the sample rod. We define *φ* as the angle between one end of the current axis of the Hall bar device and the x-axis. This angle is varied across the x-y plane in steps of 22.5°. For each fixed *φ*, the sample rod rotates within the x-z plane in the following manner: first clockwise from 0° to 180°, then counterclockwise from 180° back to 0°, thus completing a full forward and backward scan of the hysteresis loop. The right panel of Figure S2 shows the variations of the in-plane (IP) and out-of-plane (OOP) magnetic fields during this process, with the direction of the arrows indicating the rotation direction of the sample rod. At each angle *θ*, the IP magnetic field varies as *H_ext_ sinθ*, while the out-of-plane field varies as *H_ext_ cosθ*. We use a fixed value of external field *H_ext_* = 156 mT, which is slightly larger than the OOP saturation field of the sample. The bottom right panel of Figure S2 shows the typical AHE measurement curves as a function of the sample rod rotation angle *θ*. under different IP field application angles *φ*. Regardless of the value of *φ*, the sample is always subjected to the same variation process of the OOP and IP magnetic fields; the only difference lies in the direction of the IP field.


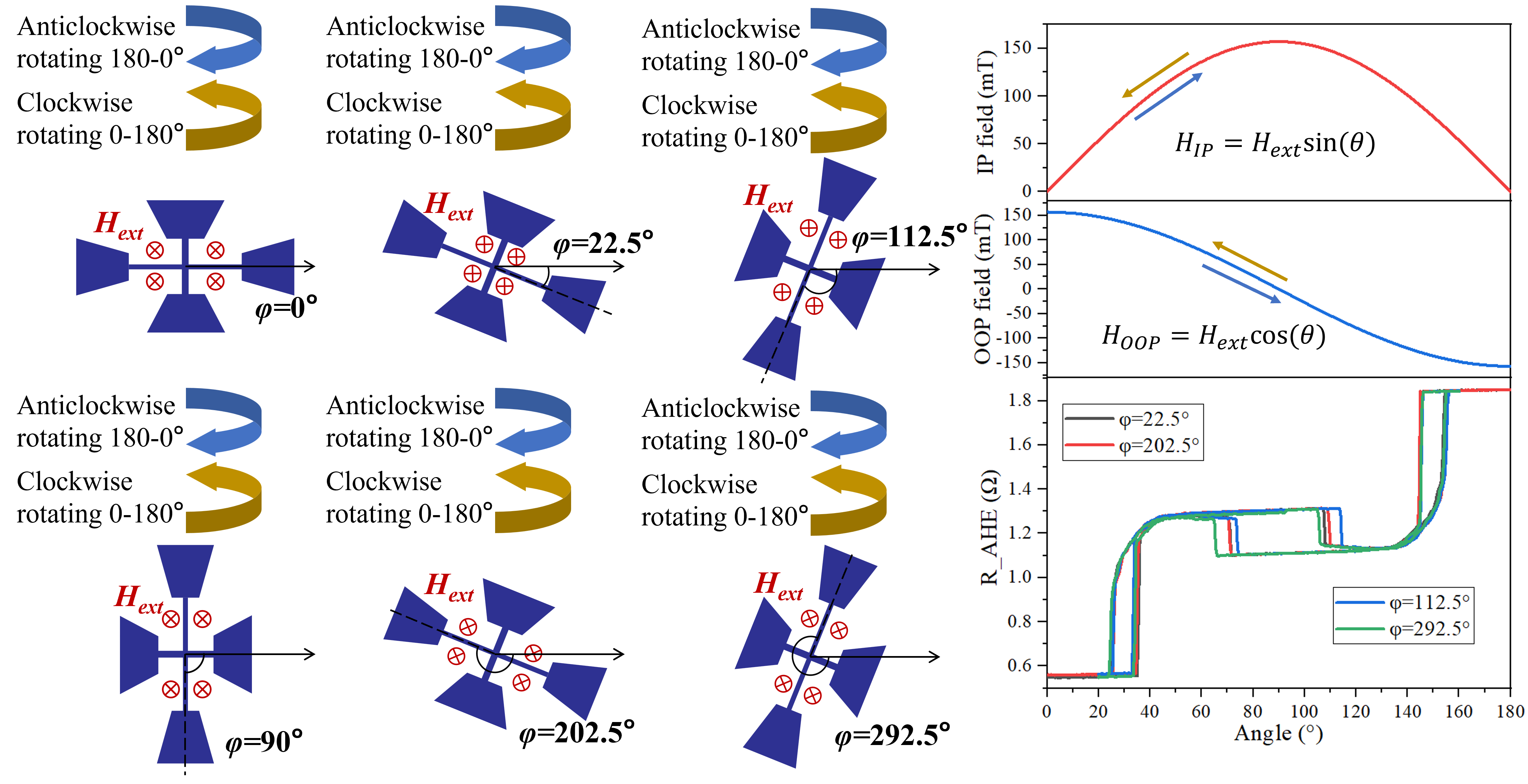


**Supplementary** **Figure S2**. **Typical measurement procedure of asymmetric AHE curves and the corresponding magnetic field variation process.**

**S2. The detailed switching process analysis and simulation of SFi in two step-switching.**

To provide a more comprehensive understanding of the asymmetric magnetization switching by IL-DMI in two-step switching, here we discuss magnetic hysteresis loops under an applied IP field *H_IN_* and IL-DMI. Figure S3a and b show the symmetric and asymmetric magnetization switching without or with IL-DMI respectively in a SFi. In the case without IL-DMI, positive or negative *H_IN_* will always assist the switching back and forth. In the case with both the IL-DMI and *H_IN_*, if the directions of the two are parallel, the switching process will be assisted; if the two directions are antiparallel, the switching process will be hindered. Figure S3c shows the corresponding hysteresis loop simulation without IL-DMI. The red and blue lines are superimposed. Figure S3d shows the azimuthal angular dependence of the switching field of the upper layer of the SFi. The U-D and D-U switching are totally symmetric. Figure S3e shows the corresponding hysteresis loop simulation with IL-DMI. Under the combined action of positive or negative *H_IN_* and IL-DMI, the hysteresis loops will show as an overall shift. The result is that the hysteresis loop appears to be shifted to left or right due to this assisting or hindering behavior. Figure S3f shows the azimuthal angular dependence of the switching field of the upper layer with IL-DMI. The ***D*** vector is set along the *y*-axis and the corresponding asymmetric axis is along *x* axis.


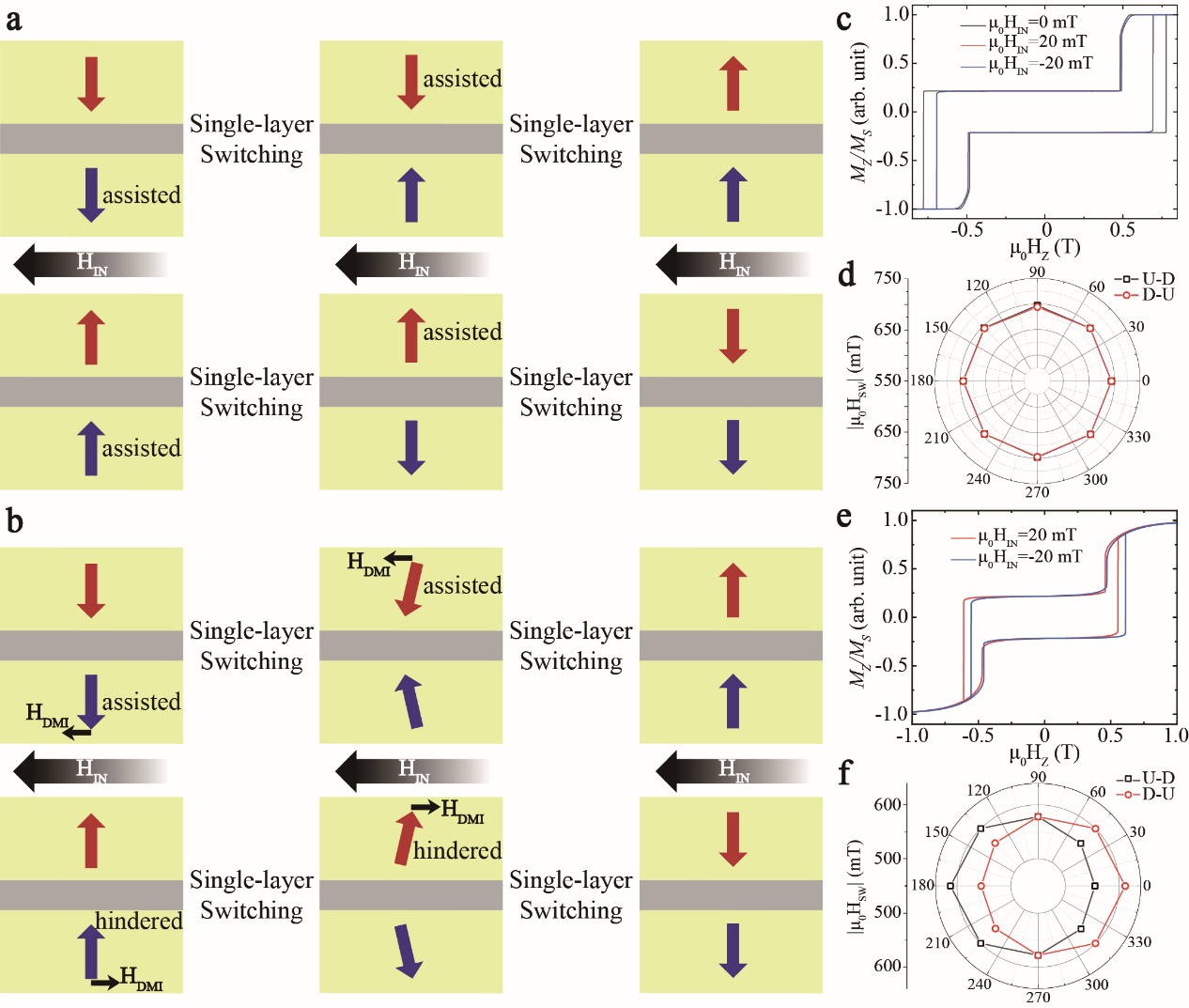


**Supplementary** **Figure S3**. **Symmetric and asymmetric magnetization switching without or with IL-DMI in a SFi with two-step switching.** (a-b) Schematic illustration of the switching process without (a) or with (b) IL-DMI in a SFi with two-step switching. In the case without the IL-DMI, all switches are assisted with *H_IN_* applied. When *H_IN_* is reversed, all switches are assisted too. In the case with IL-DMI, the switching processes during scanning back and forth are assisted and hindered respectively. When *H_IN_* is reversed, this assisting or hindering behaviour is also reversed. (c) Corresponding hysteresis loop simulation without IL-DMI in a SFi with two-step switching. Without the IL-DMI, the red and blue lines are superimposed. (d) Azimuthal angular dependence of the switching field of the upper layer of SFi without IL-DMI. The red and black lines are superimposed. (e) Corresponding hysteresis loop simulation with IL-DMI in a SFi with two-step switching. In the case with IL-DMI, the hysteresis loops with *H_IN_* of +100 mT and -100 mT are shifted to the left and right, respectively. (f) Azimuthal angular dependence of the switching field of the upper layer of SFi with IL-DMI. The AS axis and S axis are along 0° and 90° respectively.

Tilted anisotropy is indeed a possible effect that needs to be considered, especially in films deposited using oblique sputtering. To verify that the asymmetric magnetization reversal observed in our structure originates from IL-DMI rather than tilted anisotropy, we conducted additional micromagnetic simulations to exclude the influence of tilted anisotropy. Figure S4 provides a direct comparison between the two mechanisms and their distinct impacts on magnetization switching. Figure S4a illustrates the magnetic configuration induced by IL-DMI and its influence on each switching step in a two-step switching process (taking an example where the applied in-plane field points to the right). When the in-plane field is parallel to the IL-DMI effective field, switching is assisted, while an antiparallel field direction hinders the switching. Figure S4b displays the magnetic configuration produced by tilted anisotropy, where the magnetizations of both FM layers are tilted symmetrically by 180°, and the corresponding effect of an in-plane field directed to the right is analyzed. The switching behavior under tilted anisotropy is not consistent with that under IL-DMI, and therefore it does not lead to a rigid shift of the whole hysteresis loop. When decomposing the external in-plane field into parallel and perpendicular components relative to the magnetic moments, the parallel component may hinder switching while the perpendicular component may assist it, leading to uncertain switching behavior.

We further verified these differences through micromagnetic simulations. Figures S4c and S4d show the hysteresis loops obtained under IL-DMI and tilted anisotropy (with 𝐾 oriented along (0.4, 0, 1) here), respectively, when applying in-plane fields with varying azimuthal directions in the x–y plane. Figures S4e and S4g present the angular dependence of the switching fields of FM1 and FM2 under IL-DMI, both following cosine-shaped trends with opposite phase relationships. In contrast, Figures S4f and S4h show the switching-field distribution under tilted anisotropy, where FM2 exhibits a cosine-like trend but FM1 shows irregular behavior, and the FM1 U–D and D–U reversals do not show the opposite phase compared to FM2.

Our experimental results match the IL-DMI case shown in Figures S4e and S4g with high consistency. FM1 and FM2 follow cosine-shaped switching filed trends with opposite phase relationships (see Fig 2 b and c in the main text). This confirms that tilted anisotropy does not significantly contribute to the observed asymmetric switching in our devices. Additionally, the experimental results under a fixed in-plane field shown in Supplementary Section S8, as well as the asymmetric threshold-current distribution observed during SOT switching, cannot be explained by tilted anisotropy. These results further confirm that IL-DMI is the dominant magnetic interaction governing the switching behavior in our structure.


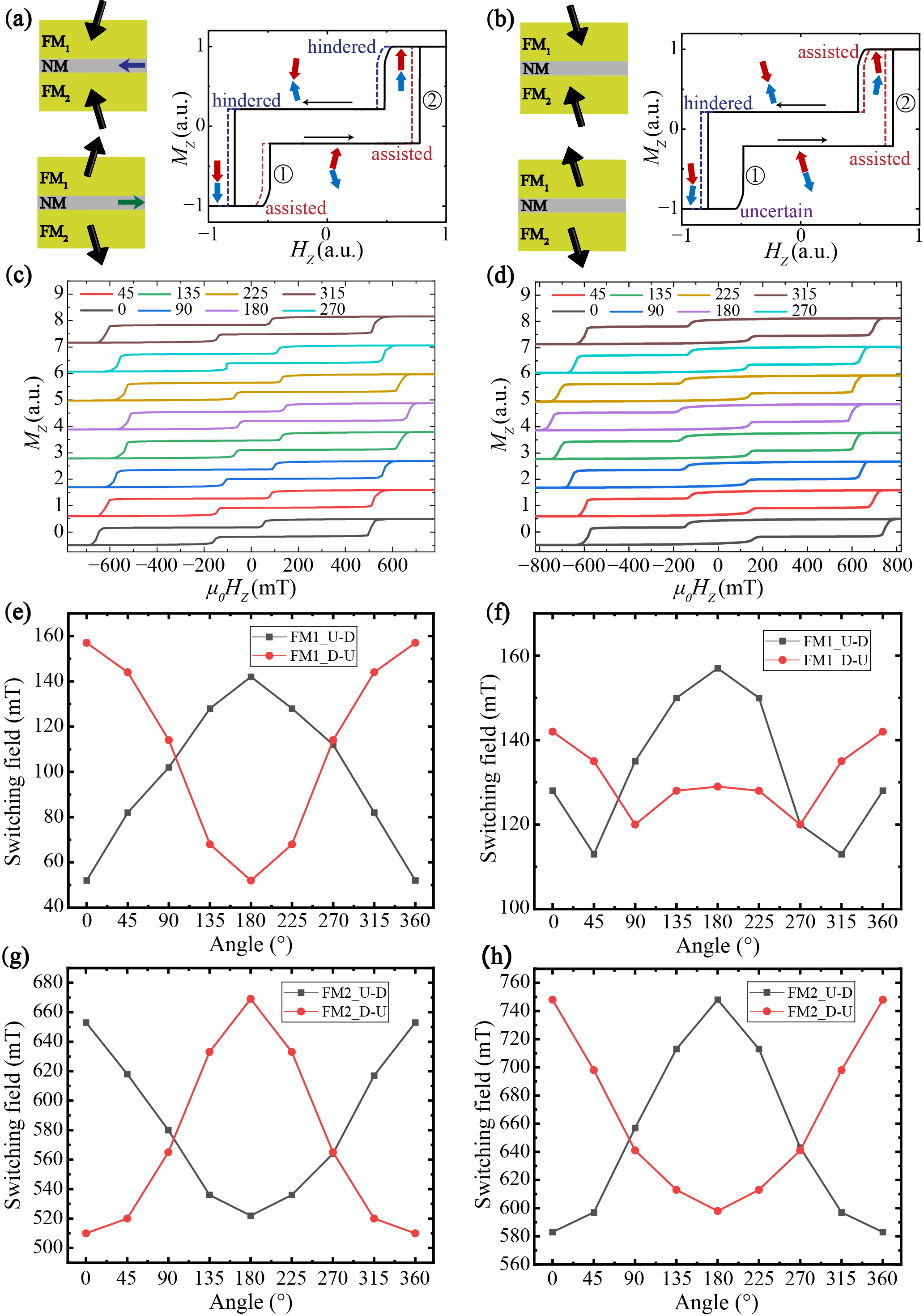


**Supplementary** **Figure S4**. (a and b) The magnetic configuration induced by IL-DMI (a) and tilted anisotropy (b) and their respective influence on each switching step. (c and d) The hysteresis loops obtained under IL-DMI (c) and tilted anisotropy (d) (with 𝐾 oriented along (0.4, 0, 1)), respectively. (e-h) The angular dependence of the switching fields of FM1 (e) and FM2 (g) under IL-DMI and the angular dependence of the switching fields of FM1 (f) and FM2 (h) tilted anisotropy.

**S3. The detailed switching process analysis and simulation of SFi in three-step switching.**

To provide a more comprehensive understanding of the asymmetric magnetization switching by IL-DMI with DLS (three-step switching), here we discuss magnetic hysteresis loops under an applied IP field *H_IN_* without or with IL-DMI. Figure S5a and b show the symmetric and asymmetric magnetization switching without or with IL-DMI respectively in a SFi with three-step switching. Without IL-DMI, positive or negative *H_IN_* will always assist the switching back and forth. In the case with both the IL-DMI and *H_IN_*, the switching will be assisted or hindered depending on the direction of *H_IN_* and magnetization configuration. Figure S5c shows the corresponding hysteresis loop simulation without IL-DMI. The red and blue lines are superimposed. Figure S5d shows the corresponding hysteresis loop simulation with IL-DMI. The three loops will undergo an expanding-shifting-contracting process. Accordingly, the center of the two outer loops can be used to calibrate the angular error. The shifting of the inner loops can be used to characterize the IL-DMI. It should be pointed out that in the simulation results here, the shift of the inner loop is not significantly amplified, which is different from the measured experimental results. This is because the Arrhenius law [1] does not apply to our Mumax simulation. Our micromagnetic simulation only considers single-domain uniform switching at the nanometer scale at a temperature of 0 K, while the switching results of the μm-level devices in the experiment are caused by thermally activated magnetization switching. Figure S5e shows the azimuthal angular dependence of the switching field from AP+ to AP- and AP- to AP+. The ***D*** vector is set along the *y*-axis direction, and the corresponding asymmetric axis is along the *x*-direction.


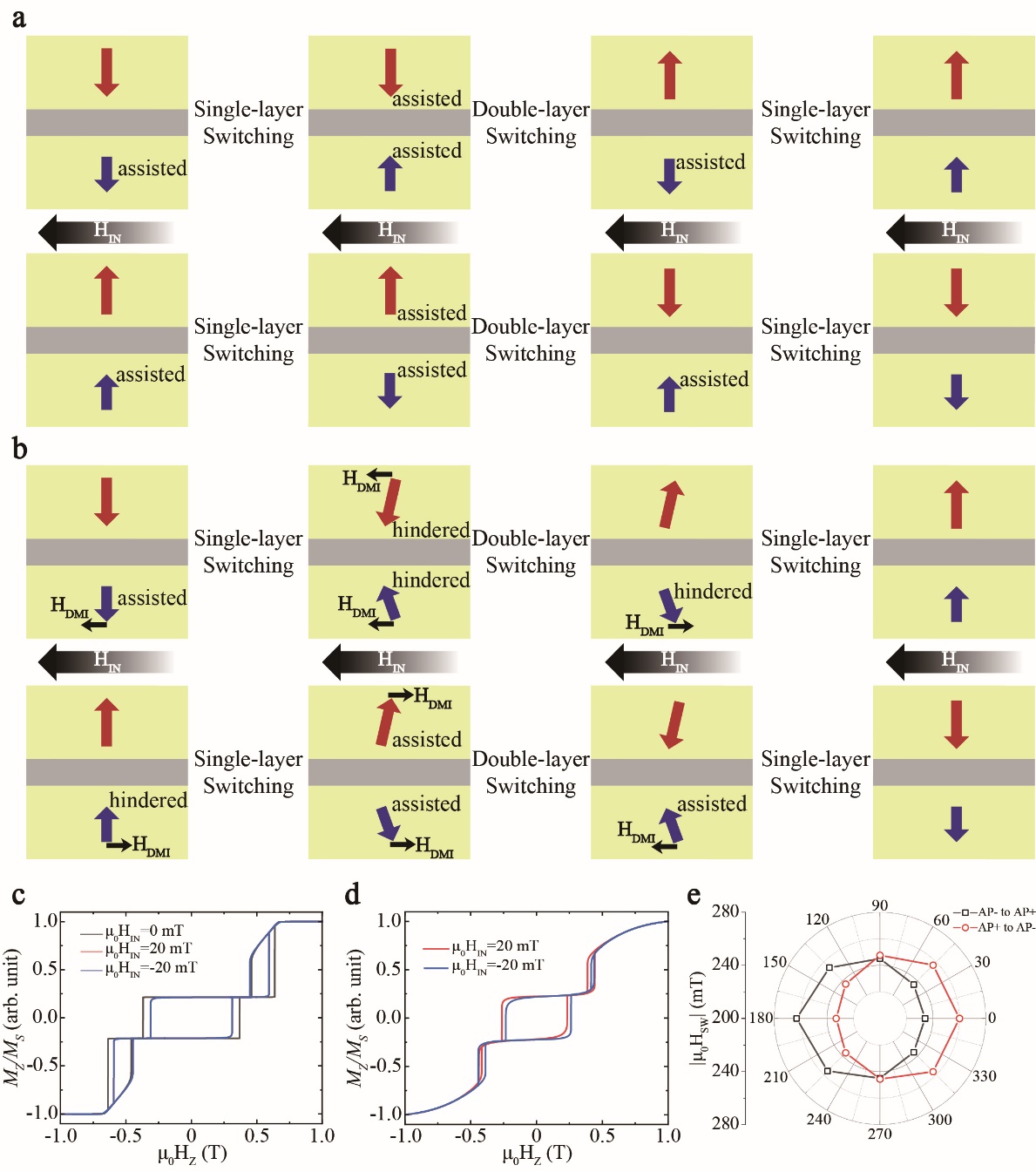


**Supplementary** **Figure S5**. **Symmetric and asymmetric magnetization switching without or with IL-DMI in a SFi with three-step switching.** (a-b) Schematic illustration of the switching process without (a) or with (b) IL-DMI in a SFi with three-step switching. Without IL-DMI, all switches are assisted with *H_IN_* applied. When *H_IN_* is reversed, all switches are assisted too. In the case with IL-DMI, depending on the magnetization state of the upper and lower layers, there are both assisting or hindering behaviours during the entire switching process. When *H_IN_* is reversed, this assisting or hindering behaviour is also reversed. (c-d) Corresponding hysteresis loop simulation without (c) or with (d) IL-DMI in a SFi with DLS process. Without the IL-DMI, the red and blue lines are superimposed. With the IL-DMI, the switching field during the DLS process is shifted to left and right, respectively. During the SLS, depending on the assisting or hindering behavior, the switching field width will expand or contract, respectively. (e) Azimuthal angular dependence of the switching field during DLS of SFi with IL-DMI. The AS axis and S axis are along 0° and 90° respectively.

After completing the analysis and micromagnetic simulation of the switching process of SFi in three-step switching, we focus on analyzing the role of IL-DMI in the DLS process. In SLS, it is easy to analyze whether IL-DMI assists or hinders the switching. Since the existence of an IP field will help define the switching direction of the FM and reduce the energy barrier required for switching, IL-DMI will always assist the switching when *H_IL-DMI_* is parallel to the external IP field and hinder the switching when *H_IL-DMI_* is antiparallel to the external IP field. However, in DLS process, the analysis of whether IL-DMI assists or hinders the switching becomes complicated. Since the upper and lower FMs are antiferromagnetically coupled and will switch at the same time, both layers will switch with the same clock direction to maintain the antiparallel arrangement during the whole switching process. This means that *H_IL-DMI_* parallel to the external IP field will always assist the switching of one layer and hinder the switching of the other layer. It will be complicated to analyze the role of IL-DMI in DLS using the way IL-DMI is analyzed in SLS. Here, we change the method to make the switching process analysis clearer. Figure S6a and b show the schematic illustration of the DLS switching process under IL-DMI and an extra IP field when *H_Z_* is ascending and descending, respectively. During the actual measurement process, *H_IN_* is much larger than *H_IL-DMI_*. Because the upper and lower layers will switch at the same time during the DLS process, this will inevitably lead to a situation where both of them rotate into IP direction. We mainly consider the relative directions of *H_IL-DMI_* and vertical scanning field *H_Z_* in this case. When DLS happens, the magnetic moment of the thinner (lower) layer will always turn to *H_IN_* direction first. This process is similar to the first step in the three-step switching process. The smaller magnetic moment makes that the lower layer is more easily driven by the influence of external fields. At this time, due to the antiferromagnetic coupling, the magnetic moment of the upper layer will rotate to the anti-parallel IP direction relative to the lower layer magnetic moment. Considering the chirality of IL-DMI, *H_IL-DMI_* in this case will point vertically downward. DLS occurs in the positive (negative) magnetic field region when *H_Z_* is ascending (descending). Correspondingly, *H_IL-DMI_* hinders (assists) the DLS since it is antiparallel (parallel) to *H_Z_*.


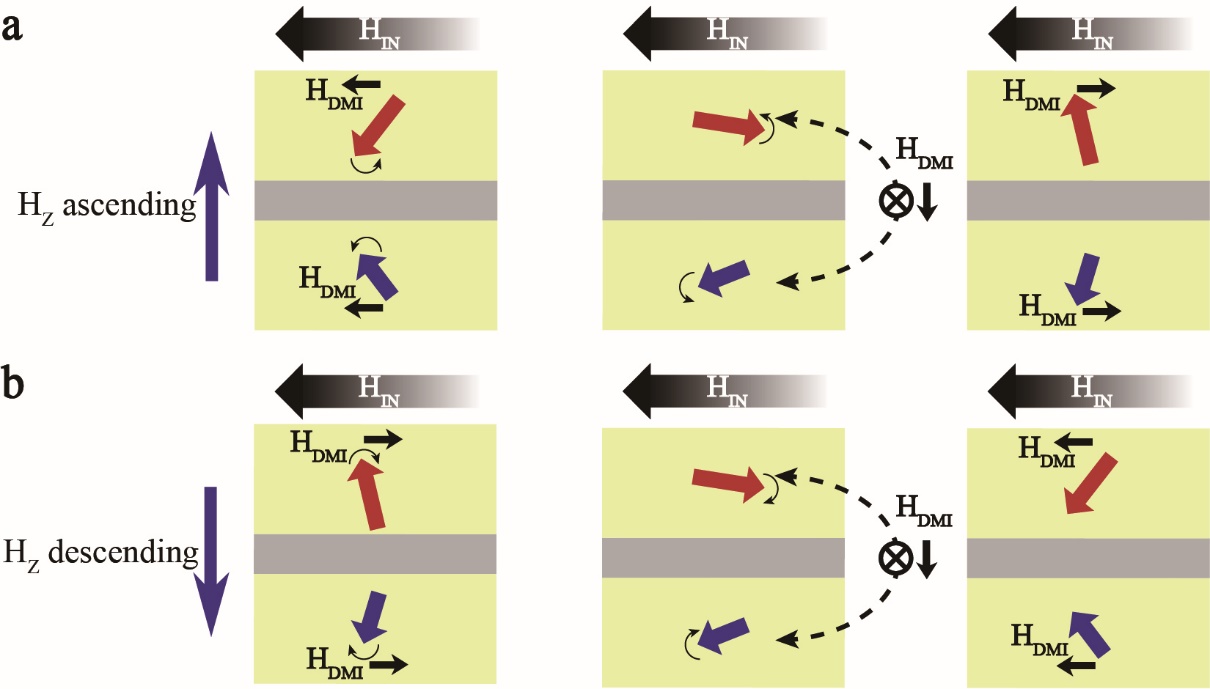


**Supplementary** **Figure S6**. **Analysis of the role of IL-DMI in the DLS process. (**a-b) Schematic illustration of the DLS switching process under IL-DMI and an extra IP field when *H_Z_* is ascending (a) or descending (b). When DLS happens, the magnetic moment of the thinner (lower) layer will always turn to *H_IN_* direction first. The magnetic moment of the upper layer will rotate to the anti-parallel IP direction. In both cases, *H_IL-DMI_* will point vertically downward. Correspondingly, *H_IL-DMI_* hinders (assists) the DLS since it is antiparallel (parallel) to *H_Z_* when *H_Z_* is ascending (descending).

Previously reported asymmetric magnetization switching induced by IL-DMI lacked the synchronous switching process between the two antiparallel states, i.e., the double-layer switching. However, this double-layer switching is a critical magnetization reversal step in SFi. It represents the mutual reversal between the two energetically most stable states of SFi, corresponding to the bit “0” and bit “1” states in data storage applications. For the first time, we report the asymmetric switching behavior induced by IL-DMI during this double-layer switching process. Moreover, we find that this synchronized switching can amplify the IL-DMI offset field by approximately 20 times, significantly facilitating future IL-DMI measurements, as shown in Fig. S7.


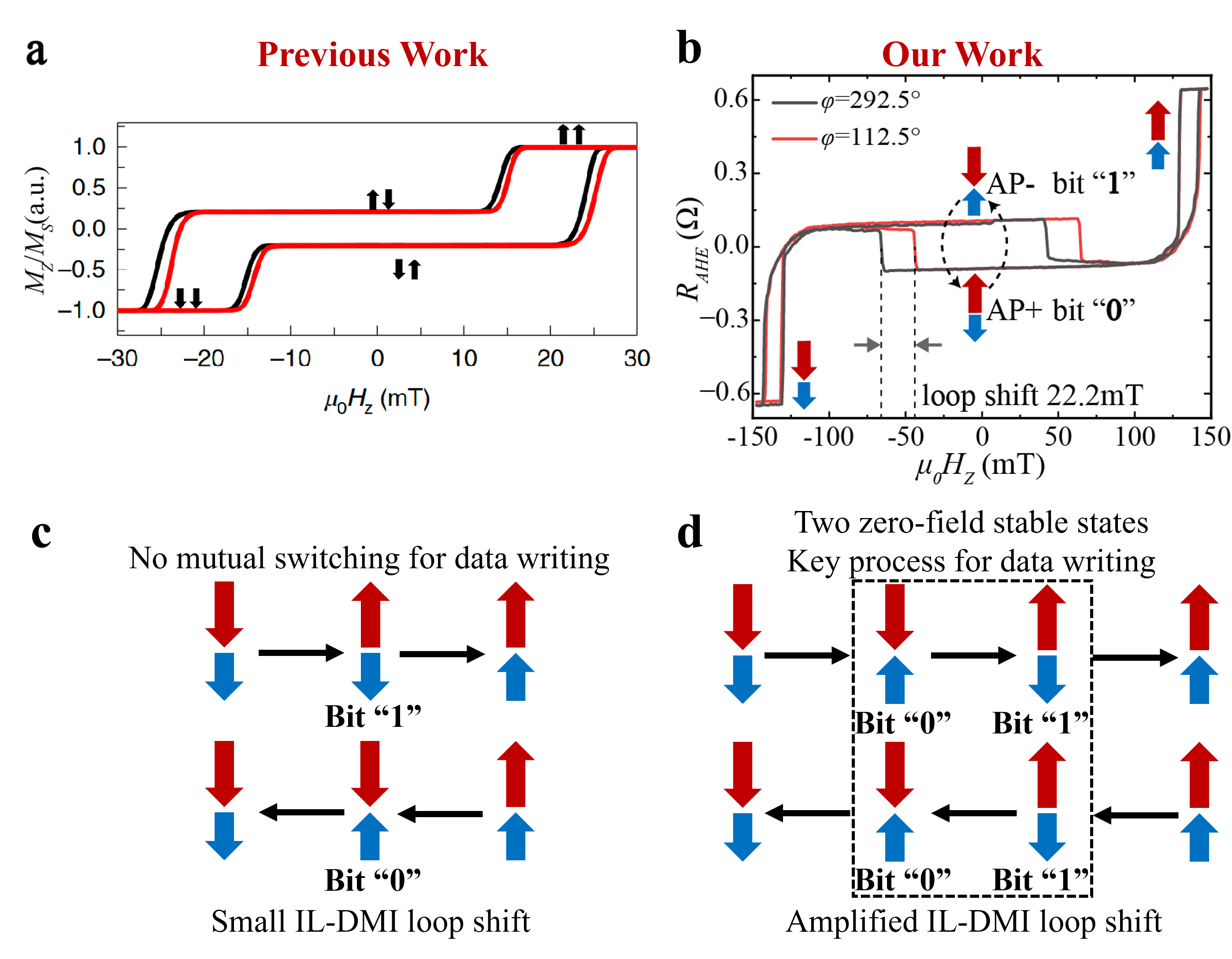


**Supplementary** **Figure S7**. (a and b) The asymmetric magnetization switching without/with double-layer switching induced by IL-DMI in [7] (a) and in our work (b); (c and d) Schematic diagrams of the magnetization switching processes corresponding to the hysteresis loops in a (c) and b (d).

**S4. Basic film, magnetic and electrical transport properties of SFi S_1 to S_3**

The basic magnetic and electrical transport properties of our samples S_1 to S_3 are shown in this section. Figure S8 shows the magnetic moment measurement results using SQUID. The AHE signal of the antiparallel state changes sign between S_3 and S_2 due to the increase in the thickness of the underlying Co layer, which makes the SOC between Co and Pt stronger [3]. Figure S9 shows the AHE signal contrast of S2 under IP and OOP field. Due to the weaker PMA of the lower Co, the magnetic moment is easier to rotate into the X-Y plane under the applied IP field.


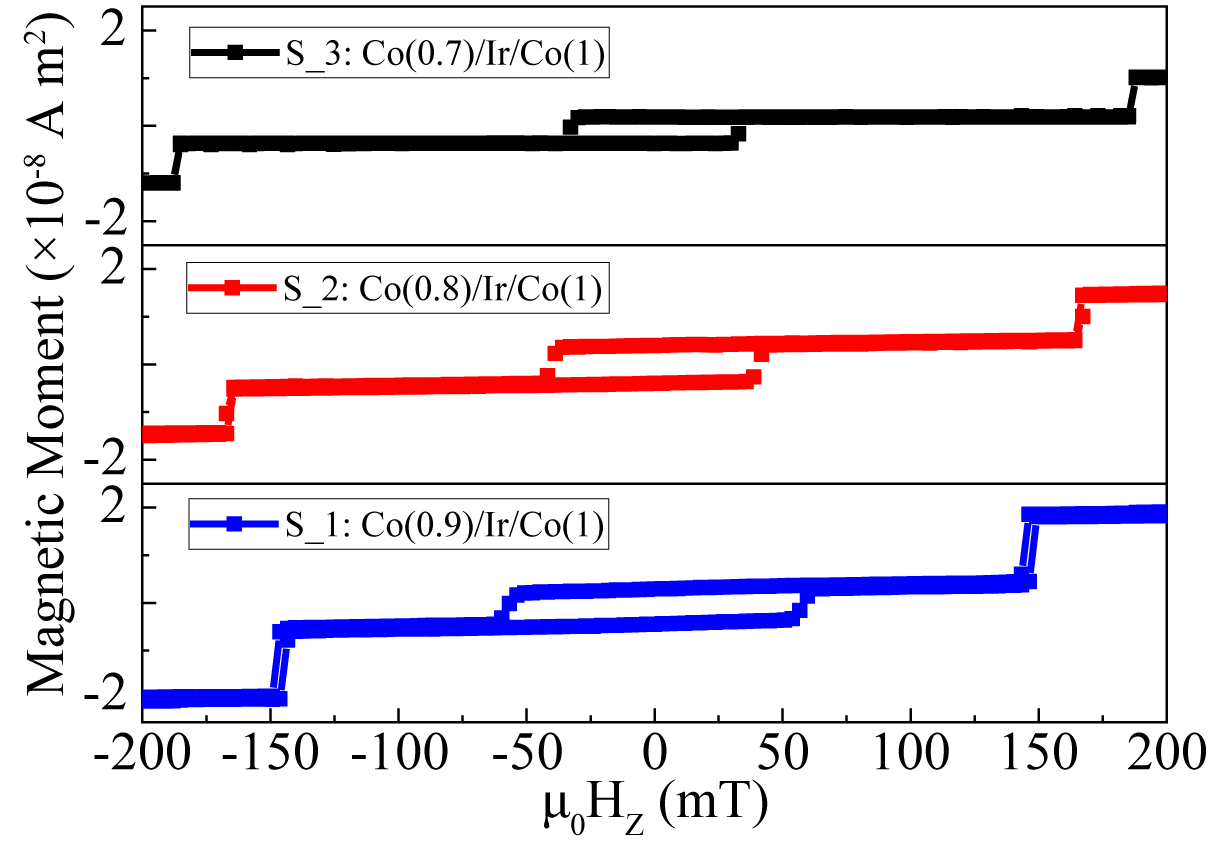


**Supplementary** **Figure S8**. **Basic magnetic and transport properties of SFi S_1 to S_3 under OOP magnetic field.** (a) Magnetic moment measurement results using SQUID.


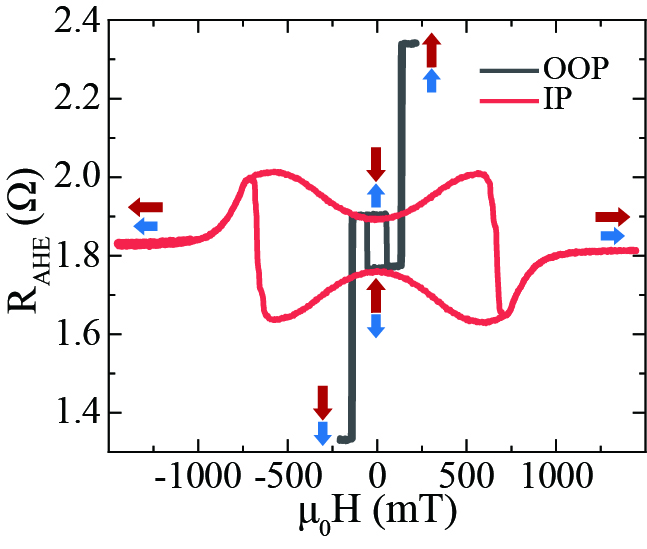


**Supplementary** **Figure S9**. **The AHE signal of S_2 under both IP and OOP field.** The red and blue arrows indicate the magnetization states of the upper and lower layer under some specific fields, respectively.

Figures S10a and b show optical microscopy images of a representative Hall-bar device (2-µm width). Figure S10c presents the surface morphology of the Ta/Pt/Co/Ir/Co/Cu/Ta stack before Au-electrode deposition, together with its characteristic roughness. Bruker AFM measurements indicate a surface roughness of Rq = 0.0515 nm and Ra = 0.0409 nm (highlighted by the red dashed box), demonstrating the good flatness of the film, which is also smaller than the Co-layer thickness resolution of our samples (0.1 nm). This ensures reliable conditions for subsequent hysteresis-loop, AHE and SOT measurements.


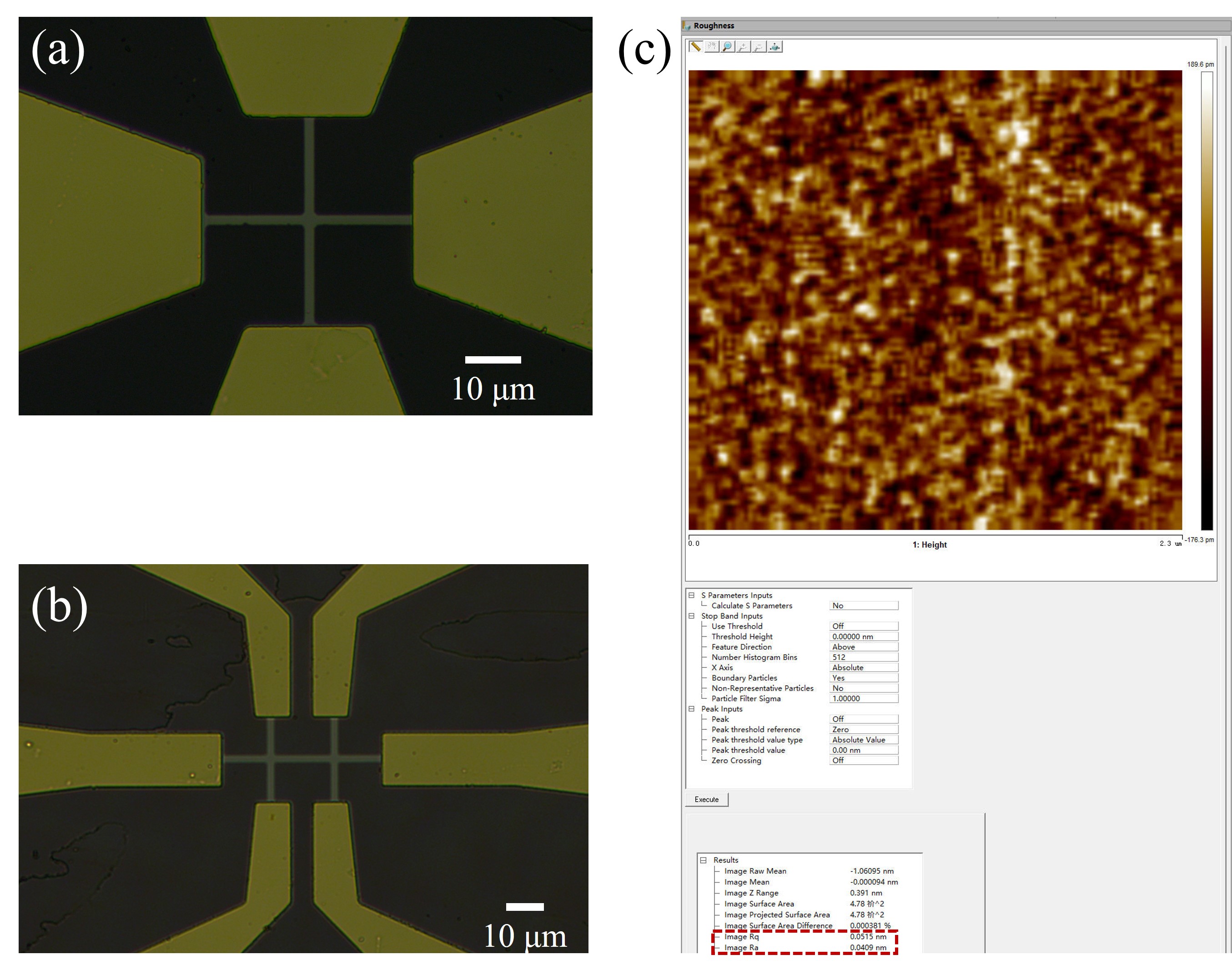


**Supplementary** **Figure S10**. (a and b) Optical microscopy images of our representative Hall-bar devices. (c) The surface morphology of the Ta/Pt/Co/Ir/Co/Cu/Ta stack.

**S5. Comparison between the minimum and maximum switching field shifts of SFi S_2 during the DLS process.**

To verify the IL-DMI in SFi with three-step switching, we measured AHE loop with an extra IP field along different direction. Figure S11 shows the hysteresis loops of S_2 near both the asymmetry axis and symmetry axis. When *H_IN_* is along *φ*=22.5° and *φ*=202.5°, the AHE loop curves almost overlap. When *H_IN_* is along *φ*=112.5° and *φ*=292.5°, the inner loops for DLS are clearly shifted to left and right, respectively. The maximum loop shift measured here is 22.2 mT.


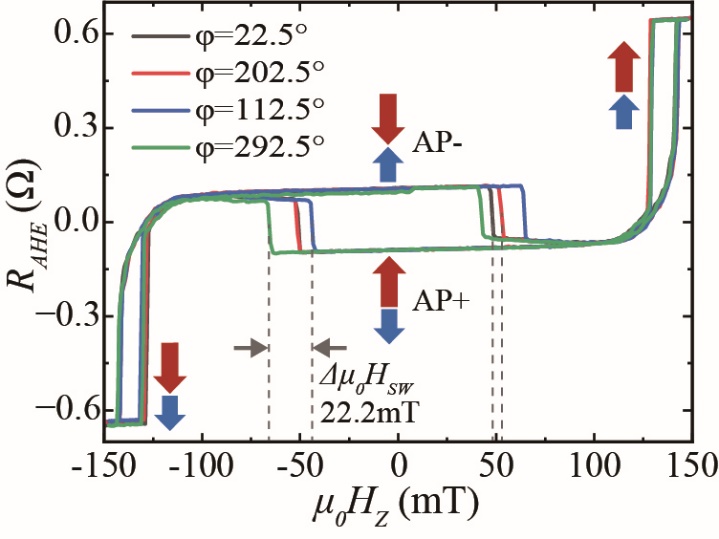


**Supplementary** **Figure S11**. **Minimum and maximum switching field shifts of SFi S_2 during the DLS process measured at azimuth angles of 22.5°, 202.5° and 112.5°, 292.5°.** When *H_IN_* is along *φ*=22.5° and *φ*=202.5°, the AHE loop curves almost overlap. When *H_IN_* is along *φ*=112.5° and *φ*=292.5°, the inner loops for DLS are clearly shifted to left and right, respectively, relative to the inner loops along *φ*=22.5° and *φ*=202.5°.

**S6. Characterization of the IL-DMI effect for SFi with three-step switching by measuring the switching field width during SLS process.**

To further verify the IL-DMI in SFi with DLS, we also measured the switching field difference in SLS process of the outer loops. Figure S12a shows the hysteresis loops of S_2 near the asymmetric axis. Figures on the right side are enlarged views of the corresponding outer loops under positive and negative fields. Taking the curve at *φ*=112.5° as an example, its outer loop width at SLS will contract and expand under negative and positive fields respectively, which is consistent with our analysis and simulation results in Fig. S5. Since the outer loops will appear to expand or contract rather than shifting, we can use the center of the outer loops as the reference axis to calibrate the hysteresis loop, thereby eliminating errors caused by angular inaccuracies or field misalignments. Figure S12b shows the azimuthal angular dependence of the outer loop width during the SLS process of S2. The distribution of symmetry axis and asymmetry axis is basically consistent with the results shown in Figure 2e in the main text.


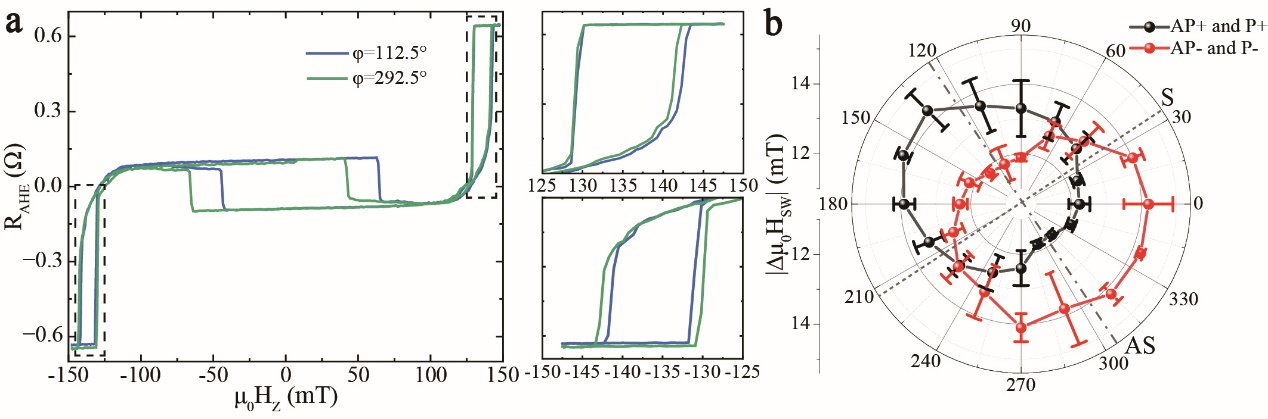


**Supplementary** **Figure S12**. **Characterization of the IL-DMI effect for SFi with three-step switching in the SLS process. (**a) Comparison of hysteresis loops of S2 near the asymmetric axis. The figures on the right show the enlarged curve in the dashed box in the left figure. The two switching behaviours are consistent with the switching analysis and simulation curves in Figure S5. (b) Azimuthal angular dependence of the outer loop width during the SLS process of S2. The red and black data represent the loop width at left and right side in a, respectively. The maximum loop width difference is 2.46 (±0.7) mT. Similarly, we can calculate that the effective IL-DMI field in S2 is $\frac{\left| \Delta\mu_{0}H_{SW} \right|}{2}=$1.23 mT. The distribution of S axis and AS axis is basically consistent with the results shown in Figure 2e in the main text.

**S7. Quantitative calculation of the assisted magnetization switching by IL-DMI during the SLS and DLS processes.**

Based on the theoretical explanation of IL-DMI-assisted SLS and DLS and our experimental results, we quantitatively calculated the performance of IL-DMI-assisted switching according to the Arrhenius law [1,4]. Here, we use an extended model for calculating the switching field of the SFi. The interlayer coupling energy, magnetic energies of two layers, and anisotropy energies are all taken into account [5]. For AP+ to AP- transition,

$$\begin{aligned} H_{C}=H_{f}ln\left( \frac{R}{R_{0}} \right)+\frac{K_{eff}}{2M_{S}}\frac{h_{2}+h_{1}}{h_{2}-h_{1}}\#\left( 1 \right) \end{aligned}$$

$$\begin{aligned} H_{f}=\frac{kT}{2M_{S}V_{A}}\frac{h_{2}+h_{1}}{h_{2}-h_{1}}\#\left( 2 \right) \end{aligned}$$

where $H_{f}$ is the fluctuation field, R = *dH/dt* is the sweeping rate of magnetic field, $K_{eff}$ is the magnetic effective anisotropy, $M_{S}$ is the saturation magnetization, *V_A_* is the activation volume, *h_1_* and *h_2_* are the thicknesses of top and bottom layers, respectively and *R_0_* is a parameter proportional to the attempt frequency (Arrhenius factor) of domain nucleation. For SLS and DLS, the differences in energy terms not only include the thickness of the magnetic films but also the contribution of the effective IL-DMI field to effective anisotropy. For SLS, the IL-DMI and the applied in-plane field combine to lower the energy barrier in the form of an in-plane field [6],

$$\begin{aligned} K_{eff}=\frac{1}{2}\mu_{0}H_{K,eff}M_{S}\left( 1-\left( \frac{H_{X}+H_{IL-DMI}}{H_{K,eff}} \right)^{2} \right)\#\left( 3 \right) \end{aligned}$$

For DLS, there is a situation where the magnetic moments of the upper and lower layers simultaneously rotate in-plane, in which case the IL-DMI directly reduces the energy barrier in the form of a perpendicular field,

$$\begin{aligned} K_{eff}=\frac{1}{2}\mu_{0}{(H}_{K,eff}{-H_{IL-DMI})M}_{S}\left( 1-\left( \frac{H_{X}}{H_{K,eff}} \right)^{2} \right)\#\left( 4 \right) \end{aligned}$$

By substituting the anisotropy terms into Equation (1), we obtain the switching field *H_C_* of SFi device as a function of *H_IL-DMI_*, as shown in Figure S13a. The black line represents the reference switching field without in-plane field and IL-DMI, which is 51.63 mT, close to the actual experimental value (53.7 mT). It can be observed that with the increase of the IL-DMI effective field, *H_C_* for both the SLS and DLS processes gradually decreases, with the decrease in DLS being more significant. Figure S13b shows the ratio of the reduced switching field in DLS $\Delta H_{DLS}$ to that in SLS $\Delta H_{SLS}$ as a function of IL-DMI. As *H_IL-DMI_* increases, this ratio gradually rises to 15 times, which is close to our experimental results.


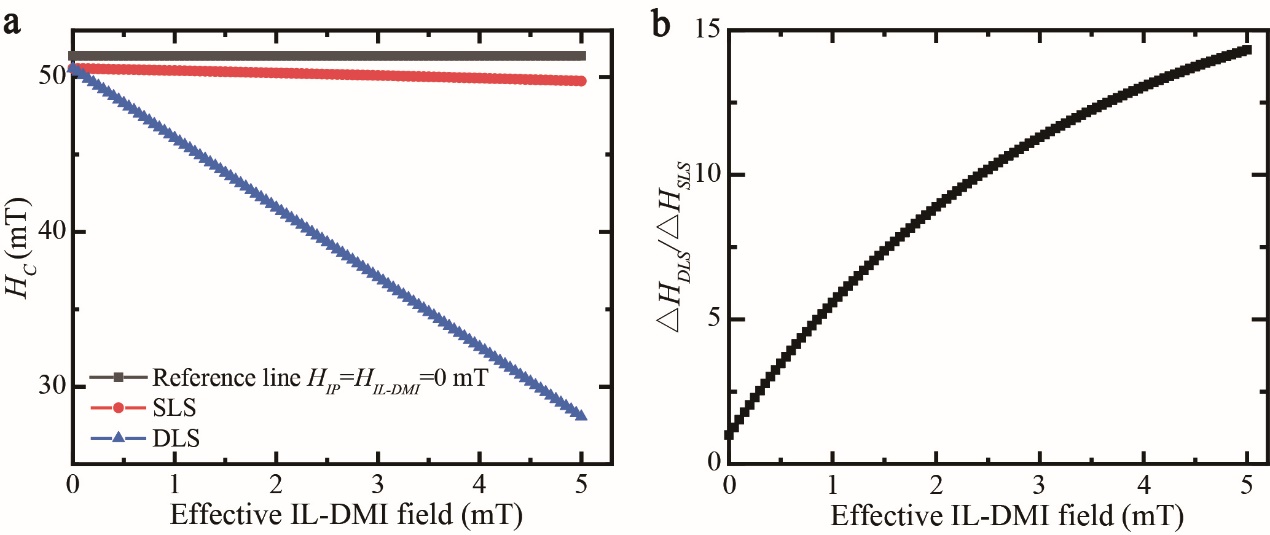


**Supplementary** **Figure S13**. **Calculation of the switching field *H_C_* with IL-DMI during the SLS and DLS processes. (**a) The trend of *H_C_* decreasing with increasing IL-DMI in both the SLS and DLS processes. (b) The variation of the ratio between the reduced switching field in DLS and the reduced switching field in SLS with IL-DMI.

**S8. Characterization of the IL-DMI effect for S_1 and S_2 with fixed IP field.**

In our asymmetric magnetization switching measurements, the direction of the additional IP field is fixed, while its magnitude varies with the rotation angle *θ*, following $H_{IN}=H_{ext}sin\theta$. In contrast, in previous study [7], both the magnitude and direction of the IP field remained constant. However, as seen from the results in Figure 3b of [7], the influence of the IP field magnitude on the switching field shift is relatively small—the switching field changes by only about 1.3 mT when the IP field increases from 0 mT to 112.5 mT. While the IP field can shift the hysteresis loop, it cannot account for the roughly 20-fold enhancement of the switching field in double layer switching (DLS) process compared to single layer switching (SLS) observed in our experiment. Moreover, we are not the first to use this method to measure the IL-DMI; similar approaches have been employed in earlier studies as well [8][9]. Next, we performed additional AHE measurement experiments with the IP field fixed in both magnitude and direction, further confirming the validity of our measurement method.

To confirm the effect of the IP field magnitude on the switching field of our device, we supplemented our study with AHE measurements under various constant IP fields. Figure S14 shows the AHE curves of device S_2 under constant IP fields applied along the asymmetric (AS) axis. As the IP field magnitude increases, the switching field shifts of both the DLS and SLS also increase.


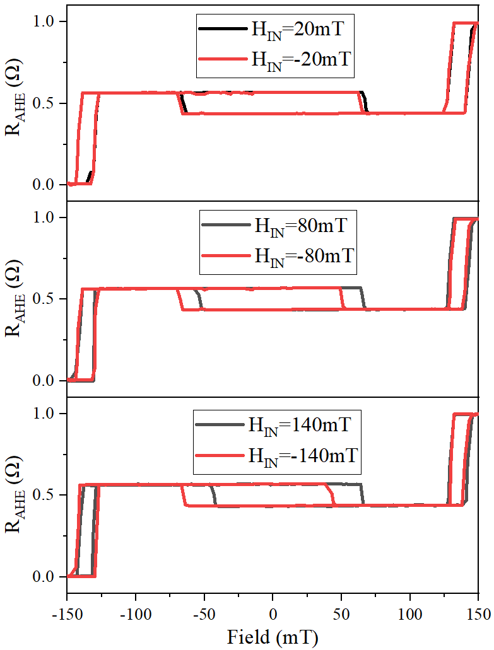


**Supplementary** **Figure S14. AHE curves of device S_2 under constant IP fields applied along the AS axis.**

Furthermore, Figure S15 presents the experimentally measured switching field *μ*_0_*H_SW_* as a function of *H_IN_* in S_2. The top panel shows the switching field variations of different switching processes (AP+ to AP-, AP+ to P+, P+ to AP+) with the IP field applied along the AS axis, while the bottom panel shows the same with the IP field applied along the S axis. When the IP field reaches 140 mT, the DLS exhibits a switching field shift of 22.4 mT, while the average shift for the SLS is only 1.85 mT. This indicates that the switching field shift in DLS is still enhanced by roughly 12 times compared to that in SLS.


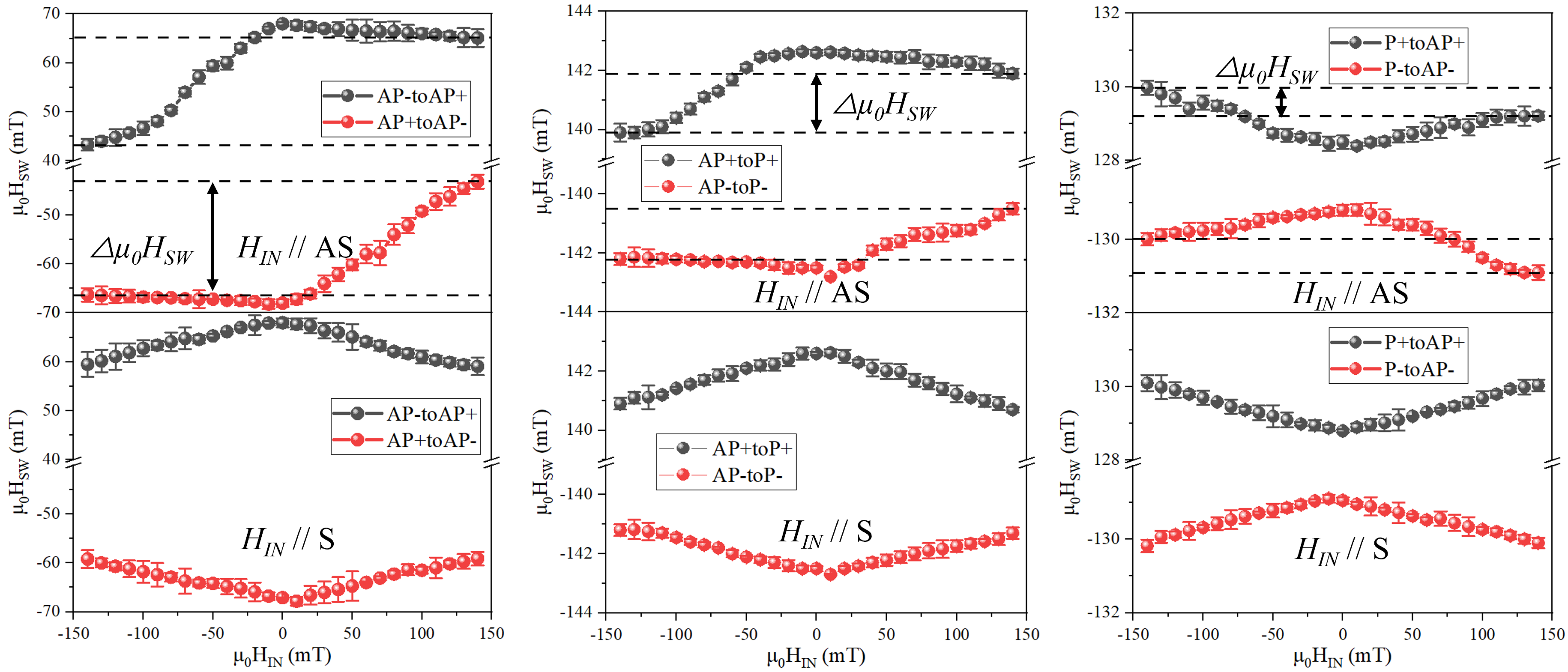


**Supplementary** **Figure S15. The experimentally measured switching field as a function of *H_IN_* in S_2.**

In contrast, figure S16 shows the experimentally measured switching field *μ*_0_*H_SW_* as a function of *H_IN_* in S_1. The maximum (*μ*_0_*H_IN_=*140 mT) switching field shifts are 2.94 mT and 2.1 mT, respectively, which are of the same order of magnitude as the results presented in the main text (2.8 mT).


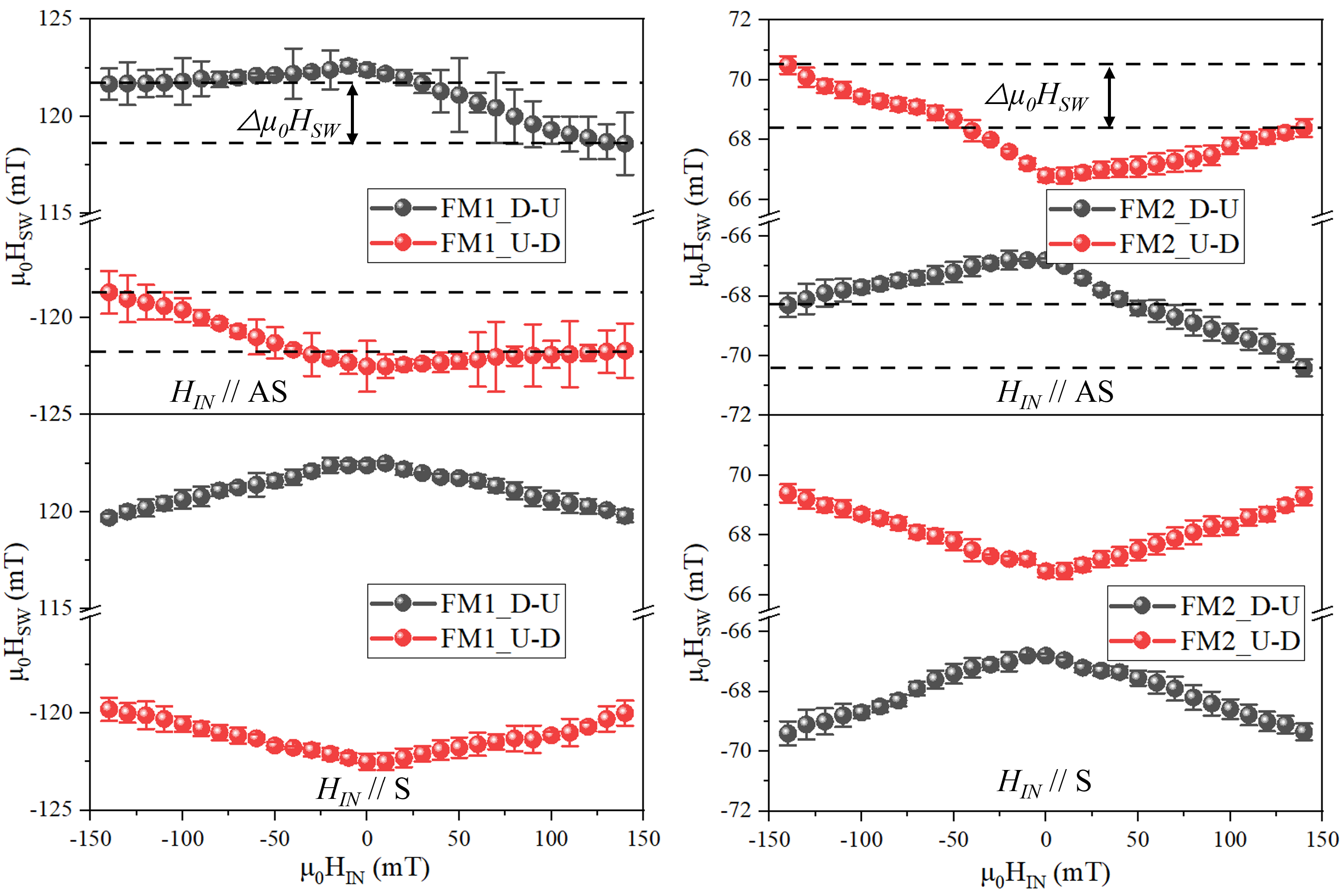


**Supplementary** **Figure S16. The experimentally measured switching field as a function of *H_IN_* in S_1.**

In summary, we have supplemented our study with AHE measurements under constant IP fields, which further support our core conclusion: DLS exhibits a significantly enhanced switching field shift induced by IL-DMI compared to SLS. Previous studies have validated the effectiveness of our measurement method, and the consistency between our supplementary experimental data and the results in the main text further confirms this conclusion.

**S9. Complete IL-DMI measurement results and comparisons for all the SFi devices.**

Here, we provide complete measurement results from SFi devices with different uncompensated magnetic moments. We prepared three samples, S_1, S_2, and S_3, where the numbers indicate the thickness differences in angstroms between the upper and lower ferromagnetic layers. We also attempted samples with thickness differences of 4 Å and 5 Å; however, in these cases, the lower Co layer thickness was reduced to 0.6 nm and 0.5 nm, respectively. Due to the excessively thin Co layer, the PMA is significantly weakened, and the anisotropy tends to become in-plane, which falls outside the scope of our study.

Figure S17 shows the measurement results of the asymmetric hysteresis loops of the S_3 device. Figure S17a presents typical AHE curves under in-plane fields applied along the symmetric and asymmetric axes. Figure S17b shows the azimuthal angular dependence of the switching field during the DLS process of S_3. AP+ and AP− represent the antiparallel states where the magnetization in the upper Co layer points up and down, respectively. From these results, the AS axis and S axis of the S_3 device is along 135° and 45°, respectively, with the maximum hysteresis loop shift reaching 23.1 mT. The maximum switching field shift during the SLS process is 1.63 mT. This further confirms that, compared to SLS, the DLS process can significantly amplify the hysteresis loop shift induced by IL-DMI.


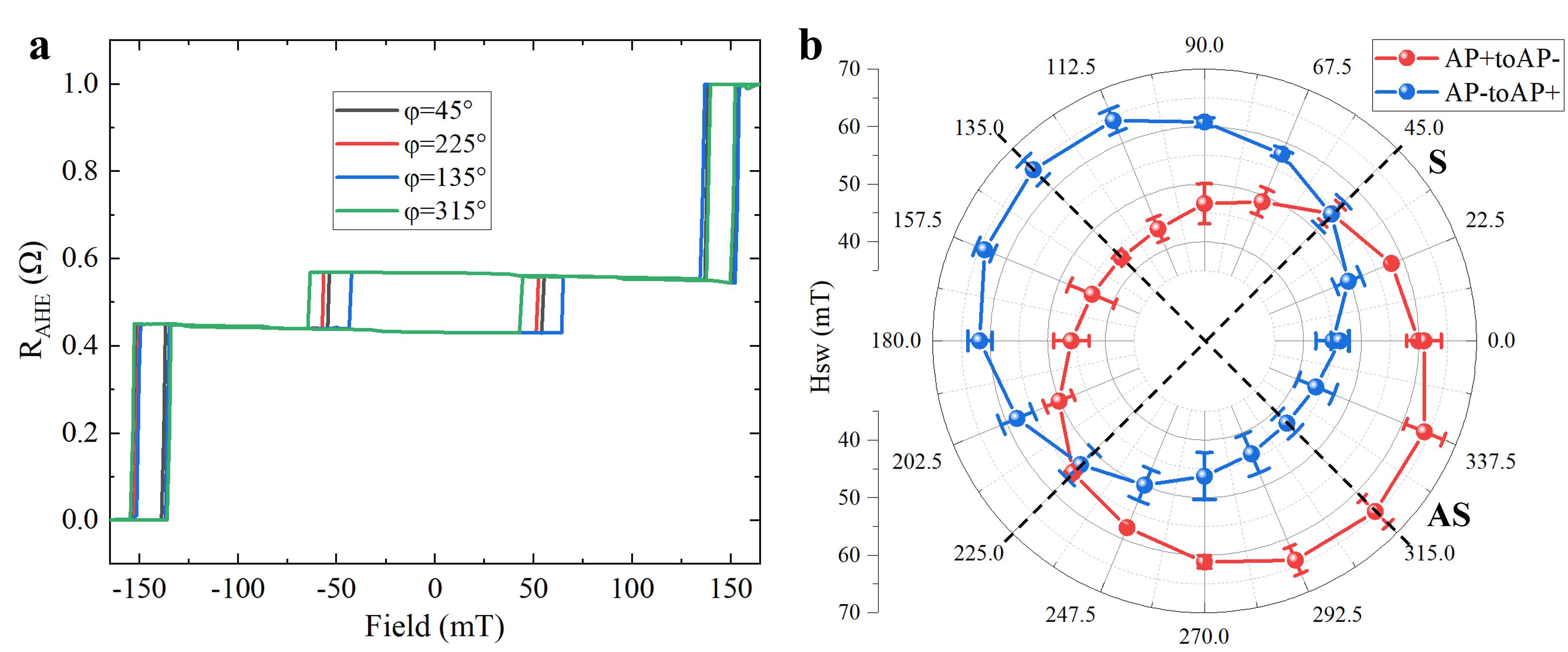


**Supplementary** **Figure S17. Characterization of the IL-DMI effect for S_3.** (a) Maximum and minimum AHE loop shifts of SFi S_3. Angle *φ* refers to the additional *H_IN_* direction. (b) Azimuthal angular dependence of the switching field during the DLS process of S_3.

When the magnetic moment difference between the upper and lower ferromagnetic layers is larger, the hysteresis loop shift in the DLS process of S_3 is slightly greater than that of S_2. This indicates that a larger uncompensated magnetic moment may lead to a greater hysteresis loop shift. Table S1 summarizes the IL-DMI asymmetric axis directions and effective hysteresis loop shifts for all the devices we measured. As shown in the table, all devices exhibit switching field shifts in the range of 1–3 mT during the SLS process, whereas the shifts exceed 20 mT during the DLS process. This clearly demonstrates that DLS significantly amplifies the hysteresis loop shift induced by IL-DMI. For S_2 and S_3, a larger uncompensated magnetic moment results in a slightly increased switching field shift. Additionally, the asymmetric axes associated with IL-DMI for all devices lie in the second quadrant, which may be attributed to slight variations in the oblique sputtering angle of the Ir target across different positions on the substrate. This table demonstrates that significantly uncompensated SFi is a better system for detecting IL-DMI.

Table S1 IL-DMI asymmetric axes and effective field shifts for all the devices

| Sample | Hall bar | Maximum switching field shift in SLS (mT) | Maximum switching field shift in DLS (mT) | *φ_AS_* | *φ_S_* |
| --- | --- | --- | --- | --- | --- |
| S_1 | 1 | 2.7 (±0.3) | \ | 145° | 55° |
|  | 2 | 2.12 (±0.6) | \ | 112.5° | 22.5° |
| S_2 | 1 | 1.23 (±0.35) | 22.2 (±3.4) | 123° | 33° |
|  | 2 | 1.74 (±0.31) | 22.8 (±4.2) | 145° | 55° |
|  | 3 | 1.55 (±0.39) | 21.7 (±4.7) | 135° | 45° |
|  | 4 | 1.91 (±0.24) | 23.3 (±3.3) | 123° | 33° |
| S_3 | 1 | 1.63 (±0.66) | 23.1 (±3.9) | 135° | 45° |
|  | 2 | 1.77 (±0.61) | 24.1 (±3.7) | 157.5° | 67.5° |
|  | 3 | 1.97 (±0.61) | 24.7 (±3.4) | 123° | 33° |

In all devices, the IL-DMI asymmetric axes are aligned along the second and fourth quadrants, while the symmetric axes are along the first and third quadrants (see Table S1). We speculate that this is caused by the tilted alignment between the Ir target and the substrate. The following figure S18 illustrates the relative configuration between the Ir target and the substrate (Side view and top view). The oblique sputtering of Ir atoms breaks the IP symmetry, thereby introducing the IL-DMI. However, the specific direction of asymmetry varies depending on the relative position between the Hall bar device and the target.


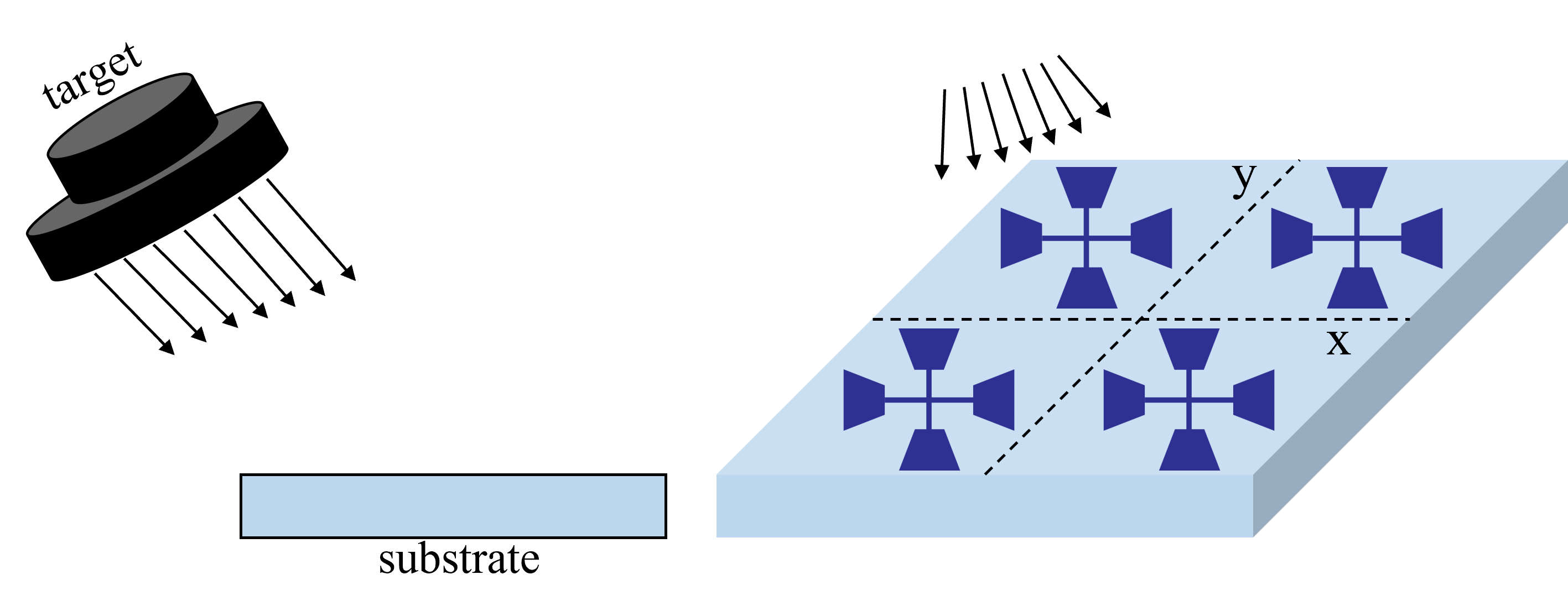


**Supplementary** **Figure S18. The relative configuration between the Ir target and the substrate (Side view and top view).**

**S10. The procedure for extracting the threshold switching current and complete analog-like SOT switching of S_3 and S_2 under different IP fields.**

The procedure for extracting the threshold switching current is as follows: First, the AHE curves under external magnetic fields and the resistance at zero current are used to determine the resistance values of the AP+ and AP- states, as shown in Fig. 3b and 3c of the main text (the horizontal dotted lines). The average of *R_AP+_* and *R_AP−_* is then taken to represent the resistance of the intermediate state. This intermediate resistance is used as a reference to define the threshold switching current—i.e., the SOT current at which the device resistance reaches this value. Figure S19 and S20 below are the SOT switching curves of the device under additional external magnetic fields. The horizontal dashed line indicates the intermediate resistance, while the two vertical dashed lines mark the threshold switching currents during the positive and negative current sweeps, respectively. Figures S19 and S20 present the measurement results for devices S_3 and S_2, respectively. The IP field amplitude applied in each case is indicated in the upper left corner. The threshold switching currents extracted from these results, along with those from Figures 3b and 3c in the main text, form the experimental data shown in Figures 3d and 3e.


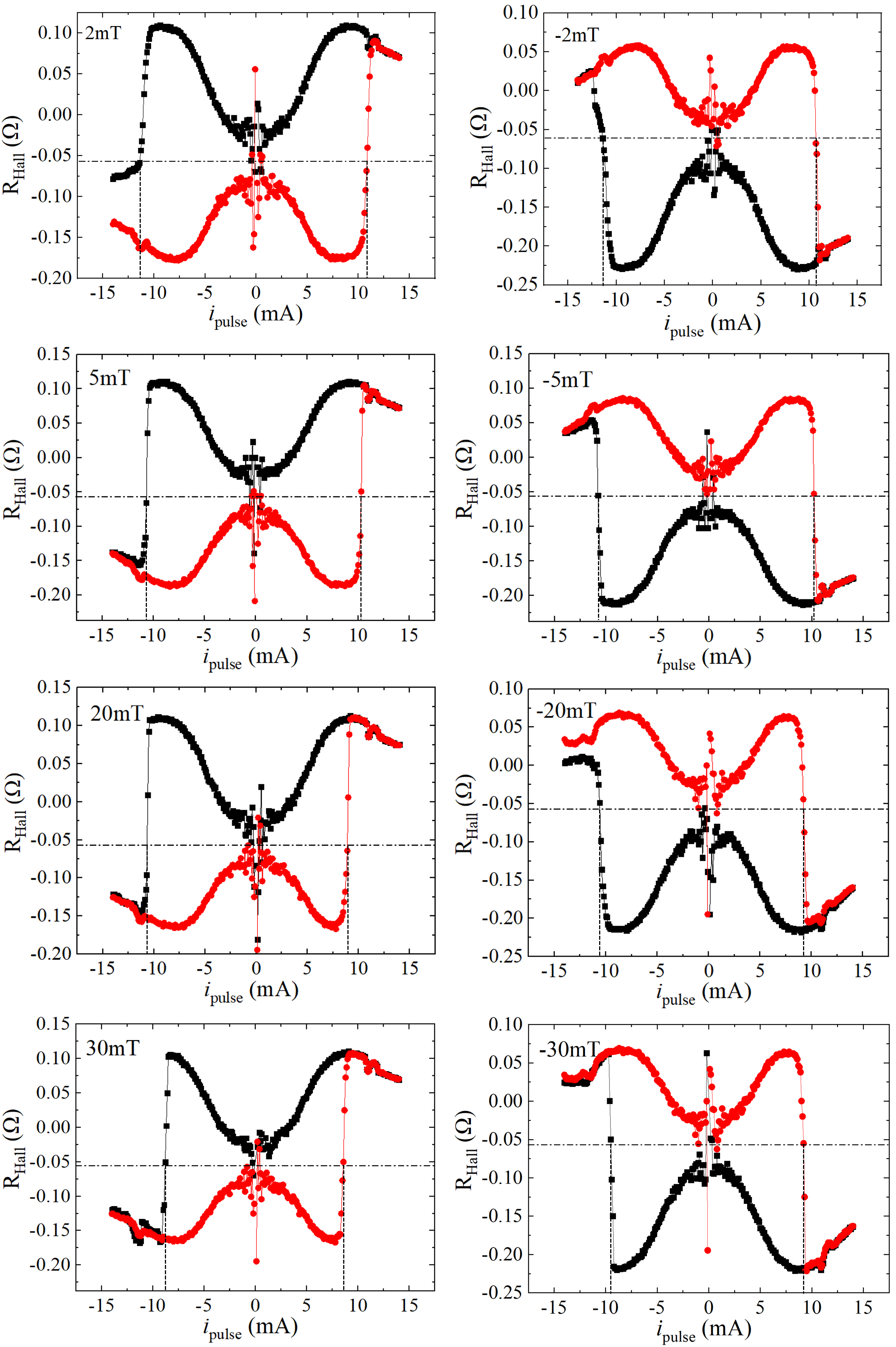


**Supplementary** **Figure S19. Analog-like SOT switching of S_3 under different IP fields.**


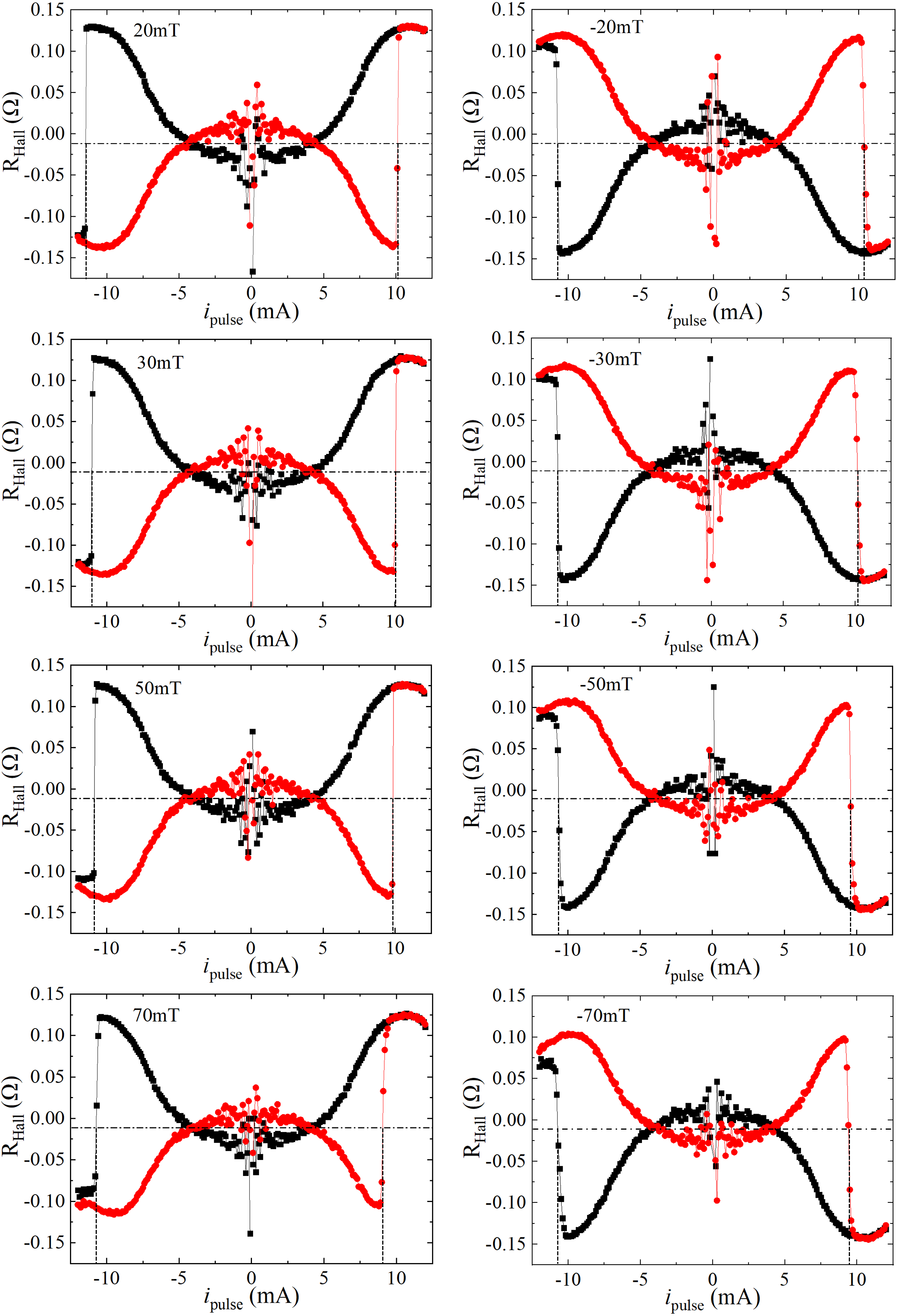


**Supplementary** **Figure S20. Analog-like SOT switching of S_2 under different IP fields.**

**S11. Detection of the intermediate resistance states of SFi S_1 during the SOT switching.**

The detection of the intermediate states of S_1 during the SOT switching is shown in this section. Figure S21a shows the different switching behaviors under different IP fields. It is basically consistent with the switching behavior of S_2 shown in Figure 3c in the main text. Figure S21b shows the asymmetric critical current distribution with different H_IP_ of S_1. During the SOT switching process, regardless of the direction of H_IP_, the critical current in the positive scan is always smaller than that in the negative scan. This is also consistent with the switching results of S_2 and S_3.


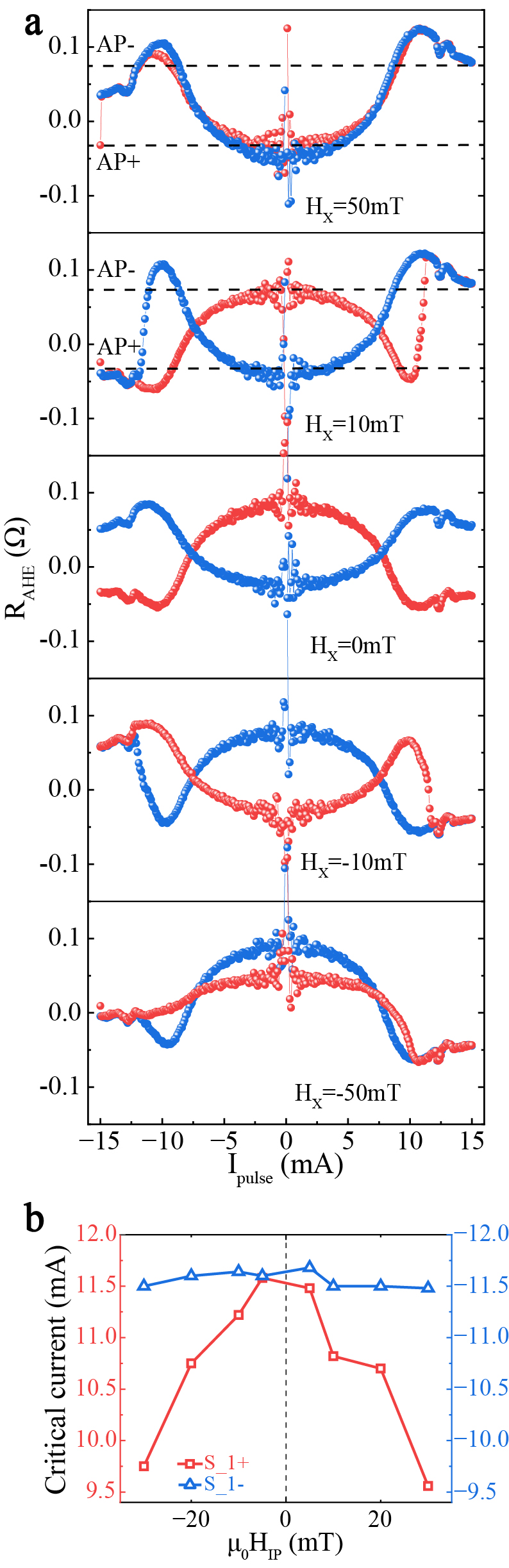


**Supplementary** **Figure S21**. **Detection of the intermediate resistance states of SFi S_1 during the SOT switching.** (a) R_AHE_ versus SOT pulse curves of S_1 under different IP fields. (b) Asymmetric critical current distribution with different *H_IP_* of S_1.

**S12. SOT-induced Kerr-imaging dynamics and size-scaling results of the Hall bar devices.**

Multi-domain states and domain-wall motion induced by SOT could also, in principle, lead to an analog-like evolution of the AHE signal. To examine this possibility, we applied SOT current to a 10-μm-wide Hall-bar device and directly visualized its switching dynamics. The Kerr microscopy results are presented in Fig. S22. Figure S22a shows the magnetization distribution under an in-plane field of 10 mT at different SOT current densities. Because the total magnetic thickness of our stack is ∼10 nm, the signals from the two ferromagnetic layers overlap in the Kerr image. When a current of –2×10¹¹ A/m² is applied, the Kerr contrast becomes darker; increasing the current further to –3×10¹¹ A/m² continues to deepen the contrast. Once the threshold current is reached (–4×10¹¹ A/m²), the Kerr contrast abruptly switches to a lighter color. Throughout this process, we do not observe evidence of multi-domain formation; instead, the magnetization of the Hall bar evolves uniformly under the combined influence of thermal effects and IL-DMI. For comparison, Fig. S22b displays the switching between AP+ and AP- states under an out-of-plane magnetic field. With these reference states, Fig. S22c plots the Kerr contrast at different SOT current densities, with the relative signal amplitudes of AP+ and AP- marked accordingly. The resulting SOT-driven switching curve closely matches the AHE measurements, indicating that the analog evolution arises from a gradual tilting of magnetization rather than multi-domain formation or domain wall motion.


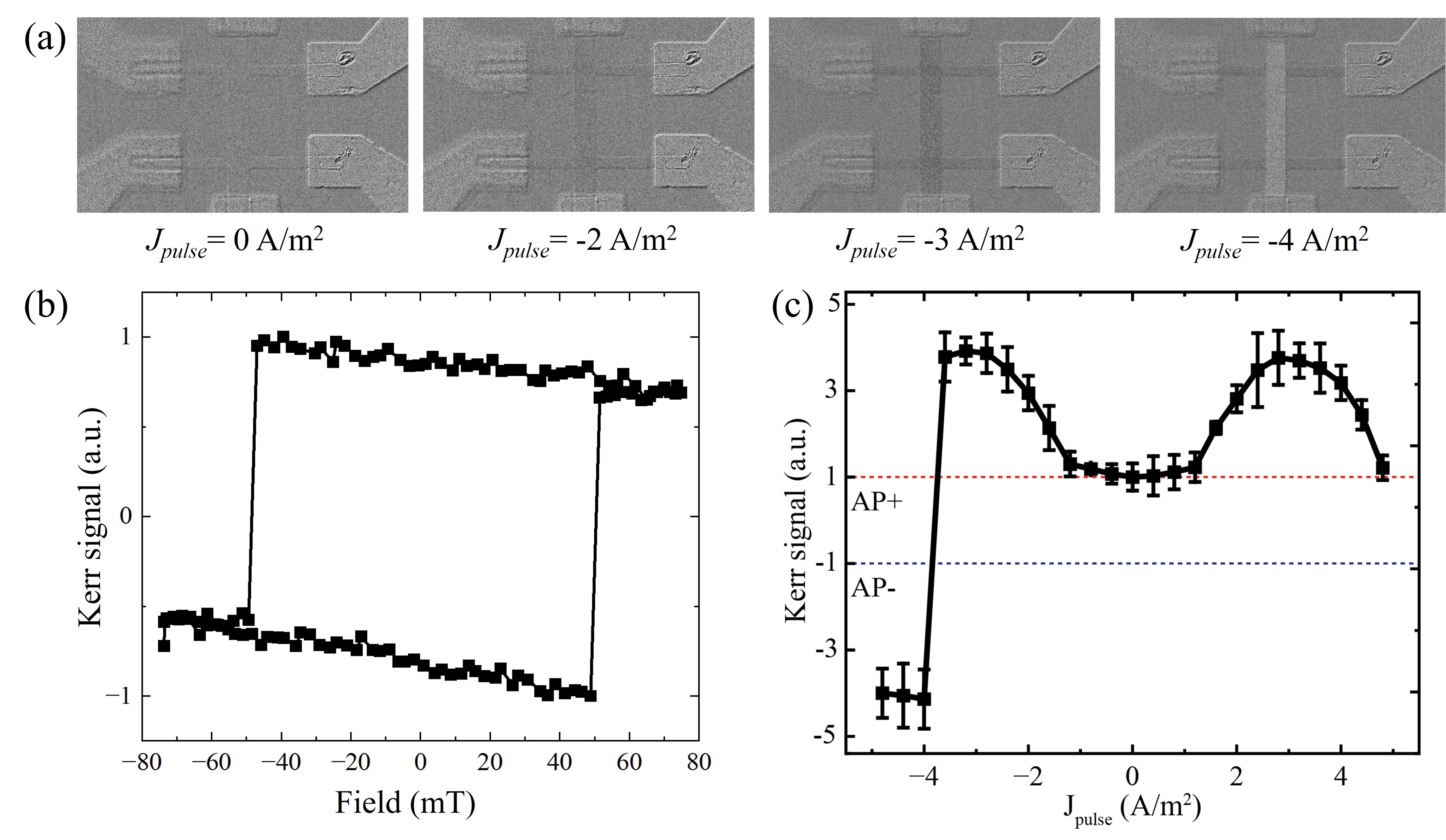


**Supplementary** **Figure S22**. (a) Kerr images of our Hall bar device under an in-plane field of 10 mT at different SOT current densities. (b) Kerr signal of the device under an out-of-plane magnetic field indicating the switching between AP+ and AP- states. (c) The Kerr signal evolution with varying SOT current densities.

In addition, your suggestion to compare the switching behavior between micrometer-scale and sub-200-nm devices was highly insightful for us. Although our current fabrication and characterization conditions do not yet allow Hall-bar measurements with 200 nm width, we systematically compared the analog-like switching behavior across all Hall-bar devices we have fabricated with different widths. Figure S23a presents the SOT-driven switching curves for Hall bars with widths of 20, 10, 4, and 2 μm. While little variation exists in threshold current density and the shape of the analog transition, all devices exhibit very similar analog-like switching characteristics. Accordingly, Figure S23b compares the maximum AHE resistance change for the four devices, which reflects the maximum degree of magnetization tilting. Interestingly, the maximum AHE contrast does not decrease as the device size shrinks; instead, it shows a slight increasing trend. This further confirms that the analog-like switching originates from a collective tilting of magnetization rather than domain-wall-mediated multi-domain evolution. By extension, sub-200-nm-scale devices are also expected to exhibit similar analog-like switching behavior.


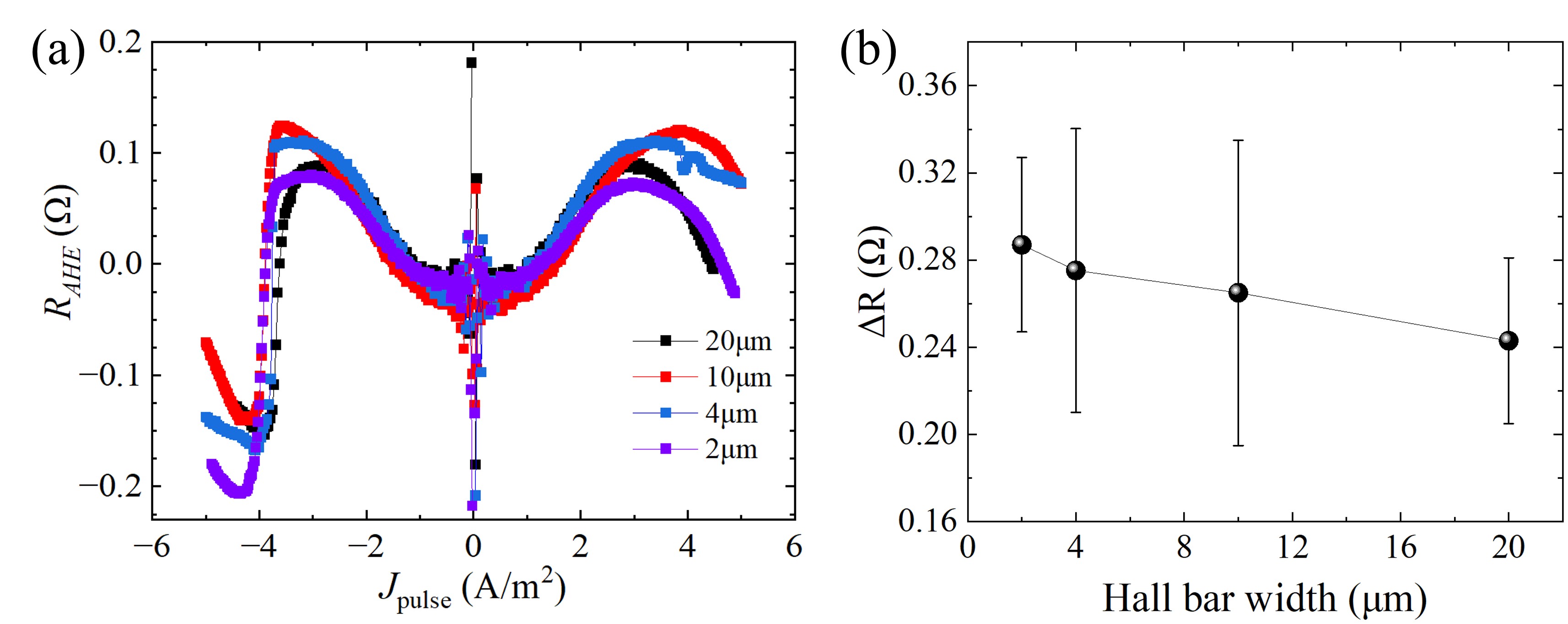


**Supplementary** **Figure S23**. (a) SOT-driven switching curves for Hall bars with widths of 20, 10, 4, and 2 μm. (b) The maximum AHE resistance change for the four devices.

In our present study, the layer-resolved reversal trajectories—including synchronized in-plane rotation during DLS and asymmetric tilting under SOT—are indeed supported primarily by micromagnetic simulations and AHE measurements. Direct layer-resolved measurements are not yet available in our current experimental setup. However, we provide two independent pieces of indirect experimental evidence that strongly constrain possible magnetization configurations:

1. Kerr-imaging dynamics and size-scaling results

The gradual continuous Kerr contrast evolution under SOT, with no observable multi-domain nucleation, indicates a coherent rotational mode in the FM layer. The behavior remains robust across device widths (20 to 2 μm), consistent with IL-DMI–stabilized collective tilting as predicted by simulation.

2. Detailed AHE measurements combined with the full reproduction of all key experimental results by micromagnetic simulations

Supplementary Section S8 provides detailed hysteresis-loop measurements under various fixed in-plane fields, further confirming the strongly enhanced IL-DMI effective field during the DLS process. We believe that the synchronized in-plane rotation during the simultaneous reversal of the two ferromagnetic layers can be reliably identified, as it represents the energetically favored switching pathway under antiferromagnetic interlayer coupling. The AHE measurements can deterministically capture the out-of-plane magnetization evolution of each FM layer. Building on this, our micromagnetic simulations accurately reproduce all key experimental observations, including SLS, DLS, analog-like SOT switching, and the asymmetric threshold-current distribution. The strong agreement between experiment and simulation supports the reliability of the inferred layer-resolved magnetization dynamics.

Together, these results do not fully resolve the individual magnetic layer states directly, but they do significantly narrow the configuration space, indicating that synchronized layer-dependent rotation is the most reliable mechanism. We will clarify this in the revised manuscript, explicitly distinguishing what is experimentally supported and what is inferred through simulation. In future work, we aim to adopt layer-resolved techniques, such as layer-resolved MOKE, XMCD-PEEM or magnon-mode spectroscopy to further directly visualize and verify the layer-specific trajectories.

**S13. Non-volatile multi-resistance-state SOT switching results under different pulse widths.**

It is worth noting that the analog-like SOT switching observed in our results (Figure 3) is different from the memristive SOT switching reported in previous studies. The memristive intermediate states formed in this process are non-volatile and depend on factors such as the maximum value of the scanning SOT pulse or the width of the SOT pulse. These intermediate states lie between AP+ and AP-, and their non-volatile nature makes them suitable for implementing memristors in SOT-based neuromorphic computing. In contrast, the intermediate states in the analog-like SOT switching of our devices (Figure 3) arise from the gradual tilting of the top and bottom layers towards perpendicular alignment. These states go beyond the AP+ or AP- states and are volatile. Based on analog-like SOT switching, we have designed and implemented the LIF model of neurons in a SNN, which is elaborated in the Spintronic LIF neuron implementation in SNN section.

To distinguish it, we also demonstrated the non-volatile memristive characteristics in our device by varying the SOT pulse width. Here, an extra IP field is applied along *x* direction to achieve the deterministic switching. During the SOT pulse scanning, the pulse widths are uniformly set to 50 μs, 25 μs, 5 μs, 1 μs, and 0.2 μs, respectively (figure S24). The AHE resistance is obtained through a steady-state detection method. When the pulse width was 50 μs, the device achieved a full switching and the resistance difference (0.08 Ω) before and after switching was close to the AHE resistance difference observed during magnetic field scanning. As the pulse width decreased, the resistance difference before and after switching gradually decreased, indicating partial switching of the device.


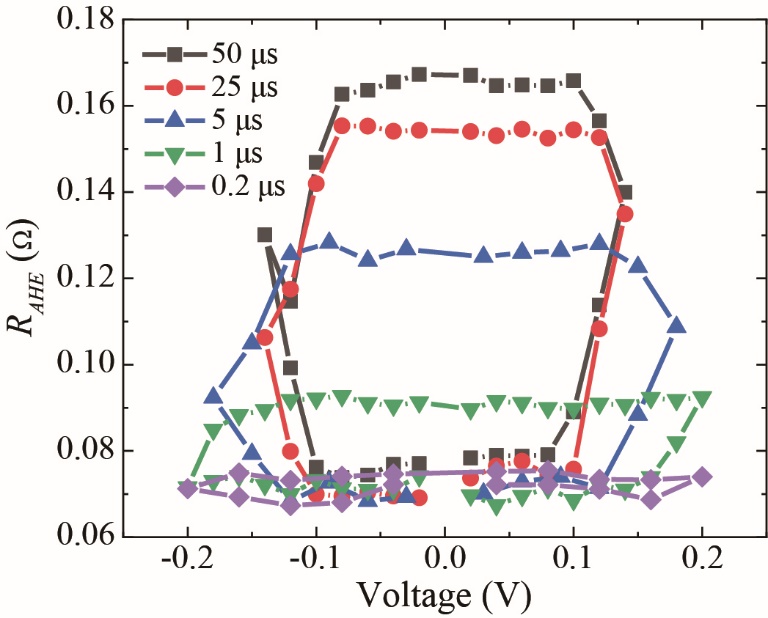


**Supplementary** **Figure S24**. **Characterization of the memristive switching behaviour in our SFi device by varying the SOT pulse width.**

**S14. Characterization of the effective IL-DMI field of SFi S_2 for field-free SOT switching.**

We further demonstrate the effective field by plotting the AHE resistance difference before and after switching as a function of magnetic field shown in Figure S25. The result indicate that the effective fields produced by IL-DMI at *φ*=0° and *φ*=90° are 0.2 mT and -0.1mT, respectively.


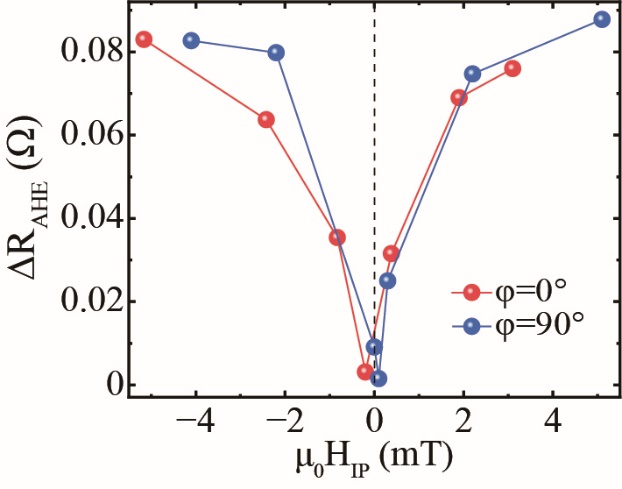


**Supplementary** **Figure S25**. **The dependence of the absolute value of the AHE resistance change with IP field at *φ*=0° and *φ*=90°.**

**S15. Speculation on the reasons for the relatively small IL-DMI effective field measured during field-free SOT switching.**

As shown in Fig. 4b and 4c of the main text, the steady-state switching results based on SOT indicate that the IL-DMI provides an effective field for SOT-induced switching. The magnitude and direction of this effective field vary with the azimuthal angle of the applied current. In this case, the effective field is 0.2 mT along φ=0°, which is too small to assist SOT in achieving fully deterministic switching. In this SFi device, the IL-DMI effective field is 1.03 mT along φ=0° in asymmetric hysteresis loop switching measurement, which is also relatively small. Thus, the IL-DMI in our devices is not strong. We believe there are two main reasons for the absence of remarkable field-free switching in our devices:

1. For the basic antiparallel magnetization configuration, the effective in-plane field generated by the IL-DMI in our device reaches only 1.03 mT, as determined from asymmetric hysteresis loop measurements. This effective field is relatively small for enabling deterministic switching via SOT with in-plane field assistance. However, during the DLS process, the effective field can be significantly amplified up to 22.2 mT through the synergetic effect of the uncompensated magnetic moments and IL-DMI. The reason for the difference between the two lies in the fact that the former contributes indirectly as an IP field, while the latter acts as an OOP field that directly combines with or competes against the vertically scanned magnetic field.
2. There is a discrepancy between the 1.03 mT effective field extracted from asymmetric hysteresis loop measurements and the 0.2 mT field inferred from field-free SOT switching experiments. We attribute this difference to thermal effects. The steady-state measurement method merely ensures that the final obtained AHE resistance excludes the influence of thermal effects. However, thermal effects are always present and cannot be neglected during SOT switching. IL-DMI, similar to RKKY exchange coupling, arises from indirect exchange interactions mediated by conduction electron spins interacting with localized magnetic moments. Recent studies have shown that RKKY coupling strength decreases with Joule heating induced by electric current. The RKKY effective field decreases linearly with the square of the current density [10]. Given their similar physical origins, we speculate that Joule heating during the SOT switching process weakens the IL-DMI effect, thereby reducing the effective switching field observed in our experiments.

These two factors collectively contribute to the absence of distinct field-free switching. Field-free SOT switching is not the main focus of our work. To address the challenge of field-free SOT switching, for the first issue, researchers may consider to increase the oblique sputtering angle of the Ir target to enhance the IL-DMI strength. In our current devices, IL-DMI arises naturally from the slight angular offset between the target and the substrate. However, a recent study has shown that a larger oblique sputtering angle can strengthen the IL-DMI [11]. To address the second issue, researchers may investigate the temperature dependence of IL-DMI by measuring the effective IL-DMI field under variable temperature conditions to validate our hypothesis.

**S16. Micromagnetic simulation results of magnetization dynamics during the binary-state SOT switching.**

Here, we used micromagnetic simulation to prove that IL-DMI will cause the magnetic moments of the upper and lower layers to be aligned perpendicularly during SOT applying. Micromagnetic simulations were carried out by solving numerically the LLG equation augmented with damping-like SOT that considers the contribution of the current flowing through the heavy metal layer. In our micromagnetic simulations, we constructed a three-layer synthetic ferrimagnet structure with a mesh of 100 × 50 × 3 cells and a cell size of 5 × 5 × 1 nm, corresponding to a Hall-bar geometry of 500 nm in length and 250 nm in width. The damping constant was set to 0.3. Here, we additionally provide the micromagnetic simulation procedures for reproducing the two-step and three-step switching behavior. Theoretically, in a synthetic antiferromagnet, the switching between AP^+^ and AP^-^ is determined by the magnetic anisotropy and the thickness difference between the two ferromagnetic layers: $|\mu_{0}H_{AP^{+}\leftrightarrow AP^{-}}|=\frac{2K}{M_{S}\Delta t}$, while the switching from P to AP⁺ is determined by the RKKY coupling strength together with the thickness and saturation magnetization of the thinner ferromagnetic layer: $|\mu_{0}H_{P^{+}\leftrightarrow AP^{+}}|=\frac{|J_{E}|}{t_{2}M_{2}}$ [2]. When the difference between these two characteristic switching fields is large, the hysteresis loop exhibits a three-step switching; when the difference is small, a two-step switching behavior appears instead. For the case where the saturation magnetization of the top and bottom ferromagnetic layers differs, the micromagnetic simulation results also reproduce a similar trend. Since MuMax employs a uniform simulation cell size, we are unable to vary the individual layer thicknesses to directly reflect the experimentally observed two-step and three-step switching behaviors. Therefore, we equivalently represented the change in thickness through an adjustment of the saturation magnetization of bottom FM layer. Specifically, for the 0.9-nm-thick bottom ferromagnet, we set the saturation magnetization to 0.62 MA/m. For the 0.8-nm bottom layer, we scaled the saturation magnetization proportionally, i.e., 0.62/0.9×0.8=0.55 MA/m. All other simulation parameters were kept identical. The hysteresis loop comparison shown below demonstrates that when the saturation magnetization is equivalently modified, the switching behavior transitions from two-step to three-step, consistent with experimental observations.


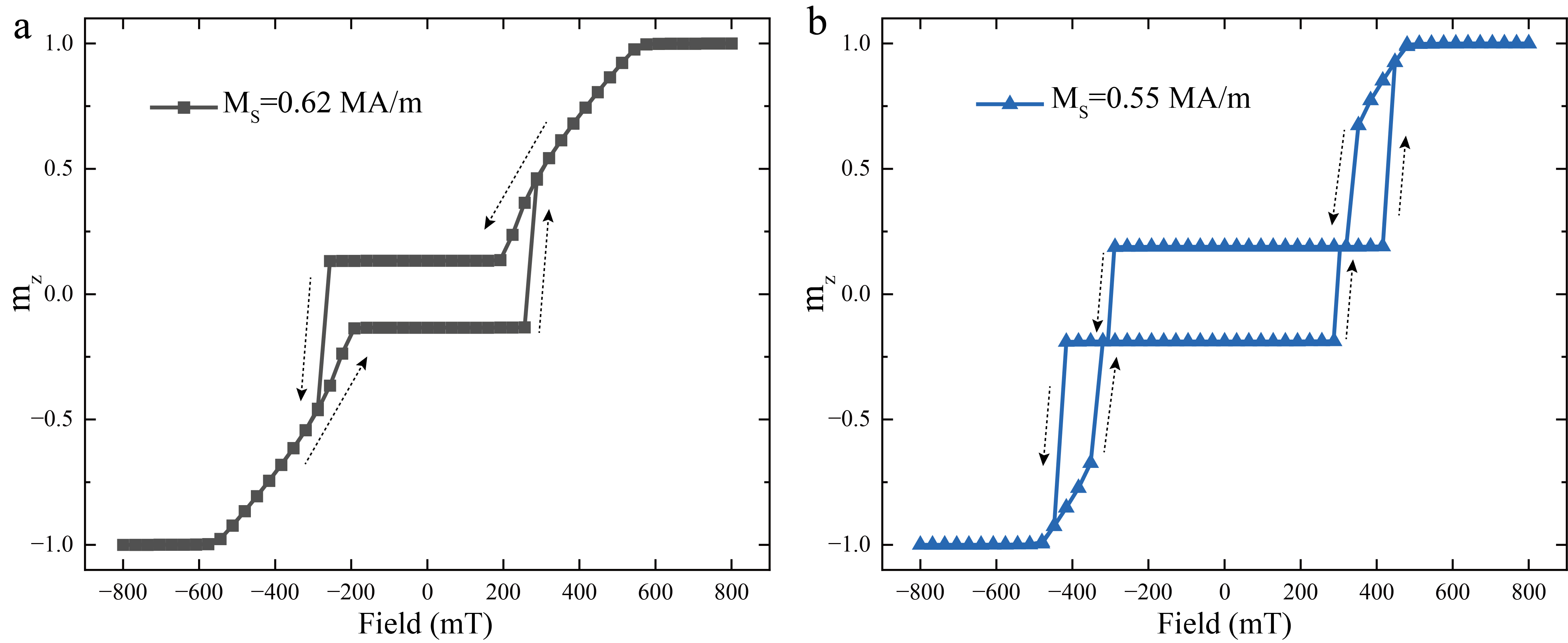


**Supplementary** **Figure S26**. **Hysteresis loops of Ta/Pt/Co/Ir/Co/Pt with different bottom Co saturation magnetization.**

We included the contribution of the interlayer DMI as an effective field in the micromagnetic simulator Mumax3 [12] with the energy form as $E_{DM}=D\cdot\left( S_{1}\times S_{2} \right)$. Since MuMax3 does not natively support IL-DMI, we implemented IL-DMI by introducing customized effective fields and energy densities, as described below:

DDZ1 := Const( (Dinter) / (cellsize*tsp*Ms1))

DDZ2 := Const( (Dinter) / (cellsize*tsp*Ms2))

up1 := Mul(DDZ1,cross(u,Shifted(m,0,0,2)))

down1 := Mul(DDZ2,cross(Shifted(m,0,0,-2),u))

Bidc := Add(up1,down1)

AddFieldTerm(Bidc)

addEdensTerm(Mul(Const(-0.5),Dot(Bidc,M_full)))

The parameters of ferromagnetic material for the simulations are chosen to be A_ex_ = 19 pJ/m (exchange stiffness) [13]; M_S_ = 0.85 MA/m (upper layer) and 0.55 MA/m (lower layer) (saturation magnetization); K_u_ = 0.26 MJ/m^3^ and 0.52 MJ/m^3^ (uniaxial anisotropy). The interlayer DMI and RKKY coupling strength are set as D_1,2_ = 0.12 mJ/m^2^ and J_RKKY_ = 0.3 mJ/m^2^, respectively. In the simulations, the RKKY coupling and IL-DMI strength were intentionally increased within a reasonable range to account for the size discrepancy between the simulated and actual devices. The simulated Hall bar has dimensions of 500 nm × 250 nm, whereas the fabricated devices are at the micrometer scale (50 μm in length and 2–10 μm in width). Although the saturation magnetization and magnetic anisotropy parameters are directly extracted from our experiments, the difference in physical dimensions leads to a fundamental distinction in switching field. The much smaller device size behaves closer to a Stoner–Wohlfarth single-domain regime. As a consequence, the simulated switching field is naturally larger than the experimental value, and therefore the RKKY and IL-DMI strengths were scaled accordingly to maintain realistic switching trends. A typical heavy metal spin Hall angle of 0.13 is used [14][15]. The spin polarization is set as [0 -1 0] when the current is along [1 0 0]. Simulations confirm that reversing the sign of the spin Hall angle or the direction of the IL-DMI ***D*** vector can both lead to an inversion in the asymmetry of the threshold current distribution. Figure S27 shows the inversed SOT switching result when the spin Hall angle is reversed.


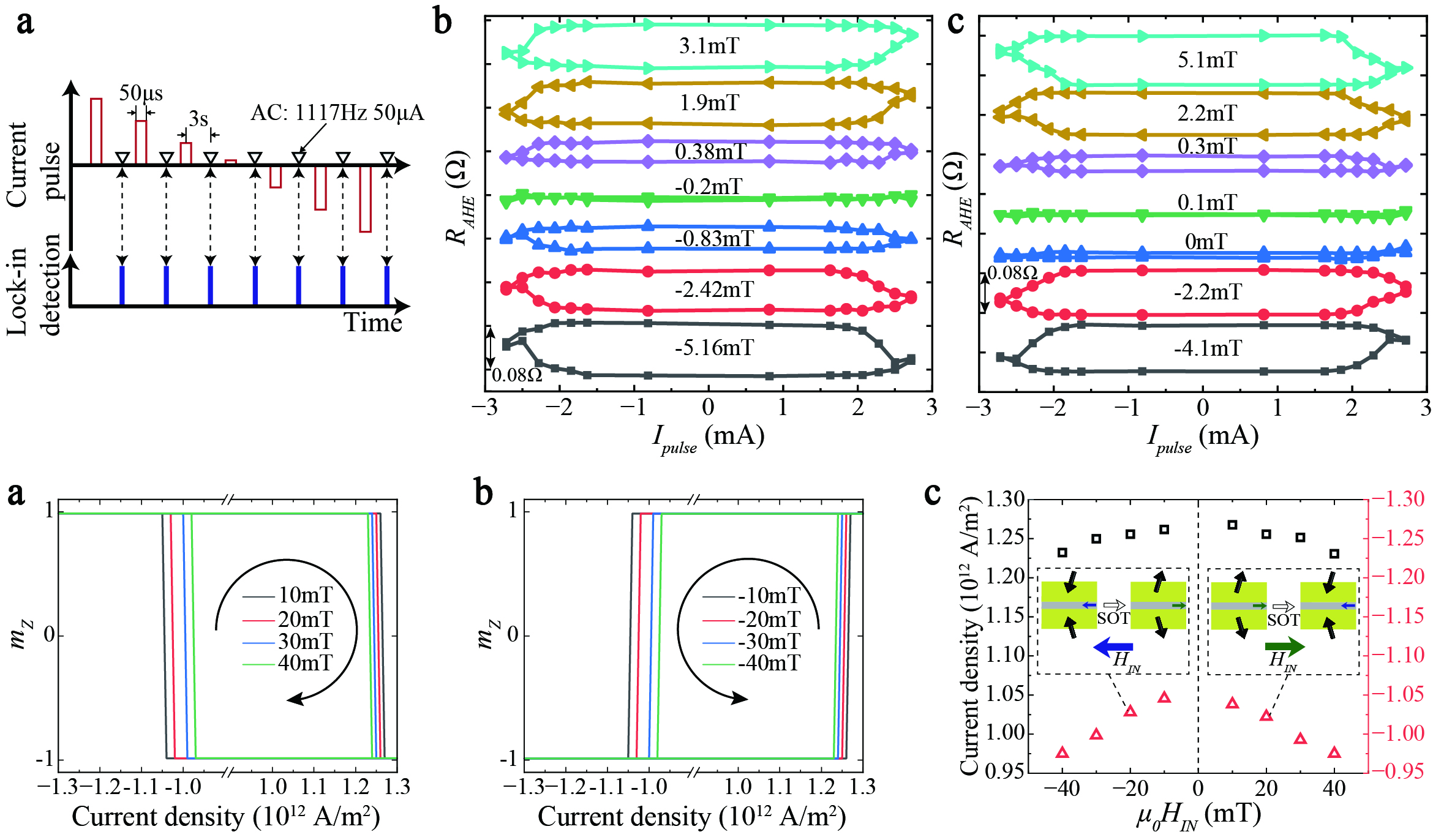


**Supplementary** **Figure S27**. **Micromagnetic simulation of asymmetric critical switching current distribution with reversed spin Hall angle.** Magnetization switching of the SFi versus SOT current density with the assistance of positive (a) and negative (b) IP fields. (c) Asymmetric critical current distribution with different IP fields.

The resistance of the device in the AP state can be expressed as $R_{AHE}=\left| R_{s}^{b}M_{z}^{b} \right|-\left| R_{s}^{u}M_{z}^{u} \right|$, where $R_{s}^{b}$ and $R_{s}^{u}$ are the AHE coefficients of the bottom and top layers, respectively, $M_{z}^{b}$ and $M_{z}^{u}$ are the perpendicular magnetization of the bottom and top layers, respectively. It is due to this gradually perpendicular alignment that the bottom layer magnetic moment $M_{z}^{b}$ gradually decreases, and the overall AHE signal gradually increases, which leads to the analog-like behavior of the AHE resistance before the SOT switching. Figure S28a shows the SOT switching dynamics of SFi with different IL-DMI magnitudes. The current is applied to the lower layer from 0.5 ns to 1.5 ns. Without IL-DMI, the magnetic moments of the upper and lower layers will simultaneously rotate to IP and align antiparallelly. With IL-DMI, the magnetic moment of the upper layer has not changed significantly. The m_top with IL-DMI of 1.5, 0, -1.5 μJ/m^2^ are basically superimposed. However, depending on the sign of IL-DMI, the lower layer magnetic moment will have a clear positive or negative vertical component. Figure S28b shows that under the action of IL-DMI, regardless of whether the SOT current reaches the critical value (3.2×10^13^ A/m^2^), the magnetic moments of the upper and lower layers will gradually be aligned from antiparallel to perpendicular configuration during the current applying with the increase of IL-DMI.


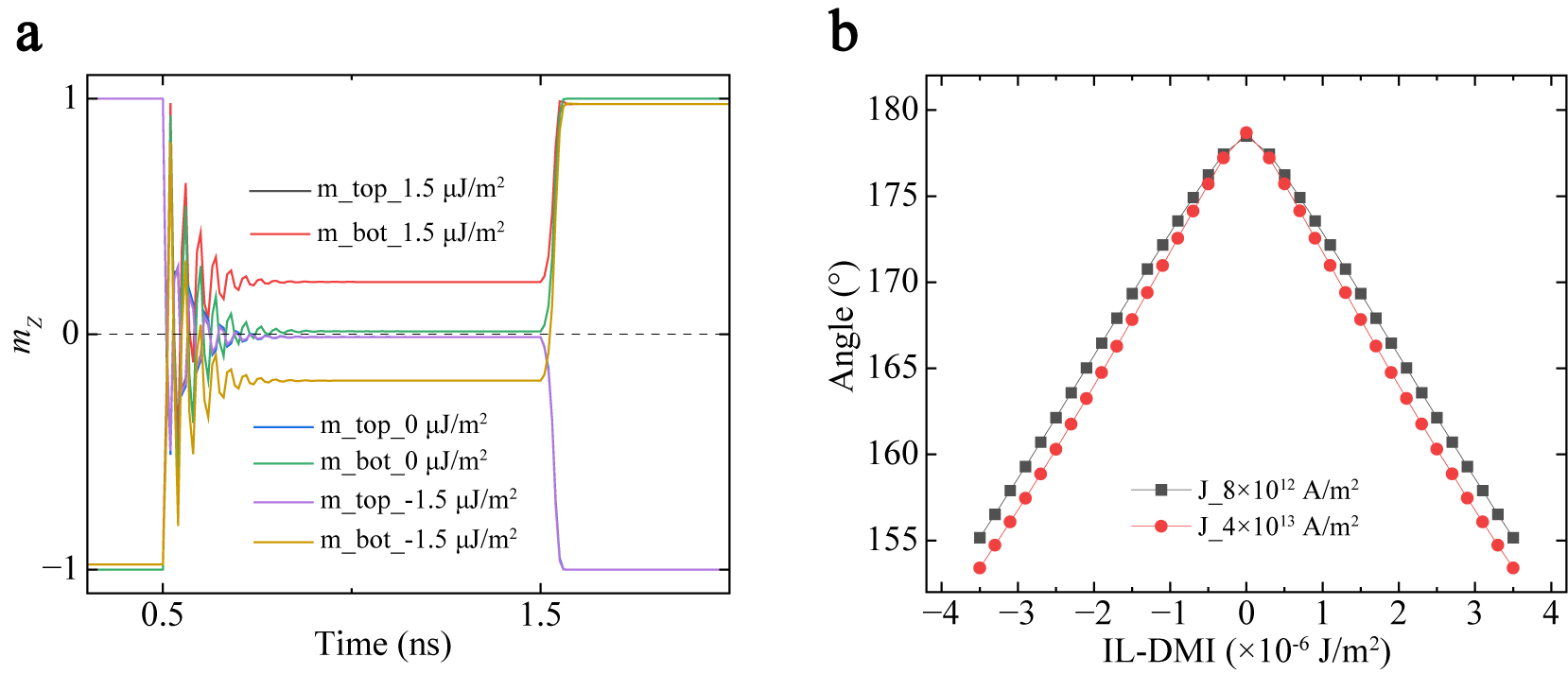


**Supplementary** **Figure S28**. **SOT switching dynamics and arrangement of upper and lower magnetic moments of SFi with IL-DMI.** (a) SOT switching dynamics of SFi with different IL-DMI magnitudes. The SOT current starts at 0.5 ns and stops at 1.5 ns. The current is applied to the lower layer. Without IL-DMI, during the SOT action, the magnetic moments of the upper and lower layers will simultaneously rotate to IP and align antiparallelly. The magnetic moment of the top layer will have a small downward component due to the influence of the magnetic field. With IL-DMI, the magnetic moment of the upper layer has not changed significantly. The m_top with IL-DMI of 1.5, 0, -1.5 μJ/m^2^ are superimposed. However, depending on the sign of IL-DMI, the lower layer magnetic moment will have a clear vertical upward or downward component. IL-DMI will cause the magnetic moments of the upper and lower layers to align perpendicularly during the SOT current applying. (b) The arrangement of upper and lower magnetic moments during SOT applying with IL-DMI. Regardless of whether the current reaches the threshold, as IL-DMI increases, the upper and lower magnetic moments will gradually transform from antiparallel to perpendicular alignment.

**S17. Micromagnetic simulation results of the analog-like SOT switching.**

Regarding Fig. 5 (a)–(c), we reproduced the experimental results shown in Fig. 3 using micromagnetic simulations to generate these results. Here, we provide a detailed description of the simulation process. First, based on the results of steady-state SOT simulations, we further incorporated thermal effects. By simulating the temperature variation of the device under different current densities, we accounted for the current-induced changes in saturation magnetization and magnetic anisotropy, and simulated the dynamic magnetization configurations of the top and bottom layers during current application. See the following section for more details on thermal effects. Due to the fact that both magnetic anisotropy and saturation magnetization dynamically change with the applied current, the simulation becomes significantly more complex. In the analog-like SOT switching simulation, the bottom layer magnetization, which experiences stronger effects from SOT, thermal excitation, and IL-DMI, exhibits a more pronounced in-plane tilt. Figure 5b shows the simulated SOT switching behavior of device S_3, in which the magnetization configurations are accurately generated via micromagnetic simulations.

Figure 5c in the main text is derived from the simulation shown in Figure 5b. Notably, both the experimental curve in Figure 3 and the simulation result in Figure 5b naturally exhibit a leaky-integrate-and-fire (LIF) behavior, similar to that of a biological neuron: the device resistance shows an integration-like increase with current density, returns to the antiparallel low-resistance state after the current is removed, and switches once the current exceeds a threshold. Furthermore, we applied a fixed threshold current density of 5×10^11^ A/m^2^ with a pulse width of 0.1 ns. With repeated pulse injection, thermal effects gradually accumulate, which in the simulation manifests as a reduction in saturation magnetization and anisotropy. Once the accumulated heating reaches a critical level, magnetization switching occurs.

Based on the above micromagnetic simulation model, we also include the effect of thermal assistance on the switching of the FM layer and the impact of thermal effects on SFi relaxation after the current is removed [10][16]. Given that the middle layer is Ir, we also consider the spin current generated by Ir acting on the upper FM layer. Figure S29a and b show two typical dynamic behaviors of the magnetic moments of the upper and lower layers when the current is below or above the critical value, respectively. Given that the analog-like SOT switching is obtained through transient measurements, in the micromagnetic simulation, we focus on the dynamic behavior of the magnetic moments during the application of SOT current to the device. Figure S29c shows the distribution of magnetic moments of the top and bottom layers during current application at different current densities. The AHE resistance of the SFi is calculated based on $R_{AHE}=R_{s}^{b}M_{z}^{b}+R_{s}^{u}M_{z}^{u}$. $R_{s}^{b}$ and $R_{s}^{u}$ are extracted from experimental data, with values of 0.1795 and 0.65, respectively.


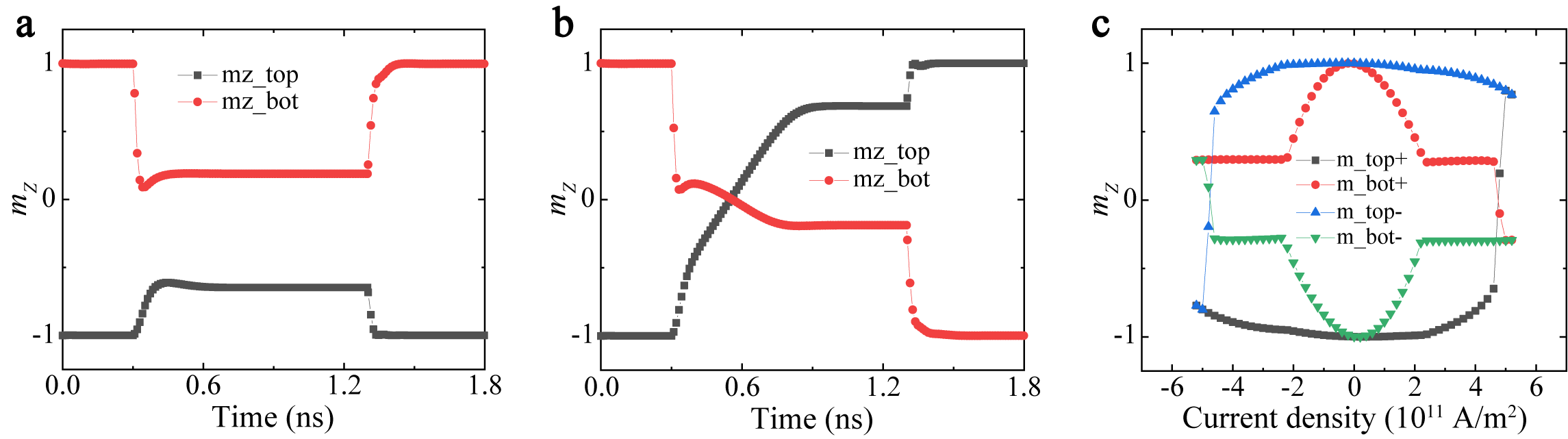


**Supplementary** **Figure S29**. **Micromagnetic simulation of the analog-like SOT switching.** (a) A typical dynamic behavior of the magnetic moments of the upper and lower layers when the current does not reach the critical value. The *m_Z_* of the top and bottom layers decrease to different degrees. (b) A typical dynamic behavior of the magnetic moments of the upper and lower layers when the current reaches the critical value. The *m_Z_* of the top and bottom layers switch during current application. c, The distribution of magnetic moments of the top and bottom layers during current application at different current densities.

**S18. Quantitative calculation and simulation of the thermal contribution in the analog-like SOT switching process.**

We first measure the variation of the device’s AHE resistance as a function of current under different applied OOP magnetic fields, as shown in the figure S30. Under the application of a 250 mT OOP magnetic field and in the absence of any external magnetic field, the device ultimately reaches a similar magnetization state due to the combined effects of thermal activation and IL-DMI. This indicates that the thermal effect and IL-DMI is significant enough to overcome the Zeeman field effect.


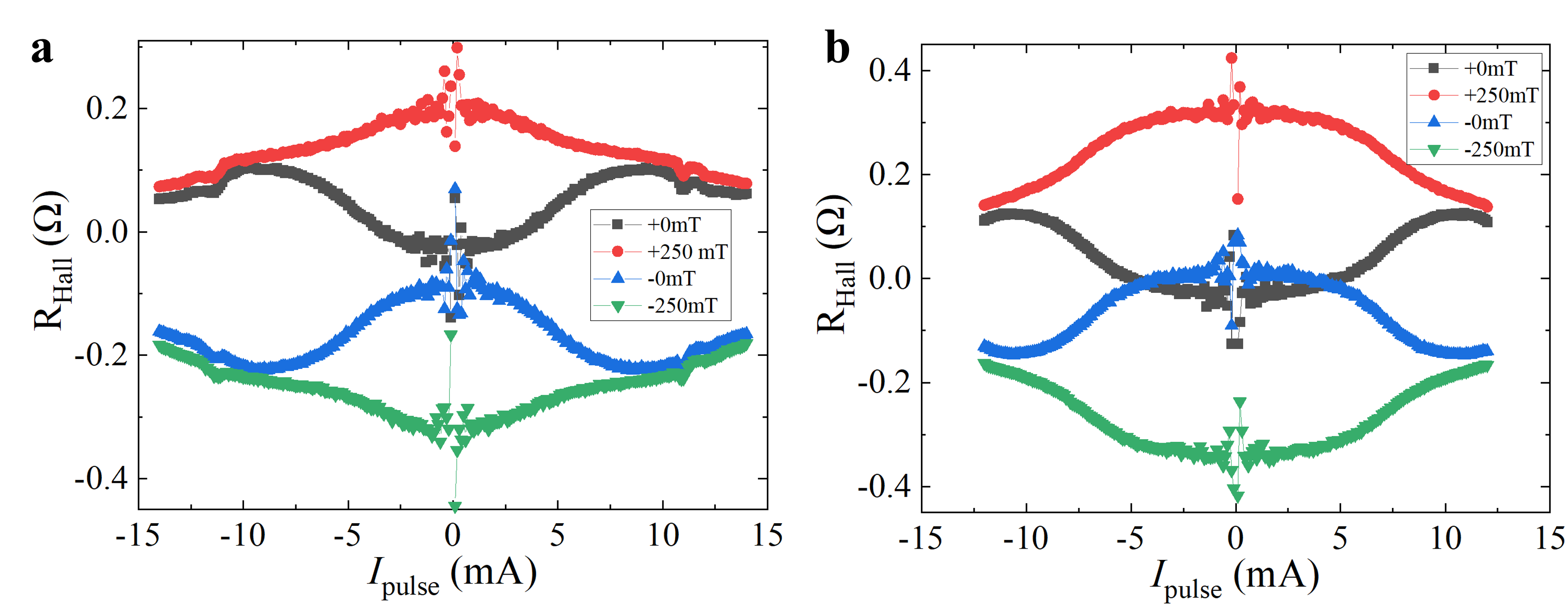


**Supplementary** **Figure S30. The variation of the device’s AHE resistance as a function of current under different applied OOP magnetic fields.** (a) and (b) indicate the results of device S_3 and S_2, respectively.

Due to experimental limitations, we are unable to precisely measure the temperature changes of the device during the SOT current sweeps. Here, we quantify the contribution of the thermal effect through simulations. The temperature change can be described as the following equations, referring to the thermally assisted magnetic recording model [17][18]:

$$C_{v}\frac{\partial T}{\partial t}=\frac{\partial}{\partial t}(Q_{in}+Q_{out})=V\times I-C_{v}\frac{T-T_{set}}{\omega}$$

where $C_{v}$ is the heat capacity, $\omega$ is the relaxation time. In the absence of external heating sources, the thermal relaxation time of such devices is typically on the order of microseconds. Here, we set $\omega$ =1.8 μs in our model[16][19]. *V* is the applied voltage, *I* is the applied current and $T_{set}$ is the stage temperature. The heat capacity is calculated based on the density, volume, and specific heat capacity of each layer in the Ta(30)/Pt(30)/Co(t)/Ir(14.5)/Co(10)/Cu(10)/Ta(30) stack (*C_v_ = sum(C_i_×m_i_), m_i_ = ρ_i_×V_i_*). The densities of Ta, Pt, Co, Ir, and Cu are 16650, 21450, 8900, 22560, and 8960 kg/m³, respectively. Their corresponding specific heat capacities are 140, 133, 420, 130, and 385 J/kg·K. Simultaneously taking into account heat dissipation and cooling after the current is removed [20],

$$T(t)=T_{0}+ V\times I\cdot R_{th}(1-e^{-t/\tau})$$

$$T(t)=T_{0}+(T_{peak}-T_{0}) \cdot e^{-(t-t_{p})/\tau}$$

where $T_{0}$ is the room temperature, $R_{th}$ is the thermal resistance and $\tau$ is the characteristic cooling time. Finally, we obtained the peak temperature of the device under different current amplitudes, as shown in the figure S31 below. The inset illustrates the temporal temperature evolution when a current of 10 mA is applied. The magnitude of the temperature rise is consistent with existing experimental results [16][21].


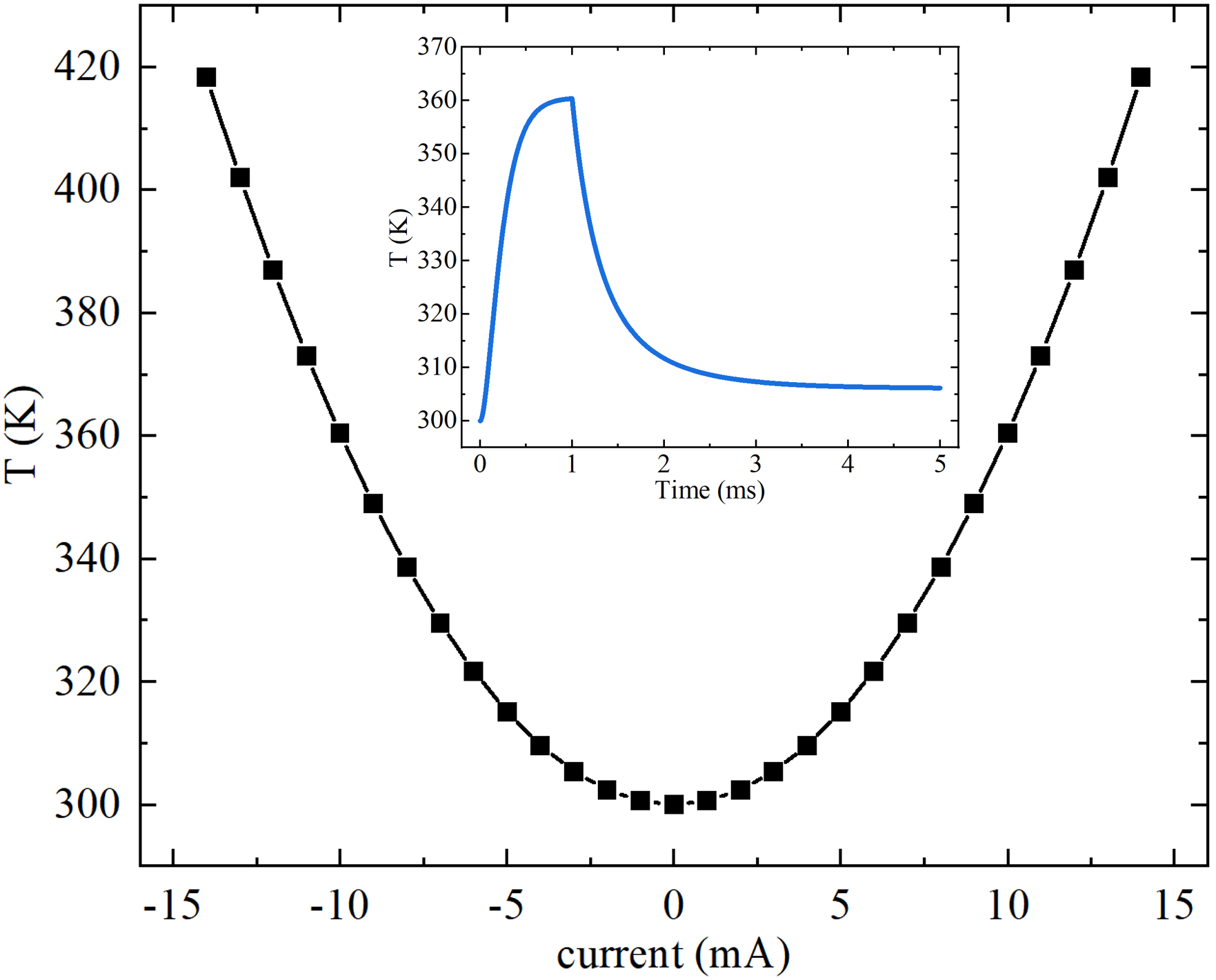


**Supplementary** **Figure S31. The peak temperature of the device under different current amplitudes. The inset shows the temperature evolution when a current of 10 mA is applied.**

Furthermore, we take into account the temperature dependence of the device's saturation magnetization $M_{S}$ and perpendicular magnetic anisotropy *K*. *M_S_* dependence with temperature *T* can be depicted with Bloch’s law [22] as

$$M_{S}(T)=M_{S}(0)\left[ 1-\left( \frac{T}{T_{C}} \right)^{x} \right]$$

where $T_{C}$ is the Curie temperature of Co and *x* is extracted from previous experiments [18]. The saturation magnetization (*Ms*) of the bottom ferromagnetic layer at T = 300 K is taken as 0.55 MA/m, obtained from SQUID measurements (figure S8). $M_{S}(0)$ is calculated to be 0.634 MA/m. The thermal effect corresponds to the variation of $M_{S}$ and interfacial anisotropy $K_{i}$. The variation of $K_{i}$ with temperature *T* can be described by [23]:

$$K_{i}(T)=K_{i}(0)\left[ 1-d_{th}\frac{T}{T_{C}} \right]\left[ \frac{M_{S}(T)}{M_{S}(0)} \right]^{3}$$

where $d_{th}$ is a dimensionless parameter. The anisotropy constant $K$ at T=300K is set to 0.52 MJ/m³, extracted from the IP AHE curve of the fabricated reference device Ta(30)/Pt(30)/Co(9)/Ir(14.5). The calculation method for *H_K_*​ is derived as follows. The equilibrium condition for the magnetic moment vector ***m*** is given by the torque balance equation[24]:

$$\vec{\tau}_{tot}=\vec{y}\cdot\left( \vec{\tau}_{SOT}+\vec{\tau}_{ext}+\vec{\tau}_{an} \right)=\tau_{SOT}^{0}+H_{ext}sin(\theta-\beta)-H_{K}sin\theta cos\theta=0$$

Here, $\vec{\tau}_{SOT}=\hbar\frac{J_{S}}{2eM_{S}t}\cdot\left( \boldsymbol{m}\times\left( \boldsymbol{\sigma}\times\boldsymbol{m} \right) \right)$ is the SOT torque, $\vec{\tau}_{ext}$​ is the torque induced by the external magnetic field, ​$\vec{\tau}_{an}$ is the torque resulting from the PMA field $H_{K}$​, $J_{S}$ is the spin current density, *e* is the elementary charge, *t* is the ferromagnetic layer thickness, ℏ is the reduced Planck constant, angle *θ* is the deviation of the magnetization vector from the z-axis and can be extracted via the Hall resistance as $sin\theta=R_{xy}/R_{A}$, and $\beta$ is the angle between the external magnetic field and the x-axis. When an in-plane field is applied along the x-axis and the current is sufficiently small, $\vec{\tau}_{SOT}$​ can be considered negligible and β=0. Substituting these conditions into the equilibrium equation yields the following expression for $H_{K}$:

$$R_{xy}/R_{A}=sin[arccos(H_{x}/H_{K})]$$

Figure S32 below shows the AHE curve of the reference device under in-plane field. The inset presents a magnified view near zero field, with the red line representing the corresponding fitted curve, yielding a value of $H_{K}=$0.945 T. Then the anisotropy constant calculated by $K=\frac{1}{2}\mu_{0}M_{S}H_{K}=$0.52 MJ/m³. $K_{i}(300)$ is determined to be 4.68×10^-4^ J/m^2^.


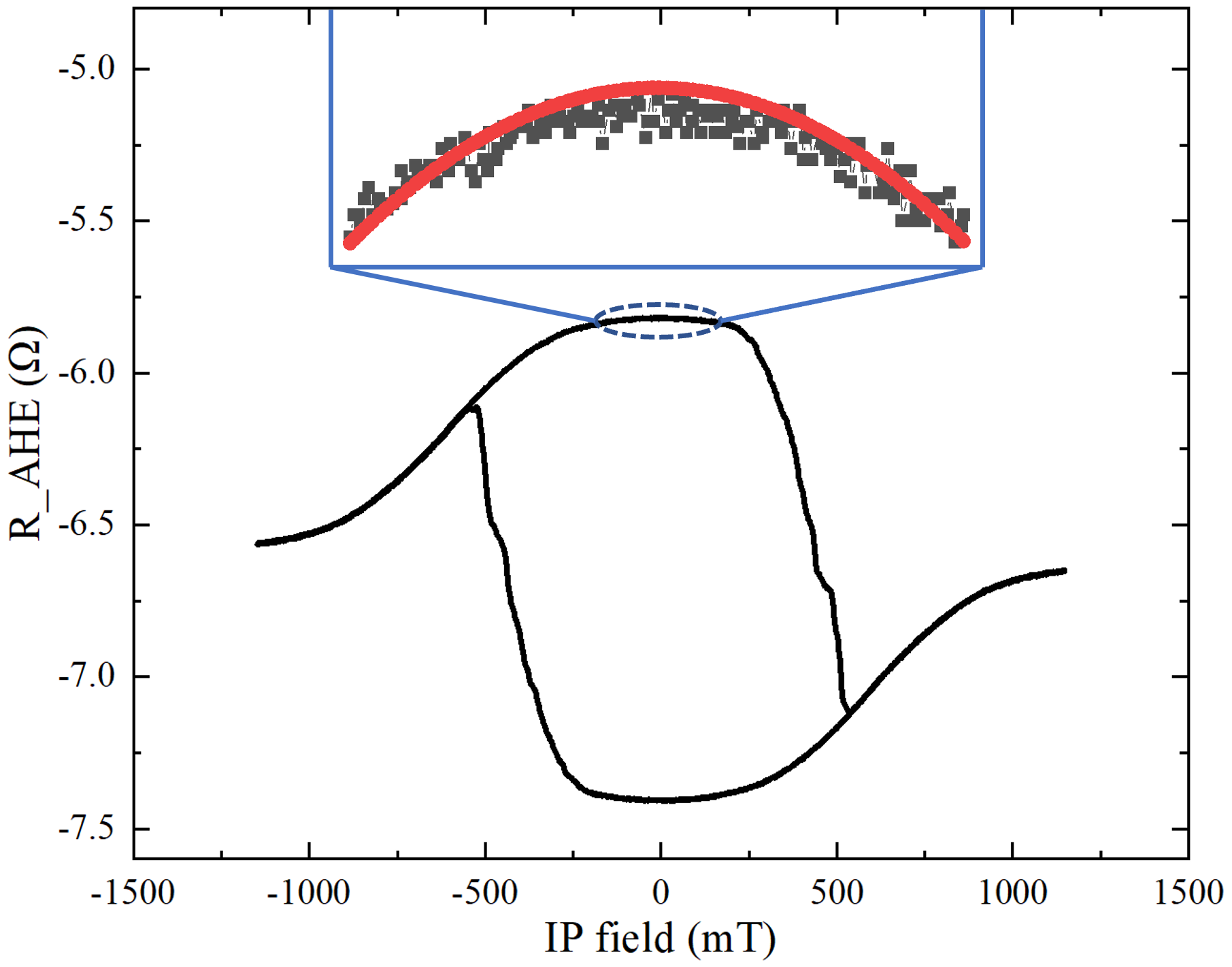


**Supplementary** **Figure S32. AHE curve of the reference device Ta(30)/Pt(30)/Co(9)/Ir(14.5) under in-plane field.**

With the experimentally obtained values of *Ms*​ and *K* at room temperature, we can use the above equations to describe their temperature dependence. Figure S33 shows the temperature dependence of the saturation magnetization and interfacial anisotropy. Here, $d_{th}$ represents the rate at which magnetic anisotropy decreases with increasing temperature. A larger $d_{th}$ indicates a more rapid reduction in anisotropy as the temperature rises. The temperature-dependent values of *Ms* ​and *K* are input into Mumax simulations to model transient SOT switching. Figure S34 shows the analog-like SOT switching results under different *dₜₕ* values. Here, we selected *dₜₕ* = 0.4, as its simulation curve best matches the experimental results. Moreover, this value is consistent with previously reported results [18].


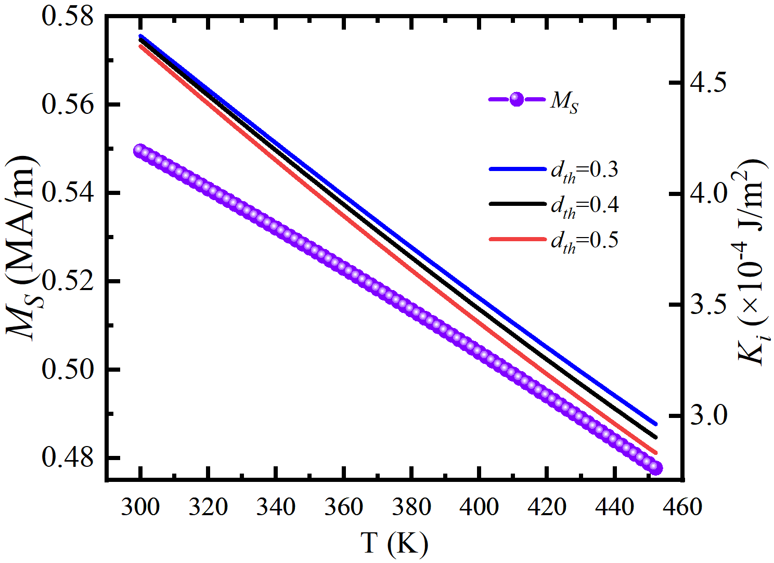


**Supplementary** **Figure S33. Temperature dependence of the saturation magnetization (left axis) and interfacial anisotropy (right axis).**


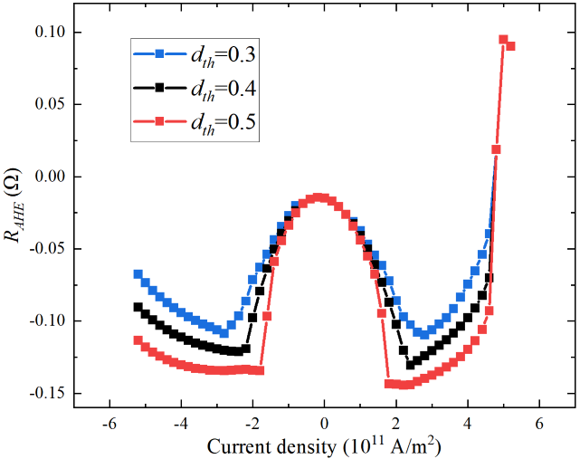


**Supplementary** **Figure S34. Simulated analog-like SOT switching with different** $\boldsymbol{d}_{\boldsymbol{th}}$**.**

**S19. Details and training process of the SNN constructed using analog-like SOT switching devices as neurons.**

In mainstream SNNs, a typical ideal LIF neuron is implemented using an RC circuit, which consists of a resistor and a capacitor. The differential equation of the LIF model directly corresponds to the charging and discharging process of the RC circuit: $\tau_{m}\frac{du}{dt}=-u+R_{m}I\left( t \right), \tau_{m}=R_{m}C_{m}$. The leaky term $-u$ models the leakage current through ion channels, analogous to the natural discharge in an RC circuit. The integration term $R_{m}I\left( t \right)$ simulates the effect of input current on charging the membrane potential. When the membrane potential $u\geq u_{th}$​, the circuit emits a spike and resets the potential, resembling the discharge of a capacitor.

The fundamental difference between an ideal neuron and our analog SOT-based LIF neuron lies in their underlying physical models: the RC circuit model versus the analog magnetization switching model. Accordingly, their specific differences in an SNN are as follows:

1) Different model parameters in the LIF framework (reflected in the leaky constant $\lambda$, firing threshold $\nu$, and output spike $o$ in the formula in figure 5);

2) Variations in the timing, frequency, and amplitude of synaptic spike inputs resulting from the differing LIF models;

3) Energy consumption differences arising from the distinct physical mechanisms. In our system, the firing mechanism of the LIF neuron is automatically achieved by the threshold switching of the SOT device, which does not require additional detection circuit as implemented in other systems and designs [25].

The core parameters of the LIF model directly influence the dynamics and information processing capabilities of SNNs, thereby affecting the model's recognition accuracy. For example, a larger leak constant $\lambda$ makes the neuron more sensitive to historical inputs but may lead to noise accumulation. In contrast, a smaller $\lambda$ is better suited for high-frequency spike inputs. Different $\lambda$ is preferable in different training scenarios. A higher firing threshold $\nu$ reduces the spike emission frequency, suppressing noise but potentially leading to signal sparsity and decreased accuracy. Conversely, a lower $\nu$ improves information transmission efficiency but may result in over-activation. To perform the real-world task using the IL-DMI-assisted SOT switching behavior, we have extracted the hyperparameters such as the leaky constant λ from the characteristics of the device. To clarify, the testing accuracy of 92.5% achieved after 25 epochs was computed using a model based on the LIF neuron dynamics, where the parameters for the LIF model were extracted from the behavior of our SOT device. The accuracy of the SOT-based neuron model, thus, reflects its performance when implemented using real device parameters. We add the training details below.

To derive a discrete, recurrent representation of the LIF neuron from the ordinary differential equation (ODE) describing the RC circuit:

$$\tau_{m}\frac{du}{dt}=-u+R_{m}I\left( t \right),$$

Where $u$ is the membrane potential, $I\left( t \right)$ is the input current, and $\tau_{m}=R_{m}C_{m}$ is the time constant of the circuit. The forward Euler method is used to solve the above linear ODE, providing a good enough approximation of continuous-time integration. This gives a good enough approximation of continuous-time integration. By isolating the membrane potential at the following time step, the equation becomes:

$$u\left( t+\Delta t \right)=\left( 1-\frac{\Delta t}{\tau_{m}} \right)u\left( t \right)+I\left( t \right)=\lambda u\left( t \right)+wI(t),$$

If a spike is triggered, the membrane potential should be reset. The reset-by subtraction mechanism is modeled by:

$$u\left( t+\Delta t \right)=\lambda u\left( t \right)+wI\left( t \right)-vS(t),$$

Where the neuron emits an output spike if the membrane exceeds the threshold $v$:

$$S\left( t \right)=\left\{ \begin{aligned} 1, if u\left( t \right)>v \\ 0, otherwise \end{aligned} \right.$$

For a specific neuron node in a spiking neural network, the weighted sum of the input current is taken as overall inputs:

$$u_{i}\left( t+\Delta t \right)=\lambda u_{i}\left( t \right)+\sum_{j} w_{ij}I\left( t \right)-vS(t),$$

Where $w_{ij}$ is learnable parameter, and *v* is often set to 1 (can be tuned), this leaves the $\lambda$ as the primary hyperparameter to be specified.

The parameter $\lambda$ (set to 0.28) was extracted from the characteristics of the device and integrated the model for training using the surrogate gradient method. Specifically, we use the spikingjelly pakage [26] for the SNN training on the MNIST dataset, which contains 60,000 training images and 10,000 testing images. In our experiment, the simulating time step T was set to 100, the batch size was set as 64, and the ADAM optimier was used for the training. Fig. S35 shows the respective training and testing results for our SNN.


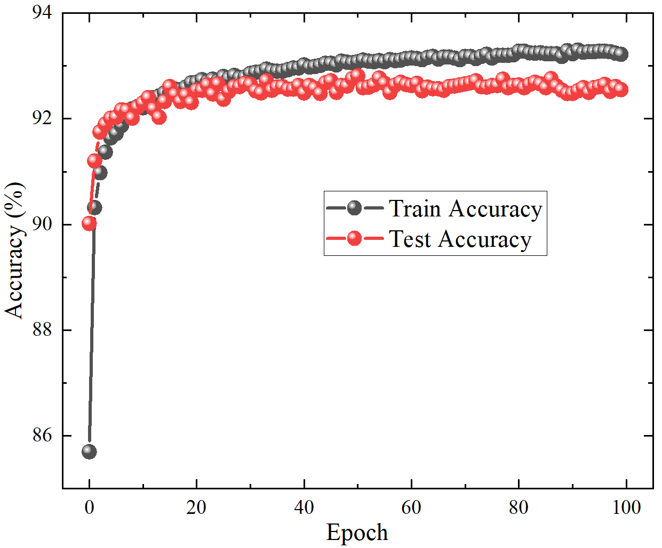


**Supplementary** **Figure S35. The train and test accuracy of MNIST digital handwritten patterns recognition as a function of learning epochs.**

**S20.** **Discussion on the LIF neuron model and SNN test accuracy**

Here, the experimental and simulation results of the AHE resistance as a function of SOT current in this study both confirm its functional characteristics for neuron applications. Based on this behavior, the physical mechanisms required in the SFi structure include: (i) IL-DMI and thermal effects that jointly drive the gradual magnetization tilting (integrate), (ii) strong perpendicular anisotropy and post-pulse thermal relaxation that restore the magnetization (leaky), and (iii) SOT-induced switching (fire). All three ingredients are essential.

1. In our experiments, transient resistance measurements are performed and the device resistance is read immediately after current input. Taking the switching result under an in-plane field of +10 mT (Fig. S36 below) an example, the SOT-induced switching curve naturally exhibits the leaky-integrate-and-fire (LIF) characteristics of a neuron: the device resistance increases progressively with current density (integrate), resets to a low-resistance antiparallel state once the current is removed (leaky), and undergoes switching when a threshold is exceeded (fire). Compared to traditional binary switching curves, our analog-like switching demonstrates, for the first time, the leaky behavior of neurons. The only difference is that the switching in this case is triggered by accumulated current density, rather than by an increasing number of discrete pulses. However, numerous studies have confirmed that in SOT switching, the multi-resistance switching behavior induced by variations in current density is consistent with that induced by different numbers of pulses [27][28]. Some studies have even directly applied the switching behavior caused by current density variations to neural networks [29]. Therefore, we believe that the analog-like SOT switching can qualitatively confirm the LIF characteristics of neurons in this context.


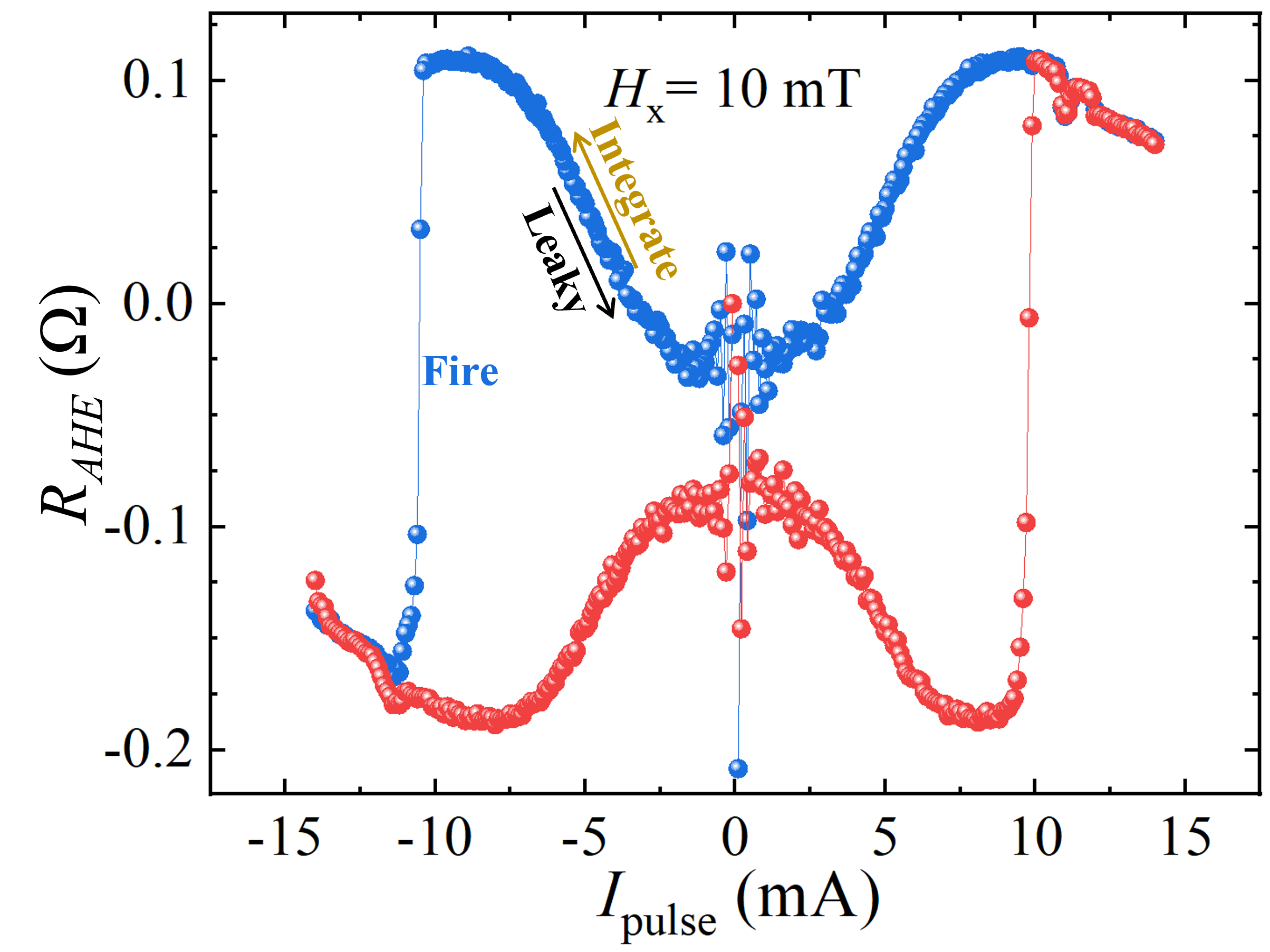


**Supplementary** **Figure S36. The inherent leaky, integrate and fire characteristics exhibited in the analog-like SOT switching.**

1. Our micromagnetic simulation results accurately reproduce the analog SOT switching behavior observed experimentally. We also fully supplemented the direct correlation between the simulation and experimental results, as detailed in S17. The simulations are not independent; parameters such as saturation magnetization and magnetic anisotropy are extracted directly from the experimental devices. The analog-type switching behavior is well reproduced, and the threshold switching current (4.8×10^11^ A/m^2^ in Fig 5b), after considering thermal effects, is also very close to the experimental results (3.8×10^11^ A/m^2^). Therefore, we believe that the analog-like SOT switching from simulation can quantitatively confirm the LIF characteristics of neurons in this context.

Here, we summarize the challenges that need to be addressed in order to realize LIF neurons experimentally with analog-like SOT switching deivces and implement them into SNN:

1) Device scaling and structural optimization. The device size needs to be scaled down from the micrometer to the nanometer regime, and the structure should evolve from a Hall bar to a magnetic tunnel junction (MTJ). In Supplementary Material 5, we calculated the energy consumption per spike for a single pulse. The current micrometer-scale device consumes 3.3 nJ/spike. After scaling down to the nanometer scale, the required thermal assistance for switching will be significantly reduced, and theoretical estimates suggest that the energy consumption can be as low as 1.2 fJ/spike. Moreover, the IL-DMI originates from the spacer layer in SAFs, which are already widely used in MTJs. This indicates high compatibility between our device and MTJ structures. Extending the device structure to MTJs can improve network convergence speed, reduce sensitivity to noise, enhance stability and accuracy, and optimize energy consumption.

2) System-level considerations for MTJ arrays targeting SNN applications. To implement arrays of MTJ devices for SNNs, we also need to address challenges related to device uniformity, interconnection, and compatibility with CMOS peripheral circuitry. Taking MNIST handwritten digit recognition as an example, the threshold current and TMR of the MTJ devices must be kept with minimal variation to achieve higher training accuracy. Additionally, algorithmic control is required to manage the network’s dynamic behavior, correcting unstable phenomena such as overactivation or suppression. This involves optimizing the network structure to enhance both performance and energy efficiency, thereby enabling co-optimization between hardware and algorithms.

Regarding the recognition accuracy, Table S2 lists the recognition rates of current state-of-the-art neuromorphic systems based on spintronic devices.

Table S2 State-of-the-art neuromorphic systems based on spintronic devices

| Category | Device type | NN | Device structure | Origin | Test Accuracy | Function |
| --- | --- | --- | --- | --- | --- | --- |
| Spin synapse | MTJ based on domain wall motion | ANN | CoFeB/MgO/CoFeB/NiFe  FeB/MgO/FeB/Ta/FeB | [30][31] | \ | Memristors simulating synaptic functions |
|  | MTJ based on domain wall motion | ANN | CoFeB/MgO/ CoFeB | [32] | 93% | Synaptic function simulation and MINST recognition |
|  | MTJ with different skyrmion number | ANN | Pt/GdFeCo/MgO | [33] | 89% | Synaptic function simulation and pattern recognition |
|  | MTJ based on multi-state SOT switching | ANN | PtMn/Pt/[Co/Ni]_2_/Co/MgO  Ta/GdFeCo/Ta | [34][35] | 92.25% | Memristors simulating long-term potentiation and depression |
|  | MTJ based on multi-state SOT switching | SNN | PtMn/Pt/[Co/Ni]_2_/Co/MgO  MgO/CoFeB/W/CoFeB/ MgO | [16][36] | \ | Memristors simulating synaptic functions and STDP |
|  | MTJ based on multi-state switching | ANN | Mn_3_Sn/Pt/Mn/AlN | [37] | 97.5% | Synaptic function simulation and facial recognition |
|  | MTJ based on multi-state SOT switching | ANN | CoFeB/Pt/Co/Pt | [38] | 92.2% | Memristors simulating synaptic functions |
| Spin neuron | superparamagnetic MTJ | ANN | Low energy barrier magnet | [39] | \ | Simulating the Sigmoid nonlinear function |
|  | Ferrimagnetic MTJ based on multi-state SOT switching | ANN | Pt/CoGd/Ta | [40] | 93% | Simulating the Sigmoid nonlinear function |
|  | MTJ based on voltage modulation | ANN | CoFeB/MgO/CoFeB/Ru/CoFe /PtMn | [41] | 95.5% | Simulating the Sigmoid nonlinear function and MINST recognition |
|  | MTJ based on domain wall motion | SNN | W/CoFeB/MgO/ CoFeB | [10] | 88.5% | Simulating LIF function with a reset function |
|  | MTJ based on skyrmion motion | SNN | Micromagnetic simulation | [42][43] | \ | Simulating LIF function |
|  | Magnetization dynamics based on SOT | SNN | PtMn/Pt/[Co/Ni]_2_/Co/MgO | [16] | \ | Simulating stochastic LIF function |
|  | Magnetization dynamics based on SOT | SNN | Pt/Co/Ir/Co/Pt | Our work | 92.5% | Simulating deterministic LIF function |
| All-Spin device | MTJ based on domain wall motion | ANN | W/CoFeB/MgO/ CoFeB/W | [44] | \ | domain wall linear/nonlinear pinning |
|  | MTJ based on multi-state SOT switching | ANN | Pt/CoGd/Ta  MgO/FePt/TiN/NiFe | [40][45] | 93% | simulating synaptic functions and the Sigmoid nonlinear function |
|  | MTJ based on multi-state SOT switching | ANN | Simulation | [46] | 91% | MINST recognition |
|  | MTJ-based Spin oscillator | ANN | CoFeB/CoFe/MgO/ FeB/MgO | [47] | 98% | Spoken digital recognition |

From the table, it can be observed that most neuromorphic systems based on spintronic devices exhibit recognition accuracies in the range of 80% to 90%. The recognition accuracy of 92.5% achieved by our device falls within the intermediate range. At the algorithm and system level, further performance gains can be achieved by exploring more advanced network structures and training methodologies. In this work, we deliberately adopt a simple two-layer SNN topology to isolate and highlight the deterministic LIF behavior enabled by IL-DMI-assisted SOT switching. From a system and algorithm perspective, however, the same physical neuron model can be naturally extended to more sophisticated SNN architectures. For example, introducing one or more hidden LIF layers, or incorporating a spiking convolutional front-end followed by spintronic LIF readout neurons, would allow the network to extract hierarchical features and is known to significantly improve MNIST classification accuracy in software implementations. With hardware-aware surrogate-gradient training, layer-wise normalization, and device-calibrated neuron models, similar accuracy improvements are expected in our spintronic SNN framework. Exploring such deeper and more advanced spintronic SNN architectures represents a promising direction for future work and may enable even higher accuracy and scalability in neuromorphic computing systems.

In the context of current popular SOT research, whether in artificial neural networks (ANNs) or spiking neural networks (SNNs), previous neuron-oriented implementations have primarily relied on stochastic switching by tuning the input current magnitude. These probabilistic switching behaviors are then fitted to a Sigmoid function to enable nonlinear signal processing. In contrast, our work extends the application of SOT-based devices for neuromorphic computing by demonstrating, for the first time, a deterministic LIF neuron. This analog SOT switching behavior opens a new direction for neuromorphic applications, as deterministic LIF neurons eliminate the need for multiple iterations, and have been shown to enable neural networks with higher sparsity, improved accuracy and superior energy efficiency [48].

**S21. Energy consumption quantification and performance comparison of analog-like SOT devices applied to neurons of SNN.**

SNNs can currently be implemented on two mainstream types of processors: one is the classical digital circuitry such as Intel CPUs and FPGAs; the other is neuromorphic manycore processors, including TrueNorth, Loihi, and Braindrop [49]. To characterize the energy efficiency of neural networks, the energy consumption per synaptic event—measured in joules per spike (J/spike)—is commonly used. The width and amplitude of spikes are determined based on the requirement that two superimposed spikes should induce the maximum change in the device state [16]. For classical digital processors, power consumption typically ranges from several hundred milliwatts to 2 W. SNNs implemented on FPGAs generally consume more than 2×10^6^ fJ/spike [50]. In contrast, neuromorphic processors offer significantly lower energy consumption: TrueNorth consumes 26000 fJ per synaptic event [51][52]; Loihi, as reported in [53], consumes 23600 pJ/spike; and Braindrop achieves ultra-low energy usage of just 381 fJ per synaptic event [54].

Regarding our analog-like switching device, the supplementary figure S24 shows a multi-resistance-state switching in a Hall bar device with dimensions of 4 μm × 50 μm, corresponding to the implementation of multi-level, non-volatile synaptic functionality. Here, we select a switching curve with a pulse width of 25 μs to represent a single synaptic event (ΔR>0.08 Ω). With a device resistance of 110 Ω and a threshold switching voltage of 0.12 V, the energy consumption per spike is calculated to be $V^{2}t/R$=3.3 nJ. The current density required for SOT switching is theoretically size-independent, while the thermal effects associated with switching are expected to decrease significantly as the device dimensions are scaled down. In our experiment, the measured threshold current for SOT-induced switching is 11.5 mA (Fig 3d in the main text), with a device cross-sectional area of 10 μm × 3 nm. The resulting current density (3.8×10^11^ A/m^2^) closely matches the threshold current density observed in our simulations (4.8×10^11^ A/m^2^ in Fig 5b). Considering device scaling down to the nanoscale, our micromagnetic simulations confirm that the energy consumption of the device (with dimensions of 40 nm × 1000 nm and resistance estimated by proportionally scaling experimental data) is $I^{2}Rt=$3.24 fJ per spike. The energy consumption per spike of our neuron, constructed using analog-like SOT switching, is 2–4 orders of magnitude lower than that of current neuromorphic manycore processors, and is comparable to the energy cost per synaptic event at the brain level, which ranges from 1 to 50 fJ [55].

**REFERENCES**

1. Krause S, Herzog G, Stapelfeldt T et al. Magnetization Reversal of Nanoscale Islands: How Size and Shape Affect the Arrhenius Prefactor. Phys Rev Lett 2009;103:127202.
2. Fache T. Iridium-based synthetic ferrimagnets for spintronics[D]. Université de Lorraine, 2020.
3. Luo X, Wang Y, Liu S et al. Unusual spin–orbit torque switching in perpendicular synthetic antiferromagnets with strong interlayer exchange coupling. J Phys: Condens Matter 2023;35:264004.
4. Bruno P, Bayreuther G, Beauvillain P et al. Hysteresis properties of ultrathin ferromagnetic films. J Appl Phys 1990;68:5759–66.
5. Morgunov RB, Kunitsyna EI, Talantsev AD et al. Influence of the magnetic field sweeping rate on magnetic transitions in synthetic ferrimagnets with perpendicular anisotropy. Appl Phys Lett 2019;114:222402.
6. Lee K-S, Lee S-W, Min B-C et al. Threshold current for switching of a perpendicular magnetic layer induced by spin Hall effect. Appl Phys Lett 2013;102:112410.
7. Han D-S, Lee K, Hanke J-P et al. Long-range chiral exchange interaction in synthetic antiferromagnets. Nat Mater 2019;18:703–8.
8. Avci CO, Lambert C-H, Sala G, Gambardella P. Chiral Coupling between Magnetic Layers with Orthogonal Magnetization. Phys Rev Lett 2021;127:167202.
9. Wang Z, Li P, Fattouhi M et al. Field-free spin-orbit torque switching of synthetic antiferromagnet through interlayer Dzyaloshinskii-Moriya interactions. Cell Reports Physical Science 2023;4:101334.
10. Wang D, Tang R, Lin H et al. Spintronic leaky-integrate-fire spiking neurons with self-reset and winner-takes-all for neuromorphic computing. Nat Commun 2023;14:1068.
11. Huang Y-H, Han J-H, Liao W-B et al. Tailoring Interlayer Chiral Exchange by Azimuthal Symmetry Engineering. Nano Lett 2024;24:649–656.
12. Vansteenkiste A, Leliaert J, Dvornik M et al. The design and verification of MuMax3. AIP Adv 2014;4:107133.
13. Shahbazi K, Kim J-V, Nembach HT et al. Domain-wall motion and interfacial Dzyaloshinskii-Moriya interactions in Pt / Co / Ir ( t Ir ) / Ta multilayers. Phys Rev B 2019;99:094409.
14. Guo Z, Yin J, Bai Y et al. Spintronics for Energy- Efficient Computing: An Overview and Outlook. Proc IEEE 2021;109:1398–417.
15. Pai C-F, Liu L, Li Y et al. Spin transfer torque devices utilizing the giant spin Hall effect of tungsten. Appl Phys Lett 2012;101:122404.
16. Kurenkov A, DuttaGupta S, Zhang C et al. Artificial Neuron and Synapse Realized in an Antiferromagnet/Ferromagnet Heterostructure Using Dynamics of Spin–Orbit Torque Switching. Adv Mater 2019;31:1900636.
17. Prejbeanu IL, Bandiera S, Alvarez-Hérault J et al. Thermally assisted MRAMs: ultimate scalability and logic functionalities. J Phys D: Appl Phys 2013;46:074002.
18. Li S, Lv C, Lin X et al. Phase-change-assisted spin-transfer torque switching in perpendicular magnetic tunnel junctions. Appl Phys Lett 2021;119:122401.
19. O. Murtagh, B. Walls, I. V. Shvets, Applied Physics Letters 2020, 117, 063501.
20. Zhao W, Duval J, Klein J-O, Chappert C. A compact model for magnetic tunnel junction (MTJ) switched by thermally assisted Spin transfer torque (TAS + STT). Nanoscale Res Lett 2011;6:368.
21. Razavi SA, Wu D, Yu G et al. Joule Heating Effect on Field-Free Magnetization Switching by Spin-Orbit Torque in Exchange-Biased Systems. Phys Rev Applied 2017;7:024023.
22. Sato H, Chureemart P, Matsukura F et al. Temperature-dependent properties of CoFeB/MgO thin films: Experiments versus simulations. Phys Rev B 2018;98:214428.
23. Lequeux S, Perrissin N, Grégoire G et al. Thermal robustness of magnetic tunnel junctions with perpendicular shape anisotropy. Nanoscale 2020;12:6378–84.
24. Zhang K, Chen L, Zhang Y et al. Efficient and controllable magnetization switching induced by intermixing-enhanced bulk spin–orbit torque in ferromagnetic multilayers. Applied Physics Reviews 2022;9:011407.
25. Bouvier M, Valentian A, Mesquida T et al. Spiking Neural Networks Hardware Implementations and Challenges: A Survey. J Emerg Technol Comput Syst 2019;15:1–35.
26. Fang W, Chen Y, Ding J et al. SpikingJelly: An open-source machine learning infrastructure platform for spike-based intelligence. Sci Adv 2023;9:eadi1480.
27. Zhang S, Luo S, Xu N et al. A Spin–Orbit‐Torque Memristive Device. Adv Elect Materials 2019;5:1800782.
28. Cao Y, Rushforth AndrewW, Sheng Y, Zheng H, Wang K. Tuning a Binary Ferromagnet into a Multistate Synapse with Spin–Orbit‐Torque‐Induced Plasticity. Adv Funct Materials 2019;29:1808104.
29. Zhou J, Zhao T, Shu X et al. Spin–Orbit Torque‐Induced Domain Nucleation for Neuromorphic Computing. Advanced Materials 2021;33:2103672.
30. Chanthbouala A, Matsumoto R, Grollier J et al. Vertical-current-induced domain-wall motion in MgO-based magnetic tunnel junctions with low current densities. Nature Phys 2011;7:626–30.
31. Lequeux S, Sampaio J, Cros V et al. A magnetic synapse: multilevel spin-torque memristor with perpendicular anisotropy. Sci Rep 2016;6:31510.
32. Liu S, Xiao TP, Cui C et al. A domain wall-magnetic tunnel junction artificial synapse with notched geometry for accurate and efficient training of deep neural networks. Applied Physics Letters 2021;118:202405.
33. Song KM, Jeong J-S, Pan B et al. Skyrmion-based artificial synapses for neuromorphic computing. Nat Electron 2020;3:148–55.
34. Borders WA, Akima H, Fukami S et al. Analogue spin–orbit torque device for artificial-neural-network-based associative memory operation. Appl Phys Express 2017;10:013007.
35. Hu H, Wang K, Li W et al. Spin–orbit torque-induced memristor in Ta/GdFeCo/Ta structures for neuromorphic computing. Journal of Magnetism and Magnetic Materials 2024;589:171582.
36. Zhang X, Cai W, Wang M et al. Spin‐Torque Memristors Based on Perpendicular Magnetic Tunnel Junctions for Neuromorphic Computing. Adv Sci 2021;8:2004645.
37. Zheng Z, Zeng T, Zhao T et al. Effective electrical manipulation of a topological antiferromagnet by orbital torques. Nat Commun 2024;15:745.
38. Lin C-Y, Hsieh J-Y, Wang P-C, Tsai C-C, Pai C-F. Field-free spin–orbit torque devices for logic and neural network applications. APL Machine Learning 2024;2:046110.
39. Camsari KY, Sutton BM, Datta S. p-bits for probabilistic spin logic. Applied Physics Reviews 2019;6:011305.
40. Liu J, Xu T, Feng H et al. Compensated Ferrimagnet Based Artificial Synapse and Neuron for Ultrafast Neuromorphic Computing. Adv Funct Materials 2022;32:2107870.
41. Cai J, Fang B, Zhang L et al. Voltage-Controlled Spintronic Stochastic Neuron Based on a Magnetic Tunnel Junction. Phys Rev Applied 2019;11:034015.
42. Chen X, Kang W, Zhu D et al. A compact skyrmionic leaky–integrate–fire spiking neuron device. Nanoscale, 2018;10:6139–46.
43. Li S, Kang W, Huang Y et al. Magnetic skyrmion-based artificial neuron device. Nanotechnology 2017;28:31LT01.
44. Liu L, Wang D, Wang D et al. Domain wall magnetic tunnel junction-based artificial synapses and neurons for all-spin neuromorphic hardware. Nat Commun 2024;15:4534.
45. Dong K, Guo Z, Jiao Y et al. Field-Free Current-Induced Switching of L 1 0 - Fe Pt Using Interlayer Exchange Coupling for Neuromorphic Computing. Phys Rev Applied 2023;19:024034.
46. Cao Z, Zhang S, Hou J, Duan W, You L. All-Spin Artificial Neural Network Based on Spin–Orbit Torque-Induced Magnetization Switching. IEEE Trans Electron Devices 2023;70:6336–40.
47. Torrejon J, Riou M, Araujo FA et al. Neuromorphic computing with nanoscale spintronic oscillators. Nature 2017;547:428–31.
48. Jiang Y, Lu S, Sengupta A. Stochastic Spiking Neural Networks with First-to-Spike Coding. 2024 International Conference on Neuromorphic Systems (ICONS) IEEE 2024;24–31.
49. Wang S, Maris Ferreira P, Benlarbi-Delai A. Physics Informed Spiking Neural Networks: Application to Digital Predistortion for Power Amplifier Linearization. IEEE Access 2023;11:48441–53.
50. John Wood, Behavioral Modeling and Linearization of RF Power Amplifiers, Artech, 2014.
51. Merolla PA, Arthur JV, Alvarez-Icaza R et al. A million spiking-neuron integrated circuit with a scalable communication network and interface. Science 2014;345:668–73.
52. Aguirre F, Sebastian A, Le Gallo M. et al. Hardware implementation of memristor-based artificial neural networks. Nat Commun 2024;15:1974.
53. Davies M, Srinivasa N, Lin T-H et al. Loihi: A Neuromorphic Manycore Processor with On-Chip Learning. IEEE Micro 2018;38:82–99.
54. Neckar A, Fok S, Benjamin BV et al. Braindrop: A Mixed-Signal Neuromorphic Architecture With a Dynamical Systems-Based Programming Model. Proc IEEE 2019;107:144–64.
55. Laughlin SB, de Ruyter van Steveninck RR, Anderson JC. The metabolic cost of neural information. Nature Neuroscience 1998;1:36–41.
